# Novel autoproteolytic and DNA-damage sensing components in the bacterial SOS response and oxidized methylcytosine-induced eukaryotic DNA demethylation systems

**DOI:** 10.1186/1745-6150-8-20

**Published:** 2013-08-15

**Authors:** L Aravind, Swadha Anand, Lakshminarayan M Iyer

**Affiliations:** 1 National Center for Biotechnology Information, National Library of Medicine, National Institutes of Health, Bethesda, MD 20894, USA

## Abstract

**Abstract:**

The bacterial SOS response is an elaborate program for DNA repair, cell cycle regulation and adaptive mutagenesis under stress conditions. Using sensitive sequence and structure analysis, combined with contextual information derived from comparative genomics and domain architectures, we identify two novel domain superfamilies in the SOS response system. We present evidence that one of these, the SOS response associated peptidase (SRAP; Pfam: DUF159) is a novel thiol autopeptidase. Given the involvement of other autopeptidases, such as LexA and UmuD, in the SOS response, this finding suggests that multiple structurally unrelated peptidases have been recruited to this process. The second of these, the ImuB-C superfamily, is linked to the Y-family DNA polymerase-related domain in ImuB, and also occurs as a standalone protein. We present evidence using gene neighborhood analysis that both these domains function with different mutagenic polymerases in bacteria, such as Pol IV (DinB), Pol V (UmuCD) and ImuA-ImuB-DnaE2 and also other repair systems, which either deploy Ku and an ATP-dependent ligase or a SplB-like radical SAM photolyase. We suggest that the SRAP superfamily domain functions as a DNA-associated autoproteolytic switch that recruits diverse repair enzymes upon DNA damage, whereas the ImuB-C domain performs a similar function albeit in a non-catalytic fashion. We propose that C3Orf37, the eukaryotic member of the SRAP superfamily, which has been recently shown to specifically bind DNA with 5-hydroxymethylcytosine, 5-formylcytosine and 5-carboxycytosine, is a sensor for these oxidized bases generated by the TET enzymes from methylcytosine. Hence, its autoproteolytic activity might help it act as a switch that recruits DNA repair enzymes to remove these oxidized methylcytosine species as part of the DNA demethylation pathway downstream of the TET enzymes.

**Reviewers:**

This article was reviewed by RDS, RF and GJ.

## Introduction

The bacterial SOS, first described by Radman about 40 years ago, is a versatile stress-induced network for DNA repair, mutagenesis, cell cycle regulation and adaptation [1-4]. At its heart lies a DNA-damage sensor comprised of the LexA repressor, which combines a signal peptidase-like serine peptidase domain with a DNA-binding winged helix-turn-helix (wHTH) domain, and RecA, which is a P-loop ATPase that catalyzes strand exchange during homologous recombination [2,3]. Normally, the access of RecA to single stranded DNA is barred by the single-strand-binding protein. However, when DNA is damaged by single strand lesions, the RecFOR complex, and when by double-strand breaks, the RecBC complex, help RecA to gain access to DNA [5]. When RecA polymerizes as a filament on ssDNA it facilitates the peptidase domain of LexA to catalyze autoproteolysis. Autoproteolysis of LexA in turn allows transcription of genes repressed by it, thereby initiating the SOS response [1,2,4,6].

The many-faceted SOS network typically starts with induction of the UvrABD complex, which together with UvrC (which contains two endonuclease domains), mediates excision of nucleotides from damaged DNA [7]. This is followed by induction of homologous recombination networks and the cell division inhibitor SulA/SfiA (an inactive RecA paralog) that buys time for DNA repair to be completed. Temporal persistence of DNA damage induces the final module of the SOS network, i.e., DNA polymerase V (Pol V) comprised of UmuC and UmuD, a LexA paralog with comparable domain architecture [4,5,7]. Pol V can synthesize DNA over lesions, which normally stall the replicative polymerase III (Pol III), such as abasic sites, cyclobutane thymine dimers, and bases altered by radiation-induced reactions [4,8]. This allows the cell to potentially survive irreparable lesions but at a cost of increased mutagenesis due to the error prone nature of the polymerase. Induction of this mutagenic Pol V is strongly regulated: in order for the active polymerase complex to form, UmuD has to come in contact with RecA filaments and undergo autoproteolytic cleavage similar to LexA [4]. While absent in the *Escherichia coli*, a second SOS-induced mutagenic module has been reported across a wide phyletic range of bacteria in the form of the ImuA-ImuB-DnaE2 module [1,9,10]. The ImuA protein is an inactive paralog of RecA recombinase related to SulA, ImuB is an apparently catalytically inactive paralog of the DNA polymerase V catalytic subunit UmuC, and DnaE2 is an alternative, catalytically active Pol III α-subunit. These proteins form a distinct DNA repair complex that also appears to be capable of error-prone repair that requires the catalytic activity of DnaE2 [9,10].

Beyond induction of SOS by various genotoxic stresses, the SOS machinery also appears to play a role in facilitating mutagenesis and thereby adaptation to adverse conditions. For instance, presence of an unusable carbon source causes a subset of *E.coli* cells to undergo double-strand breaks that induces SOS [11]. Recombination across these break sites generates substrates for Pol IV (DinB), a SOS induced polymerase that synthesizes DNA across templates with bulged out nucleotides [12]. The resultant mutations might result in cells that become capable of using the previously inaccessible carbon source. Similar roles for SOS mutagenesis have also been postulated to be important in antibiotic resistance in bacteria [13]. The SOS response is also used by bacteriophages, whose lytic repressors can be cleaved by the LexA peptidase domain, to initiate the lytic cycle as a means of escaping from hosts which might be compromised [1].

Given the important role of the SOS response in bacterial adaptation, we sought to identify additional components that might throw light on the processes mediated by this network. Using comparative genomics and the extensive sequence data we identify two novel components of the SOS response that are likely to have a pan-bacterial role in regulating adaptive and repair-linked mutagenic processes. We also show that one of these components, an autopeptidase, has been transferred to eukaryotes, where, in certain lineages, it might function in recognition of modified cytosines as part of the DNA-damage response induced by the endogenous oxidation of methyl cytosine by the TET enzymes.

## Findings

### Identification of two novel components of the SOS network through gene-neighborhood analysis

**Figure 1:**
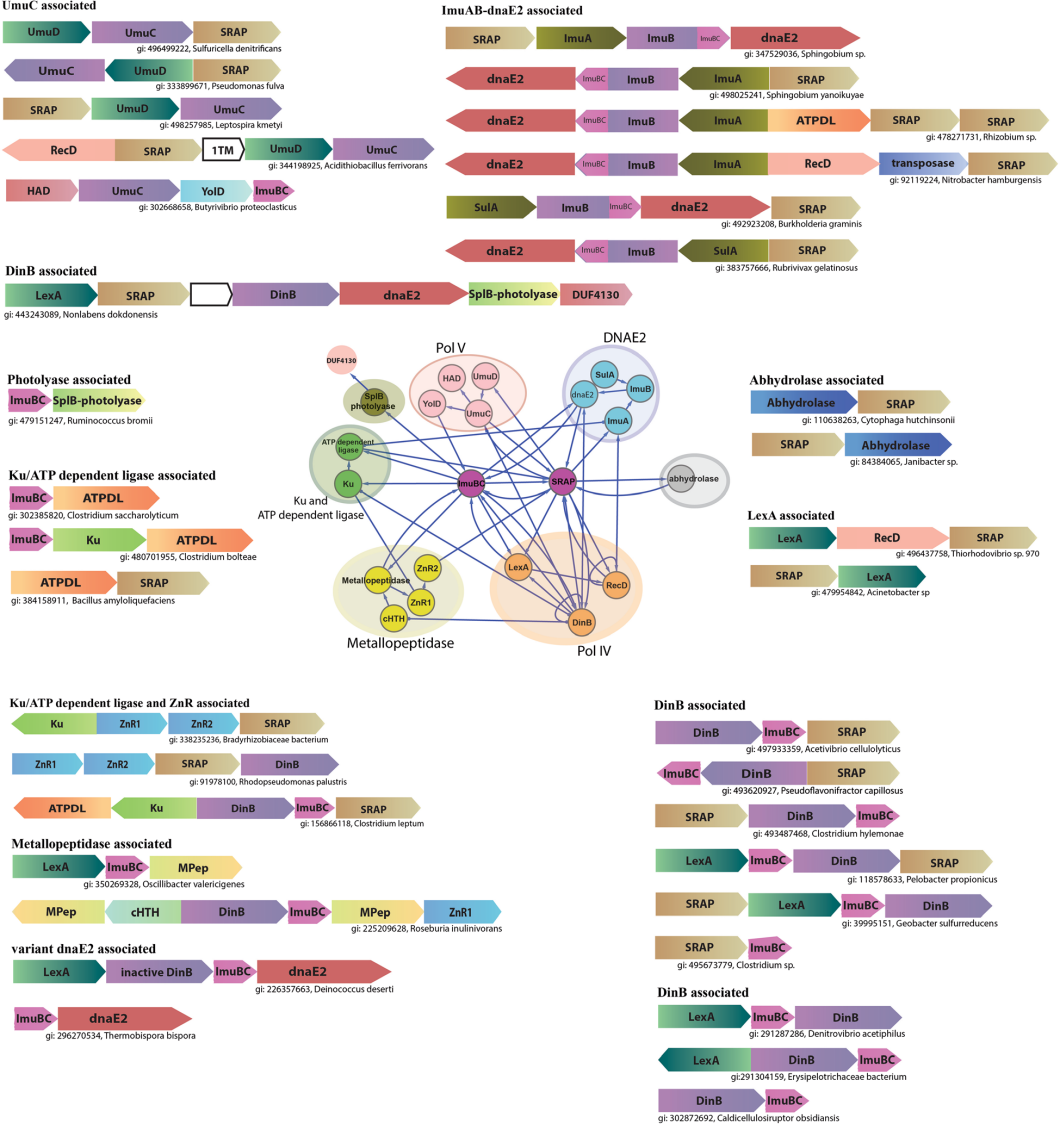
**Operons and contextual network graph of the SRAP and ImuB-C containing proteins.** Genes are represented as arrows, with the arrow head pointing from the 5’ to the 3’ direction. Operons are labeled with the gi number of the SRAP or ImuB-C gene in the neighborhood followed by the species name. Standard names of encoded domains are used to label genes, the rest being MPep, metallopeptidase; ATPDL, ATP-dependent DNA ligase. In the network graph, genes are represented as nodes, and edges indicate the gene context with the arrow head pointing to the gene in the 3’ direction of the neighborhood. Edges depicting genes arranged in diverging directions are shown with circular ends. Pfam DUF4130 is a predicted mutagenesis-related enzyme that is associated with the SplB-like radical SAM photolyases and uracil DNA glycosylases, which might modify thymines.

Previous studies on the key mutagenic components of the SOS response revealed two widely distributed types of conserved gene-neighborhoods: 1) the combination of UmuC and UmuD genes that respectively encode the catalytic and DNA-binding subunits of PolV [1,6,14,15]. 2) Those containing LexA, ImuA, ImuB, and DnaE2 [1,9,10]. While the complete versions of the latter operon (e.g. in *Pseudomonas putida*) contain all four genes, several organisms display more abbreviated or split versions of this with just two or three of the constituent genes [10]. Given the ever-increasing accumulation of genomic data we performed a new systematic collection of the gene-neighborhoods centered on these operon types by searching 11,123 bacterial genomes deposited in the Genbank database. We then clustered the previously uncharacterized, neighboring gene products to identify any novel, conserved components that might be associated with these operons. As a result we identified two uncharacterized superfamilies that tended to strongly co-occur with the mutagenic SOS operons across most major lineages of the bacterial tree (Figure 1 and Additional file 1; 25 and 65% of all their recovered gene neighborhood showed such associations). The first of these is typified by the *Bacillus subtilis* YoqW protein (gi: 384175670) and the *E.coli* protein YedK (gi: 49176171), and contains a globular domain of about 220-260 residues in length, which was previously described as containing a domain of unknown (DUF159) in the Pfam database [16].

The second of these codes for a protein prototyped by *Geobacter sulfurreducens* GSU0042 (gi: 39995153) and contains a previously unknown, small, globular domain approximately 80 residues in length. Iterative sequence profile searches with PSI-BLAST and hidden Markov model searches with HMMSEARCH using an alignment of different representatives of this domain showed that it was related to a conserved domain found in the ImuB family of proteins that occurs C-terminal to the family Y DNA polymerase domain. For instance, HMMSEARCH using a HMM derived from the small GSU0042-like proteins recovered the ImuB C-terminal domains with e-value ~10^-4^-10^-5^. Likewise, a search initiated using the *Syntrophomonas wolfei* protein Swol_1669 (gi: 114567184) recovered the ImuB C-terminal domains in a PSI-BLAST search (e-value = 10^-3^-10^-4^; iteration 7). Accordingly, we named this conserved domain the ImuB-C (for ImuB C-terminal) domain.

### Contextual and sequence-structure analyses reveal the DUF159 domain to be a DNA-associated autopeptidase

To better understand the role of the DUF159 proteins in the SOS response we carefully examined their gene contexts and found that they are encoded in at least three distinct operonic contexts related to the SOS response (Figure 1): 1) as neighbors of the UmuC-UmuD gene dyad that code for subunits of the translesion DNA PolV; 2) Alongside two or more genes that constitute the widespread mutagenic gene cluster namely ImuB, ImuA, and DnaE2 [1,9,10]; 3) As part of an operon with DinB (coding for translesion Pol IV). Exemplars of this gene-neighborhood might further include LexA [1] or genes for bacterial versions of the DNA end-binding Ku protein and the ATP-dependent DNA ligase [17] or in certain cases a DnaE paralog. Conserved gene-neighborhoods combining the DUF159 and Ku genes also code for two previously uncharacterized Zn-chelating domains that are predicted to adopt a Zn-ribbon-like fold (Figure 1 and Additional file 1). These observations suggested that these proteins are likely to functionally interact with three sub-networks of the SOS system that are dependent on different mutagenic polymerases and in some cases also with the end-joining repair system dependent on the Ku and the ATP-dependent ligase, along with additional partners such as the above Zn-chelating domains (Network in Figure 1). Additionally, DUF159 genes also co-occur in operons coding for a superfamily-I DNA helicase related to RecD (Figure 1). Given that these operons might additionally code for LexA and UmuCD, it is likely that this helicase is also part of the SOS associated DNA repair process.

**Figure 2:**
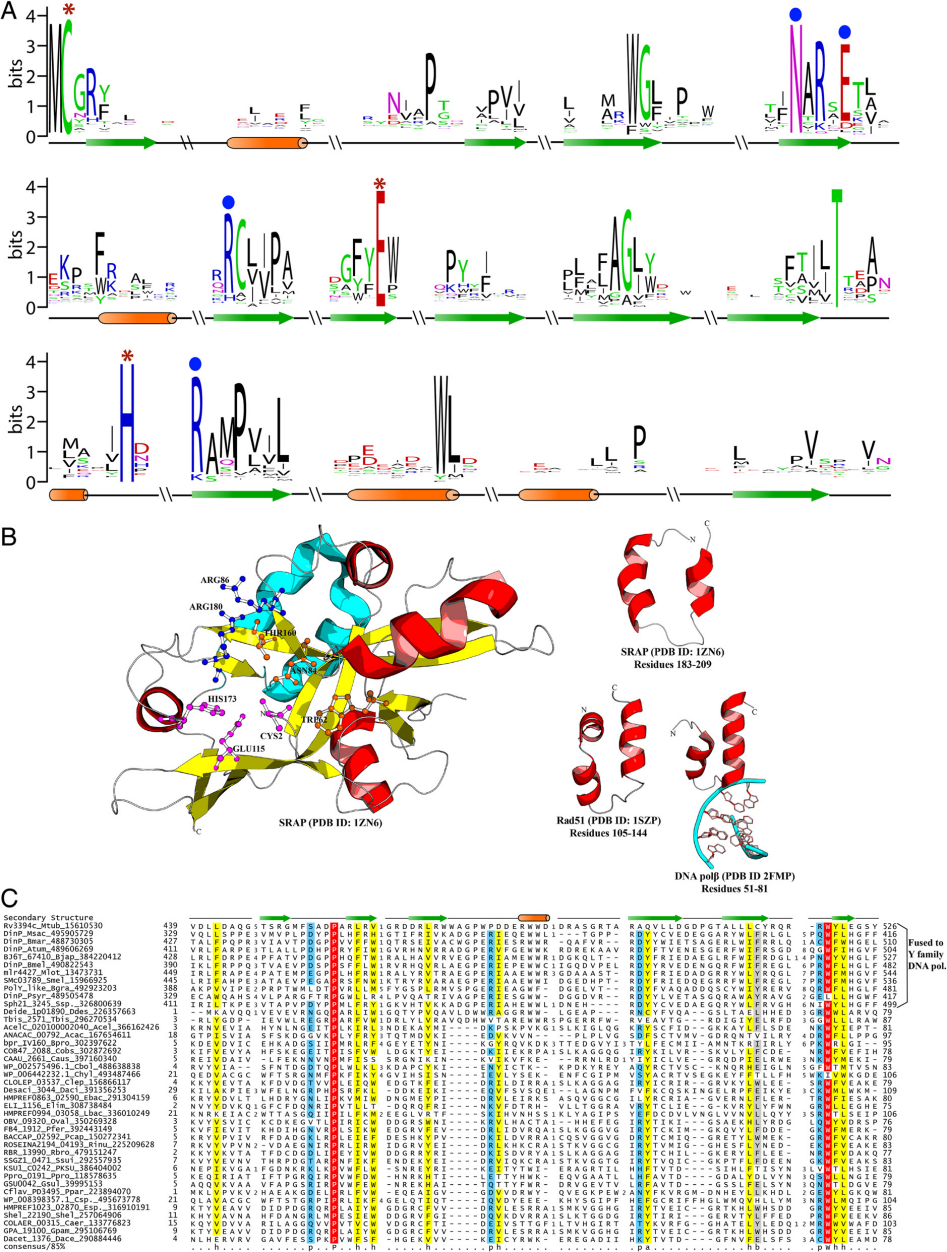
**Sequence conservation of the SRAP and ImuB-C domains, and structure of the SRAP domain. A)** Sequence logo for SRAP domain obtained from a multiple sequence alignments of representative sequences (See Additional file 1 for complete alignment). Residues predicted to catalyze auto-proteolysis are marked with red asterisks. Other conserved residues described in the text are marked with blue circles. **B)** A cartoon representation of the predicted active site for the SRAP protein is shown in left panel. The catalytic triad comprising of Cys2, His173 and E115 (PDB: 1zn6) has been depicted in magenta color, the arginine residues predicted to interact with DNA are shown in dark blue color, while the other conserved residues in active site are shown in orange. The predicted Helix-hairpin helix (HhH) region of SRAP domain (cyan) is shown in the right panel separately along with the HhH domains of RecA and the DNA polymerase β for comparison. **C)** Multiple sequence alignment of the ImuB-C domain. Proteins are labeled to the left of each sequence by their gene names, species abbreviations, and gi numbers separated by underscores. Amino acid residues are colored according to side chain properties and degree of conservation within the alignment, at 85% consensus. The secondary structure is indicated above the alignment with helices shown as orange cylinders and strands as green arrows. The consensus abbreviations and coloring scheme are as follows: h, hydrophobic (ACFGHILMTVWY) and a: aromatic (FYH) residues shaded yellow; polar (CDEHKNQRST) residues colored blue; big residues (QRKEILMWYF) shaded grey; absolutely conserved residues shaded red. Species abbreviations are provided in Additional file 1.

To further investigate the role of these proteins across distinct SOS-associated mutagenic processes we initiated multiple sequence profile and hidden Markov searches with the PSI-BLAST and JACKHMMER programs respectively and obtained a comprehensive collection of its members (Additional file 1). We then prepared a multiple sequence alignment and examined the sequence conservation patterns across the superfamily (Figure 2A). This revealed the presence of an absolutely conserved cysteine, which is always the second amino acid in the domain, followed by a conserved asparagine and glutamate in the central region of the domain, and a C-terminal histidine (Figure 2A; Additional file 1). Examination of the position of these residues in the multiple crystal structures of this superfamily, which have been solved as part of two distinct structural genomics efforts on uncharacterized proteins (PDB: 1zn6, 2f20, 2aeg, 2bdv and 2icu), revealed that the absolutely conserved histidine, glutamate and cysteine constitute a potential catalytic triad similar to those seen in structurally unrelated thiol peptidases (e.g. papain-like and caspase-hemoglobinase-like thiol peptidases; Figure 2A). This suggested that the domain might function as a thiol peptidase domain. Consistent with this prediction, all the five structures deposited in PDB have undergone autoproteolytic cleavage resulting in removal of the sequence N-terminal to the predicted catalytic cysteine (Figure 2B). Moreover, *E. coli* YedK was purified for crystallization with a longer tag fused to the N-terminus; this tag is also seen to be released by a similar cleavage N-terminal to the conserved cysteine in its crystal structure (PDB: 2icu). This suggests that the catalytic triad of these domains indeed catalyzes an autoproteolytic cleavage of N-terminal sequences, reminiscent of the autoproteolytic cleavage of LexA and UmuD, even though they are structurally and mechanistically unrelated [18]. Accordingly, we named this superfamily of domains the SOS response associated peptidase (SRAP), which reflects its function more appropriately. The structures also reveal that upon cleavage the above-mentioned highly conserved asparagine forms a hydrogen bond with the backbone of the catalytic cysteine resulting in it assuming a buried position (Figure 2B). Thus, it appears that the SRAP domains upon autoproteolysis assume a conformation that is no longer capable of further peptidase activity, suggesting that their autoproteolytic activity is part of a functional switch.

Examination of the structure of the SRAP domain reveals that it assumes a unique fold (BB1717-like fold in the SCOP database) that appears to have been constituted from 5 repeats of a β-hairpin [19]. Interspersed between these β-hairpins are α-helical regions that are similar to helix-hairpin-helix motifs (HhH; Figure 2B). Given that HhHs are prominent DNA-binding motifs especially common in DNA repair proteins [20], it is possible that the SRAP domain binds DNA. Furthermore, structures of the SRAP domain reveal that the catalytic triad and other highly conserved residues line a conserved structural pocket that could serve as a potential interaction site (Figure 2B). The periphery of this pocket contains two highly conserved arginines (R86 and R180, Figure 2B) that are also proximal to the HhH motif and could contact DNA. Consistent with these observations, the mammalian representative of this superfamily, C3Orf37, has been recently shown to bind DNA containing the oxidized derivatives of 5-methyl cytosine (hydroxymethyl cytosine, formyl cytosine and carboxy cytosine) in embryonic stem cells [21]. Oxidation of 5mC in DNA to these derivatives by the TET enzymes is important both as epigenetic marks and possibly as part of the demethylation pathway in mammalian stem cells [22]. Together these observations suggest that the SRAP domains function in close proximity with DNA and contain a conserved pocket, which could bind other proteins, perhaps in a proteolysis-dependent fashion.

### ImuB-C domains are functionally linked to diverse SOS-related DNA-repair proteins

The most frequently occurring version of the ImuB-C domains is seen linked to the C-terminus of the inactive family Y DNA polymerase domain in ImuB (Figure 1) [1,9,10]. Standalone versions of the ImuB-C domains are encoded in several distinct conserved gene-neighborhoods (Figure 1). Most frequently, it is combined with a gene for the translesion DNA polymerase IV (DinB). These operons also often code for LexA and SRAP superfamily genes, with more elaborate versions also containing genes for Ku and the ATP-dependent ligase [17]. The solo ImuB-C domain is also encoded by operons in combination with other translesion DNA-repair systems namely, DnaE2 (either with an inactive version of DinB or without it) and UmuC (along with the YolD gene family an earlier described but poorly understood SOS response factor [14]). Thus, ImuB-C appears to have functional connections with several major mutagenic DNA polymerase systems (network in Figure 1). It also occurs independently of these DNA polymerases in operons along with several other DNA repair proteins (Figure 1): 1) Ku and the ATP-dependent ligase; 2) SplB-like radical SAM superfamily DNA photolyases, previously implicated in the SOS response, which help repair 5-thyminyl-5,6-dihydrothymine, a specialized UV-induced thymine dimer [15,23]; 3) SRAP by itself; 4) Distinctive Zincin-like metallopeptidases (Pfam: DUF955 [16]). This metallopeptidase might mediate yet another SOS-linked proteolytic reaction akin to those catalyzed by LexA, UmuC and SRAP. Together the above observations suggest that the ImuB-C domain might not only interact with the mutagenic systems but also with other repair process related to the SOS response (Figure 1).

A multiple sequence alignment of the ImuB-C domain showed that it contains a highly conserved C-terminal tryptophan and at least two strongly conserved positively charged residues (Figure 2C); however, there are no indications of any pattern of conserved residues that might constitute a catalytic active site. While it could not be unified with a known fold, secondary structure prediction indicated that the ImuB-C domain is primarily composed of β-strands (Figure 2). The length of the domain and number of strands indicate that it could possibly adopt a SH3-like barrel fold. In light of above-described contextual connections, we propose that this domain is a potential DNA-binding domain, which either recognizes damaged DNA structures or photoproducts arising from radiation-exposed bases. Thus, it could help guide translesion polymerases and other DNA repair proteins to regions of DNA damage.

### General conclusions

Our findings indicate that peptidases with at least three unrelated structural folds and catalytic mechanisms, namely, signal peptidase-like serine-peptidases (LexA and UmuD), SRAP-like thiol peptidases and zincin-like metallopeptidases linked to ImuB-C and DinB (Pfam: DUF955) have been recruited for the SOS-associated DNA damage response. Of these at least LexA, UmuD and SRAP appear to possess autopeptidase activity. Moreover, proteolysis of UvrA, DinI, and RecN by the ClpPX system, of SulA/SfiA by the HslUV system, and of RecA and RuvB by the Lon system have also been implicated in regulation of the SOS response [18]. Thus, there appears to be a strong preference for proteolysis-dependent signaling over other signaling mechanisms in regulating the SOS response. One possible explanation for this could be the ability of proteolysis to directly convert conformational change into a signal without intermediate steps such as recognition of modified peptides or particular concentrations of soluble small molecules as seen in several other signaling pathways. Signaling via proteolysis might also allow for more sensitive discrimination between signal and noise, which could be of central importance in the decision process relating to deployment of potentially deleterious mutagenic polymerases.

To date the RecA protein has been studied as the primary sensor and trigger of the SOS response. However, the specific control of the temporally late-acting processes with potential to introduce mutations in the genome is less understood. Identification of the SRAP and ImuB-C domains as potential players in this process raises the possibility that these are regulators of the mutagenic arms of the SOS response. By specifically sensing types of DNA damage that could stall replication, these domains could potentially facilitate the action of translesion DNA polymerases either by autoproteolytic conformational change (SRAP) or direct recruitment (ImuB-C). Their deployment could represent checkpoints that need to be passed in order for the mutagenic processes to be activated. Thus, they could be important as regulators of “evolvability” via active mutagenesis under stress conditions. The above functional proposal might also explain the presence of SRAP in lysogenic bacteriophages: for instance, it is present in the temperate *Bacillus* phage SPBc2 and integrated prophages of proteobacteria, e.g. gi: 167582254 from *Burkholderia thailandensis*. Given that integrated phages respond to stress by initiating the lytic cycle [1], it is likely that in these cases the phage-encoded SRAP acts as a DNA damage sensor and switch for the phage lysis pathway.

The SRAP superfamily is widespread in eukaryotes (prototyped by mammalian C3Orf37) and most likely goes back to the last eukaryotic common ancestor (Additional file 1). Thus, it could function as an important regulator of DNA repair and perhaps adaptive DNA mutagenesis even in eukaryotes. However, expression of the mammalian C3Orf37 in embryonic stem cells [21] suggests that at least in this context it is unlikely to activate a mutagenic response. Given the fact that it binds oxidized methylcytosine species [21], it is quite likely that it is part of the DNA repair process required for DNA demethylation. Several studies have shown that base excision repair proteins preferentially interact with oxidized methyl cytosine species (i.e. hmC, fC and caC) generated by the TET enzymes as opposed to unmodified 5mC [21,22]. In particular, the DNA glycosylase Tdg has been shown to be a potential player in the fC and caC excision process that leads to active DNA [24,25]. In light of this we suggest that eukaryotic representatives of the SRAP superfamily, such as C3Orf37, might play a role in recognizing oxidized mC species to recruit DNA repair enzymes that replace DNA containing these bases with unmodified bases. Thus, it could be a potential autoproteolytic control point that acts between the generation of oxidized mC species by the TET enzymes and the DNA repair enzymes that act on DNA with such modifications.

We hope that the findings presented here spur further investigation of these interesting protein domains both in bacterial and eukaryotic DNA repair and processes related to SOS and DNA demethylation that are downstream of the TET enzymes.

## Methods

Iterative sequence profile and HMM searches were respectively were performed using the PSI-BLAST [26] and HMMSEARCH program from the HMMER3 package [27] run against the non-redundant (NR; May10, 2013) protein database of National Center for Biotechnology Information (NCBI). Searches with the HMMSEARCH program were run with the listed parameters different from default (-E 12.5; --domE 12.5; --cpu 20; --incE .01). Iterative HMM searches with JACKHMMER were performed using the web utility (http://hmmer.janelia.org/search/jackhmmer). Multiple sequence alignments were built using MUSCLE [28] followed by manual adjustments on the basis of profile-profile and structural alignments. Similarity-based clustering for both classification and culling of nearly identical sequences was performed using the BLASTCLUST program (http://ftp.ncbi.nih.gov/blast/documents/blastclust.html). The HHpred program was used for profile-profile comparisons. Secondary structures were predicted using the JPred program [29]. For previously known domains the Pfam database was used as a guide, though the profiles were augmented by addition of newly detected divergent members that were not detected by the original Pfam models. Structural visualization and manipulations were performed using the PyMol program (http://www.pymol.org).

For each SRAP or ImuB-C gene the gene neighborhood was determined using either the PTT file (downloadable from the NCBI ftp site) or the Genbank file in the case of whole genome shot gun sequences. The neighbors of a given query gene were extracted with a preliminary cutoff of 5 genes on either side of the query. The protein sequences of all neighbors were clustered using the BLASTCLUST program (http://ftp.ncbi.nih.gov/blast/documents/blastclust.html) to identify related sequences in gene neighborhoods. Each cluster of homologous proteins was then assigned an annotation based on the domain architecture or conserved shared domain. This allowed an initial annotation of gene neigborhoods and their grouping based on conservation of neighborhood associations. This was further refined by ensuring that genes are unidirectional on the same strand of DNA and shared a putative common promoter to be counted as a single operon. If they were on opposite strands they were examined for potential bidirectional promoter sharing patterns.

In house Perl scripts were used to automate this analysis of genome context.

## Reviewers’ comments

### Reviewer 1: Robson de Souza (University of San Paulo, Brazil)

The work by Aravind et. al. describes two novel protein domains and demonstrates that these domains are often associated with SOS response genes and/or other DNA repair proteins. The authors show that YoqW-like genes are often in the vicinity of UmuCD or ImuAB-dnaE2 operons and that the ImuB-C domain is either fused to the C-terminal end of a family Y DNA polymerase or associated with other DNA polymerases in conserved gene neighborhoods. Based on the analysis of conserved residues of the YoqW domain and on its pattern of association with other SOS genes, the authors arrive to the interesting proposal that this domain may be a new SOS-related autoproteolytic thiol peptidase that might play a regulatory role analogous to LexA. The authors take note of a recent observation that eukaryotic homologs of YoqW are involved in recognition of oxidized derivatives of 5-methyl cytosine in DNA and suggest a HhH motif inside YoqW could responsible for such recognition. The molecular function of ImuB-C is less clear, since it does not seem to have a enzyme-like pattern of conserved residues, but the authors suggest, based on genomic context, that it might be a DNA binding domain and play a role in the recognition of DNA damage.

General considerations

This work presents solid evidence for the connection between the YoqW and ImuB-C domains and the SOS network and DNA repair. It makes intelligent use of available data on structures and other experimental results to infer the molecular function of YoqW, thus leading to a very interesting and extended analogy to LexA. Addition of these domains to the known repertoire of SOS-related protein domains is of great interest to drive further experimental studies on this system. In conclusion, I believe this work is fit for publication but I have some minor corrections and suggestions that I suggest the authors to consider (see below).

#### *Authors’ response*

We thank the referee for the positive evaluation of this work.

Minor issues

I ask the authors and the managers of Biology Direct submission system to investigate what happened to the bibliographic references in this submission. The PDF I downloaded from BD’s website contained only reference manager codes and no references. It forced me to search each reference I wanted to look at using last author name and year, a time consuming procedure I would not like to repeat.

#### *Authors’ response*

We believe there was some problem with the uploaded manuscript (as also noted by referee 2). We have now reformatted the revised manuscript and it should set this issue right.

Considering the importance of the ImuAB-dnaE2 operon for this work, I believe it is fair to add at least one citation of the paper that first characterized these genes in Caulobacter and showed their association with DNA repair and mutagenesis (Galhardo et al. (2005) An SOS-regulated operon involved in damage-inducible mutagenesis in Caulobacter crescentus. NAR, 33(8):2603-14, PMID: 15886391).

#### *Authors’ response*

We agree this is an important paper and have cited it in the revised manuscript in all the relevant contexts.

The paragraph that starts “Beyond the induction of SOS by various genotoxic stresses, it also appears to play a role” might read more clearly if rewritten like “Beyond the induction of SOS DNA-repair response by various genotoxic stresses, the SOS gene regulatory and protein interaction network also appears to play a role”.

#### *Authors’ response*

We have now modified that sentence so that it reads better.

Although I have complied with it so far, I must voice against referring to the new thiol peptidase domain by the Bacillus gene name YoqW. Gene names that start with “y” are traditionally used in genome annotation to name computationally predicted genes not associated with any phenotype or as synonyms for other gene names and are based on the chromosomal map location of the gene (PMID: 9729612). Such gene names will, therefore refer to entirely different genes in different organisms, even if closely related. I would suggest renaming DUF159 to something like SOSAE for “SOS-associated enzyme” and let the standard peptidase nomenclature wait for experimental validation.

#### *Authors’ response*

We agree this is a good suggestion. Using DUF159 or YoqW do not convey the clear functional implications of these proteins and are similarly opaque in this regard. Hence we have renamed the domain as SRAP for SOS response associated peptidase.

In the sentence “small domain globular domain of about 80 residues” remove the first domain”.

#### *Authors’ response*

We have corrected this in the revised manuscript.

With respect to the supplement, it would be of great value if the table of genomes contexts was sorted in some meaningful order, like by taxonomy, instead of an apparently random order. Also, redundancies in these tables should be removed.

#### *Authors’ response*

We have now reorganized the supplement, which hopefully helps better navigation.

In figure 2B, the authors might consider using a different color for the predicted DNA-binding HhH motif so that it is more easily identifiable in PDB:1ZN6.

#### *Authors’ response*

We have done as suggested in the revised version.

### Reviewer 2: Rob Finn (Howard Hughes Medical Institute, Janelia Farm Research Campus, USA)

The paper presented by Aravind et al describes the identification of two novel protein domain superfamilies that are part of the bacterial SOS response system. The authors use there typical broad range on analyses, including thorough protein sequence analysis, structural analysis and genomic context to provide insights into the potential function of otherwise uncharacterized protein families. The discovery of these families arose from looking at conserved proteins in operons contains sequences known to be involved in the bacterial SOS response. The first protein family described is YoqW or DUF159, and is found in bacterial and eukaryotes. Based on sequence conservation and known structures the authors present a compelling case that this is an autoproteolytic thiol peptidase.

#### *Authors’ response*

We appreciate the positive evaluation of this work.

I did find the description of the conserved glutamate a little tricky to follow, as there are two prominent glutamates in the sequence logo.

#### *Authors’ response*

Indeed there are two prominent glutamates. As described in the legend, the one which is part of the catalytic triad (inferred from the structures) is specifically marked with an asterisk above it. This one is nearly absolutely conserved, whereas the other one is less conserved as can be seen in the logo.

The second protein family discovered was ImuB-C, found after the Y DNA polymerase in ImuB and in isolation, reinforcing the fact it is a distinct functional unit. The authors suggest that this domain may function as part of a DNA repair system, based on the different operons that it is found in. The authors conjecture that the domain may be a DNA binding domain, given its size and lack of obvious conserved catalytic residues. I can find no evidence to disagree with this statement, but I wonder if there could not be alternative functions for this domain, such as a protein interaction module?

#### *Authors’ response*

*In principle, it is possible that the ImuB-C is involved in protein-protein interaction. However, several subtle domain architectural features weigh in favor of a DNA-binding function. First, it is found in association with a wide range of mechanistically distinct DNA repair systems (Network in Figure*2*) but rarely if ever in any non-DNA repair related contexts. Second, it is primarily prokaryotic in its phyletic spread, and there is no evidence for the use of a common protein-interaction signal coordinating across different DNA repair systems in prokaryotes (unlike eukaryotes where phosphorylation, and ubiquitin/ubiquitin-like modifiers and histone modifications are observed). Third, its position in the ImuB-C proteins is comparable to the DNA binding RAGNYA fold domain found in other family Y DNA polymerases.*

The authors conclude by describing how these protein families may function in context of the mutagenic arms of the SOS and how the homologs in eukaryotes may function. Overall, I can find no major fault with the paper from a scientific standpoint.

However, the uploaded file appeared to lack a formatted set of references, and without it I cannot judge whether all citations are correct.

#### *Authors’ response*

As mentioned in the response to reviewer 1 we have remedied this in the revised version.

There are several grammatical errors within the paper. For example, ‘small domain globular domain of about 80 amino acids’. I think a thorough read through of the manuscript will catch them. The spelling of authors should also be checked (uploaded MS may be wrong).

#### *Authors’ response*

We have corrected this in the revised manuscript.

There are also a few sentences where the scientific meaning is vague. If something is ‘strongly co-occurs’ what does that mean? Please support with numbers. Also, how was YoqW ‘transferred’ to eukaryotes? The authors should provide indicate where the HMMER search parameters were default.

#### *Authors’ response*

The first issue has been supported with numerical data in the revised manuscript. Regarding the transfer of YoqW to the eukaryotes: The superfamily is found across all major bacterial lineages suggesting that it was likely to have been present in the bacterial common ancestor. However, it is present only in less than 10% of the archaeal genomes and that too only in the euryarchaeal lineage. In eukaryotes it is present in most major eukaryotic lineages including the basal eukaryote Trichomonas. This phyletic pattern is most consistent with it being transferred from bacteria to eukaryotes, perhaps from early bacterial symbionts. The HMMER search parameters have been included in the revised version.

They should also provide the version (or date) of the NR sequence database that was used.

#### *Authors’ response*

This has been done.

Is the TASS package publicly available? If it is not, I am not sure why it mentioned.

#### *Authors’ response*

It has been removed in the revised manuscript.

### Reviewer 3: Gaspar Jekely (Max Planck Institute for developmental biology, Germany)

In this paper Aravind and colleagues describe two novel components of the bacterial SOS response, the YoqW and the ImuB-C domains, identified by gene-neighborhood analysis. Sequence conservation analyses and analyses of available structures suggest that the YoqW proteins have proteolytic activity and undergo autoproteolysis. The YoqW domain may thus represent a novel type of thiol protease. This is a fascinating insight, based on sequence and structure analysis alone, and illustrates how careful sequence searches and information from structural genomics can suggest functions for uncharacterized proteins. The function of the second identified domain (ImuB-C domain) is less clear, but the authors suggest, based on genomic context analysis, that it may be involved in binding damaged DNA structures and help recruit downstream components. I find this a very nice paper, and recommend publication without hesitation.

#### *Authors’ response*

We thank the referee for the positive evaluation of this work.

I only have one comment to the authors, regarding the software packages used. In the Methods section they mention an in-house collection of perl scripts (TASS package) used for the genome context analyses. Since the most important findings of this paper, the identification of the YoqW and ImuB-C domains, was based on genome context analyses, one is left wondering about the easy reproducibility of the finding, given that this is an in-house package. I noticed that in several other recent papers the authors used this package. Given that this collection of scripts was key for the most important finding of this (and also other) papers, I suggest that the authors provide this package in the supplementary information. Sharing software used to generate the data should be a trivial component of (open access!) publishing.

#### *Authors’ response*

We agree reproducibility is important; hence, provide in the supplementary information a complete set of genome contexts used in the inferences made here. We have also added a more detailed description of the procedure on the methods that can be reproduced by anyone with access to the NCBI databases and tools. Regarding the in house perl scripts the main issue is that they are not in state for general distribution because: First, they have not been documented sufficiently for this purpose. Second, they contain numerous dependencies that make them difficult to adapt across systems. Importantly, they need to be tweaked often to catch up with the ever-diverging formats within Genbank files. We also simply lack the resources to support their usability for other users. However, if a reader might still be interested they could email us for the same.

## Competing interests

The authors declare that they have no competing interests.

## Authors’ contributions

LA, SA and LMI collected data; LA, SA and LMI analyzed the data; SA and LMI prepared the figures; LA wrote the manuscript that was read and approved by all authors.

## Supplementary Material

Additional file 1 **Provides access to: 1) comprehensive list of Genbank identifiers, architectures and operons of modules uncovered in this study.** 2) A comprehensive set of alignments of domains reported here in text format. Click here for file
