# Supplementary material for: Novel autoproteolytic and DNA-damage sensing components in the bacterial SOS response and oxidized methylcytosine-induced eukaryotic DNA demethylation systems
Source: Biol Direct. 2013 Aug 15;8:20. doi: 10.1186/1745-6150-8-20 (PMC3765255; doi:10.1186/1745-6150-8-20)
Supplement: Additional file 1 — Provides access to: 1) comprehensive list of Genbank identifiers, architectures and operons of modules uncovered in this study. 2) A comprehensive set of alignments of domains reported here in text format. [file 1745-6150-8-20-S1.HTML]

Supplementary material for the manuscript "Novel autoproteolytic and DNA-damage sensing components in the bacterial SOS response and oxidized methylcytosine-induced eukaryotic DNA demethylation systems"
 type="text/css">

  
**Supplementary Material  
Novel autoproteolytic and DNA-damage sensing components in the bacterial SOS response and oxidized methylcytosine-induced eukaryotic DNA demethylation systems**  
  
 **L. Aravind**\*, Swadha Anand, and Lakshminarayan M. Iyer 
  
\* *Address for correspondence: L. Aravind (aravind@mail.nih.gov)*  
  
 *National Center for Biotechnology Information, National Library of Medicine, National Institutes of Health, Bethesda, MD 20894, USA*
  

---

|  |
| --- |
|  |
| **Abstract**The bacterial SOS response is an elaborate program for DNA repair, cell cycle regulation and adaptive mutagenesis under stress conditions. Using sensitive sequence and structure analysis, combined with contextual information derived from comparative genomics and domain architectures, we identify two novel domain superfamilies in the SOS response system. We present evidence that one of these, the SOS response associated peptidase (SRAP; Pfam: DUF159) is a novel thiol autopeptidase. Given the involvement of other autopeptidases, such as LexA and UmuD, in the SOS response, this finding suggests that multiple structurally unrelated peptidases have been recruited to this process. The second of these, the ImuB-C superfamily, is linked to the Y-family DNA polymerase-related domain in ImuB, and also occurs as a standalone protein. We present evidence using gene neighborhood analysis that both these domains function with different mutagenic polymerases in bacteria, such as Pol IV (DinB), Pol V (UmuCD) and ImuA-ImuB-DnaE2 and also other repair systems, which either deploy Ku and an ATP-dependent ligase or a SplB-like radical SAM photolyase. We suggest that the SRAP superfamily domain functions as a DNA-associated autoproteolytic switch that recruits diverse repair enzymes upon DNA damage, whereas the ImuB-C domain performs a similar function albeit in a non-catalytic fashion. We propose that C3Orf37, the eukaryotic member of the SRAP superfamily, which has been recently shown to specifically bind DNA with 5-hydroxymethylcytosine, 5-formylcytosine and 5-carboxycytosine, is a sensor for these oxidized bases generated by the TET enzymes from methylcytosine. Hence, its autoproteolytic activity might help it act as a switch that recruits DNA repair enzymes to remove these oxidized methylcytosine species as part of the DNA demethylation pathway downstream of the TET enzymes. |

|  |  |
| --- | --- |
|  | ---  **Contents**- **Species abbreviations in Figure 2** - **Phyletic distribution, domain architectures, gene neighborhoods and multiple sequence alignments of various systems described in this study**   1. SRAP domain      - Phyletic distribution and gene neighborhoods      - Multiple sequence alignment of the SRAP domain- ImuB-C domain        - Phyletic distribution and gene neighborhoods        - Multiple sequence alignment of the ImuB-C domain- Zinc Ribbons          - Multiple sequence alignment of the Zinc ribbon ZnR1          - Multiple sequence alignment of the Zinc ribbon ZnR2- **Species abbreviations for Figure 2**     Acac :*Anaerostipes caccae*; Acel : *Acetivibrio cellulolyticus*; Atum : *Agrobacterium tumefaciens*;Bgra: *Burkholderia graminis*; Bjap : *Bradyrhizobium japonicum*;       Bmar : *Blastopirellula marina*; Bmel: *Brucella melitensis*; Bpro : *Butyrivibrio proteoclasticus*; Caer : *Collinsella aerofaciens*; Caus : *Caloramator australicus*;      Cbol : *Clostridium bolteae*; Chyl : *Clostridium hylemonae*; Clep : *Clostridium leptum*; Cobs : *Caldicellulosiruptor obsidiansis*; Csp. : *Clostridium sp.*;      Dace : *Denitrovibrio acetiphilus*; Daci : *Desulfosporosinus acidiphilus*; Ddes : *Deinococcus deserti*; Ebac : *Erysipelotrichaceae bacterium*;       Elim : *Eubacterium limosum*; Esp. : *Eggerthella sp.*; Gpam : *Gordonibacter pamelaeae*; Gsul : *Geobacter sulfurreducens*; Lbac : *Lachnospiraceae bacterium*;      Mlot : *Mesorhizobium loti*; Msac : *Mariniradius saccharolyticus*; Mtub : *Mycobacterium tuberculosis*; Oval : *Oscillibacter valericigenes*;       PKSU : *planctomycete KSU-1*; Pcap : *Pseudoflavonifractor capillosus*; Pfer : *Pelosinus fermentans*; Ppar : *Pedosphaera parvula*; Ppro : *Pelobacter propionicus*;       Psyr : *Pseudomonas syringae*; Rbro : *Ruminococcus bromii*; Rinu : *Roseburia inulinivorans*; Shel : *Slackia heliotrinireducens*; Smel : *Sinorhizobium meliloti*;       Ssp. : *Sphingobacterium sp.*; Ssui : *Streptococcus suis*; Tbis : *Thermobispora bispora*.- **Phyletic distribution, domain architectures, gene neighborhoods of SRAP proteins**        ```       # UmuC-associated       406976067    <-SRAP*||UmuD->                                                            ACD_23C00337G0002               213   bacteria                                       uncultured bacterium                                                         hypothetical protein ACD_23C00337G0002 [uncultured bacterium].                                                                 <-406976066_?<-406976067_SRAP*||406976068_UmuD->406976069_?->406976070_?->       390947349    <-SRAP*<-UmuC<-UmuD                                                        -                               273   bacteria>bacteroidetes                         Alistipes finegoldii DSM 17242                                               hypothetical protein Alfi_2132 [Alistipes finegoldii DSM 17242].                                                               390947346_?->390947347_?->390947348_?-><-390947349_SRAP*<-390947350_UmuC<-390947351_UmuD||390947352_?->       334366525    <-SRAP*<-UmuC<-UmuD                                                        HMPREF9720_0255                 273   bacteria>bacteroidetes                         Alistipes sp. HGB5                                                           conserved hypothetical protein [Alistipes sp. HGB5].                                                                           334366520_?->334366516_?->334366518_?-><-334366525_SRAP*<-334366526_UmuC<-334366522_UmuD||334366519_?->       336413318    UmuD->UmuC->SRAP*->                                                        HMPREF1017_00778                232   bacteria>bacteroidetes                         Bacteroides ovatus 3_8_47FAA                                                 hypothetical protein HMPREF1017_00778 [Bacteroides ovatus 3_8_47FAA].                                                          <-336413315_?||336413316_UmuD->336413317_UmuC->336413318_SRAP*-><-336413319_?<-336413320_?<-336413321_?       301310299    UmuD->UmuC->SRAP*->                                                        HMPREF9008_00649                235   bacteria>bacteroidetes                         Bacteroides sp. 20_3                                                         conserved hypothetical protein [Bacteroides sp. 20_3].                                                                         <-301310296_?||301310297_UmuD->301310298_UmuC->301310299_SRAP*->301310300_?-><-301310301_?<-301310302_?       408671484    SRAP*-><-UmuD||DinB->DnaE2->                                               Emtol_0205                      242   bacteria>bacteroidetes                         Emticicia oligotrophica DSM 17448                                            protein of unknown function DUF159 [Emticicia oligotrophica DSM 17448].                                                        408671481_?->408671482_?->408671483_?->408671484_SRAP*-><-408671485_UmuD||408671486_DinB->408671487_DnaE2->       347537456    UmuD->UmuC->SRAP*->                                                        -                               212   bacteria>bacteroidetes                         Flavobacterium branchiophilum FL-15                                          hypothetical protein FBFL15_2652 [Flavobacterium branchiophilum FL-15].                                                        <-347537453_?||347537454_UmuD->347537455_UmuC->347537456_SRAP*-><-347537457_?<-347537458_?<-347537459_?       383449398    <-SRAP*<-UmuC<-UmuD                                                        KQS_00245                       198   bacteria>bacteroidetes                         Flavobacterium indicum GPTSA100-9                                            hypothetical protein KQS_00245 [Flavobacterium indicum GPTSA100-9].                                                            383449395_?->383449396_?->383449397_?-><-383449398_SRAP*<-383449399_UmuC<-383449400_UmuD<-383449401_?       471946564    UmuC-><-?||SRAP*->                                                         D778_02367                      245   bacteria>bacteroidetes                         Formosa sp. AK20                                                             hypothetical protein D778_02367 [Formosa sp. AK20].                                                                            471946562_UmuC-><-471946563_?||471946564_SRAP*-><-471946565_?<-471946566_?<-471946567_?       384099083    <-UmuD||DinB->DnaE2->SRAP*->                                               W5A_10462                       245   bacteria>bacteroidetes                         Imtechella halotolerans K1                                                   hypothetical protein W5A_10462 [Imtechella halotolerans K1].                                                                   <-384099080_UmuD||384099081_DinB->384099082_DnaE2->384099083_SRAP*-><-384099084_?||384099085_?-><-384099086_?       375148601    UmuD->UmuC->?->SRAP*->                                                     -                               265   bacteria>bacteroidetes                         Niastella koreensis GR20-10                                                  hypothetical protein [Niastella koreensis GR20-10].                                                                            375148598_UmuD->375148599_UmuC->375148600_?->375148601_SRAP*->375148602_?->375148603_?->375148604_?->       409097154    UmuD->UmuC->SRAP*->                                                        PagrP_010100001549              226   bacteria>bacteroidetes                         Pedobacter agri PB92                                                         YoqW protein [Pedobacter agri PB92].                                                                                           <-409097151_?||409097152_UmuD->409097153_UmuC->409097154_SRAP*->409097155_?->409097156_?->409097157_?->       149278481    UmuD->UmuC->SRAP*->?->Sigma->                                              PBAL39_20159                    203   bacteria>bacteroidetes                         Pedobacter sp. BAL39                                                         hypothetical protein PBAL39_20159 [Pedobacter sp. BAL39].                                                                      149278478_?->149278479_UmuD->149278480_UmuC->149278481_SRAP*->149278482_?->149278483_Sigma->149278484_?->       325955462    UmuC->UmuD-><-SRAP*<-MutS||UDG->                                           -                               193   bacteria>bacteroidetes                         Weeksella virosa DSM 16922                                                   hypothetical protein [Weeksella virosa DSM 16922].                                                                             <-325955459_?||325955460_UmuC->325955461_UmuD-><-325955462_SRAP*<-325955463_MutS||325955464_UDG-><-325955465_?       325168972    <-Resolvase<-SRAP*||UmuD->UmuC->                                           ACMV_P2_00410                   224   bacteria>proteobacteria>alphaproteobacteria    Acidiphilium multivorum AIU301                                               hypothetical protein ACMV_P2_00410 [Acidiphilium multivorum AIU301].                                                           <-325168969_?<-325168970_?<-325168971_Resolvase<-325168972_SRAP*||325168973_UmuD->325168974_UmuC-><-325168975_?       296284324    UmuD->?->SRAP*->                                                           CbatJ_010100011896              195   bacteria>proteobacteria>alphaproteobacteria    Citromicrobium bathyomarinum JL354                                           hypothetical protein CbatJ_11896 [Citromicrobium bathyomarinum JL354].                                                         <-296284321_?||296284322_UmuD->296284323_?->296284324_SRAP*-><-296284325_?<-296284326_?||296284327_?->       338992184    <-UmuC<-UmuD||SRAP*->SRAP->                                                APM_3146                        227   bacteria>proteobacteria>alphaproteobacteria    Acidiphilium sp. PM                                                          hypothetical protein APM_3146 [Acidiphilium sp. PM].                                                                           <-338992181_?<-338992182_UmuC<-338992183_UmuD||338992184_SRAP*->338992185_SRAP->       338992218    RecD->?->?-><-SRAP*||UmuD->UmuC->                                          APM_3554                        247   bacteria>proteobacteria>alphaproteobacteria    Acidiphilium sp. PM                                                          hypothetical protein APM_3554 [Acidiphilium sp. PM].                                                                           338992214_RecD->338992215_?->338992216_?-><-338992218_SRAP*||338992217_UmuD->338992219_UmuC->338992220_?->       395494178    <-SRAP*||UmuD->UmuC->                                                      SPAM26_010100020223             189   bacteria>proteobacteria>alphaproteobacteria    Sphingomonas sp. PAMC 26617                                                  hypothetical protein SPAM26_20223 [Sphingomonas sp. PAMC 26617].                                                               395494175_?->395494176_?-><-395494177_?<-395494178_SRAP*||395494179_UmuD->395494180_UmuC-><-395494181_?       259415883    UmuD-><-SRAP*                                                              SCH4B_1729                      227   bacteria>proteobacteria>alphaproteobacteria    Silicibacter sp. TrichCH4B                                                   conserved hypothetical protein [Silicibacter sp. TrichCH4B].                                                                   <-259416428_?<-259416211_?||259416048_UmuD-><-259415883_SRAP*<-259416638_?<-259416997_?||259416513_?->       148244042    <-UmuC<-UmuD||SRAP*-><-?||?-><-RecD                                        Acry_3538                       236   bacteria>proteobacteria>alphaproteobacteria    Acidiphilium cryptum JF-5                                                    hypothetical protein Acry_3538 [Acidiphilium cryptum JF-5].                                                                    148244039_?-><-148244040_UmuC<-148244041_UmuD||148244042_SRAP*-><-148244043_?||148244044_?-><-148244045_RecD       402822464    UmuD->UmuC->SRAP*->                                                        LH128_06512                     208   bacteria>proteobacteria>alphaproteobacteria    Sphingomonas sp. LH128                                                       hypothetical protein LH128_06512 [Sphingomonas sp. LH128].                                                                     402822461_?->402822462_UmuD->402822463_UmuC->402822464_SRAP*->402822465_?-><-402822466_?<-402822467_?       404254820    <-SRAP*||UmuD->UmuC->UmuC->                                                SPAM266_010100016421            210   bacteria>proteobacteria>alphaproteobacteria    Sphingomonas sp. PAMC 26621                                                  hypothetical protein SPAM266_16421 [Sphingomonas sp. PAMC 26621].                                                              404254817_?->404254818_?-><-404254819_?<-404254820_SRAP*||404254821_UmuD->404254822_UmuC->404254823_UmuC->       197105685    UmuD->SRAP*->                                                              -                               183   bacteria>proteobacteria>alphaproteobacteria    Phenylobacterium zucineum HLK1                                               hypothetical protein PHZ_c2223 [Phenylobacterium zucineum HLK1].                                                               <-197105682_?<-197105683_?||197105684_UmuD->197105685_SRAP*-><-197105686_?<-197105687_?<-197105688_?       359797831    <-SRAP*<-?||?-><-UmuD                                                      KYC_12843                       220   bacteria>proteobacteria>betaproteobacteria     Achromobacter arsenitoxydans SY8                                             hypothetical protein KYC_12843 [Achromobacter arsenitoxydans SY8].                                                             <-359797828_?<-359797829_?<-359797830_?<-359797831_SRAP*<-359797832_?||359797833_?-><-359797834_UmuD       351732355    <-SSB<-SRAP*||UmuD->UmuC->                                                 AradN_010100021372              231   bacteria>proteobacteria>betaproteobacteria     Acidovorax radicis N35                                                       hypothetical protein AradN_21372 [Acidovorax radicis N35].                                                                     <-351732352_?||351732353_?-><-351732354_SSB<-351732355_SRAP*||351732356_UmuD->351732357_UmuC->351732358_?->       395010495    <-UmuC<-UmuD||SRAP*->                                                      PMI14_06800                     231   bacteria>proteobacteria>betaproteobacteria     Acidovorax sp. CF316                                                         hypothetical protein PMI14_06800 [Acidovorax sp. CF316].                                                                       <-395010492_?<-395010493_UmuC<-395010494_UmuD||395010495_SRAP*->395010496_?->395010497_?->       319762556    <-SRAP*||UmuD->UmuC->                                                      -                               223   bacteria>proteobacteria>betaproteobacteria     Alicycliphilus denitrificans BC                                              hypothetical protein Alide_1859 [Alicycliphilus denitrificans BC].                                                             319762553_?->319762554_?->319762555_?-><-319762556_SRAP*||319762557_UmuD->319762558_UmuC-><-319762559_?       470169817    <-SRAP*<-?||UmuD->UmuC->                                                   -                               216   bacteria>proteobacteria>betaproteobacteria     Azoarcus sp. KH32C                                                           hypothetical protein AZKH_1405 [Azoarcus sp. KH32C].                                                                           <-470169814_?<-470169815_?<-470169816_?<-470169817_SRAP*<-470169818_?||470169819_UmuD->470169820_UmuC->       302879605    <-UmuC<-UmuD<-SRAP*<-RecD                                                  Galf_2403                       208   bacteria>proteobacteria>betaproteobacteria     Gallionella capsiferriformans ES-2                                           hypothetical protein Galf_2403 [Gallionella capsiferriformans ES-2].                                                           <-302879602_?<-302879603_UmuC<-302879604_UmuD<-302879605_SRAP*<-302879606_RecD<-302879607_?<-302879608_?       254003148    <-UmuC<-UmuD<-SRAP*                                                        Msip34_2863                     216   bacteria>proteobacteria>betaproteobacteria     Methylovorus glucosetrophus SIP3-4                                           hypothetical protein Msip34_2863 [Methylovorus glucosetrophus SIP3-4].                                                         254003145_?-><-254003146_UmuC<-254003147_UmuD<-254003148_SRAP*<-254003149_?<-254003150_?<-254003151_?       253999313    SRAP*-><-?||UmuD->UmuC->                                                   -                               219   bacteria>proteobacteria>betaproteobacteria     Methylovorus glucosetrophus SIP3-4                                           hypothetical protein Msip34_1604 [Methylovorus glucosetrophus SIP3-4].                                                         <-253999310_?<-253999311_?||253999312_?->253999313_SRAP*-><-253999314_?||253999315_UmuD->253999316_UmuC->       114326595    <-SRAP*||UmuD->UmuC->                                                      Neut_2573                       210   bacteria>proteobacteria>betaproteobacteria     Nitrosomonas eutropha C91                                                    hypothetical protein Neut_2573 [Nitrosomonas eutropha C91].                                                                    <-114326592_?||114326593_?-><-114326594_?<-114326595_SRAP*||114326596_UmuD->114326597_UmuC-><-114326598_?       347539475    <-UmuC<-UmuD||SRAP*->                                                      -                               241   bacteria>proteobacteria>betaproteobacteria     Pseudogulbenkiania sp. NH8B                                                  hypothetical protein NH8B_1669 [Pseudogulbenkiania sp. NH8B].                                                                  <-347539472_?<-347539473_UmuC<-347539474_UmuD||347539475_SRAP*-><-347539476_?||347539477_?->347539478_?->       89885830     <-UmuC<-UmuD||?->SRAP*->                                                   Rfer_4344                       215   bacteria>proteobacteria>betaproteobacteria     Rhodoferax ferrireducens T118                                                hypothetical protein Rfer_4344 [Rhodoferax ferrireducens T118].                                                                <-89885827_UmuC<-89885828_UmuD||89885829_?->89885830_SRAP*->89885831_?->89885832_?->89885833_?->       394990642    UmuD->UmuC->SRAP*->                                                        SCD_03070                       221   bacteria>proteobacteria>betaproteobacteria     Sulfuricella denitrificans skB26                                             hypothetical protein SCD_03070 [Sulfuricella denitrificans skB26].                                                             394990639_?->394990640_UmuD->394990641_UmuC->394990642_SRAP*->394990643_?->394990644_?-><-394990645_?       479306720    <-SRAP*||UmuD->UmuC->                                                      C662_01285                      224   bacteria>proteobacteria>betaproteobacteria     Thauera sp. 28                                                               hypothetical protein C662_01285 [Thauera sp. 28].                                                                              479306717_?->479306718_?->479306719_?-><-479306720_SRAP*||479306721_UmuD->479306722_UmuC->479306723_?->       296137340    <-UmuC<-UmuD||SRAP*->?-><-transposase                                      Tint_2914                       223   bacteria>proteobacteria>betaproteobacteria     Thiomonas intermedia K12                                                     hypothetical protein Tint_2914 [Thiomonas intermedia K12].                                                                     <-296137337_?<-296137338_UmuC<-296137339_UmuD||296137340_SRAP*->296137341_?-><-296137342_transposase<-296137343_?       303246529    SRAP*->UmuD->UmuC->                                                        DesfrDRAFT_1283                 205   bacteria>proteobacteria>deltaproteobacteria    Desulfovibrio fructosovorans JJ                                              protein of unknown function DUF159 [Desulfovibrio fructosovorans JJ].                                                          303246526_?-><-303246527_?<-303246528_?||303246529_SRAP*->303246530_UmuD->303246531_UmuC->303246532_?->       374288499    <-SRAP*||UmuD->UmuC->                                                      -                               220   bacteria>proteobacteria>deltaproteobacteria    Bacteriovorax marinus SJ                                                     hypothetical protein BMS_1794 [Bacteriovorax marinus SJ].                                                                      374288496_?-><-374288497_?||374288498_?-><-374288499_SRAP*||374288500_UmuD->374288501_UmuC-><-374288502_?       344198925    <-UmuC<-UmuD<-1TM<-SRAP*||RecD->                                           -                               227   bacteria>proteobacteria>gammaproteobacteria    Acidithiobacillus ferrivorans SS3                                            hypothetical protein Acife_0727 [Acidithiobacillus ferrivorans SS3].                                                           <-344198922_UmuC<-344198923_UmuD<-344198924_1TM<-344198925_SRAP*||344198926_RecD-><-344198927_?||344198928_?->       218665360    SRAP*-><-?||UmuD->UmuC->                                                   -                               194   bacteria>proteobacteria>gammaproteobacteria    Acidithiobacillus ferrooxidans ATCC 23270                                    hypothetical protein AFE_1749 [Acidithiobacillus ferrooxidans ATCC 23270].                                                     <-218666597_?<-218666141_?<-218667956_?||218665360_SRAP*-><-218666217_?||218667986_UmuD->218665203_UmuC->       384086764    <-UmuC<-UmuD<-SRAP*||RecD->                                                AthiA1_010100014856             227   bacteria>proteobacteria>gammaproteobacteria    Acidithiobacillus thiooxidans ATCC 19377                                     hypothetical protein AthiA1_14856 [Acidithiobacillus thiooxidans ATCC 19377].                                                  384086761_?-><-384086762_UmuC<-384086763_UmuD<-384086764_SRAP*||384086765_RecD-><-384086766_?||384086767_?->       332853918    <-UmuD<-SRAP*                                                              HMPREF0021_02640                213   bacteria>proteobacteria>gammaproteobacteria    Acinetobacter baumannii 6013150                                              hypothetical protein HMPREF0021_02640 [Acinetobacter baumannii 6013150].                                                       <-332853915_?<-332853916_?<-332853917_UmuD<-332853918_SRAP*<-332853919_?<-332853920_?||332853921_?->       332852422    SRAP*->UmuD->UmuC->                                                        HMPREF0021_01766                211   bacteria>proteobacteria>gammaproteobacteria    Acinetobacter baumannii 6013150                                              hypothetical protein HMPREF0021_01766 [Acinetobacter baumannii 6013150].                                                       <-332852419_?<-332852420_?<-332852421_?||332852422_SRAP*->332852423_UmuD->332852424_UmuC->332852425_?->       213156268    <-UmuC<-UmuD<-SRAP*                                                        -                               211   bacteria>proteobacteria>gammaproteobacteria    Acinetobacter baumannii AB0057                                               hypothetical protein AB57_1307 [Acinetobacter baumannii AB0057].                                                               <-213156265_?<-213156266_UmuC<-213156267_UmuD<-213156268_SRAP*<-213156269_?<-213156270_?<-213156271_?       416150314    SRAP*->UmuD->UmuC->                                                        AB210_3385                      213   bacteria>proteobacteria>gammaproteobacteria    Acinetobacter baumannii AB210                                                hypothetical protein AB210_3385 [Acinetobacter baumannii AB210].                                                               <-416150311_?<-416150312_?<-416150313_?||416150314_SRAP*->416150315_UmuD->416150316_UmuC-><-416150317_?       458204837    <-UmuC<-UmuD<-SRAP*                                                        H021_RS04940                    211   bacteria>proteobacteria>gammaproteobacteria    Acinetobacter baumannii AB5711                                               hypothetical protein H021_RS04940 [Acinetobacter baumannii AB5711].                                                            <-458204834_?<-458204835_UmuC<-458204836_UmuD<-458204837_SRAP*||458204838_?->458204839_?->458204840_?->       417869193    <-UmuC<-UmuD<-SRAP*                                                        ABNIH1_03372                    211   bacteria>proteobacteria>gammaproteobacteria    Acinetobacter baumannii ABNIH1                                               hypothetical protein ABNIH1_03372 [Acinetobacter baumannii ABNIH1].                                                            <-417869190_?<-417869191_UmuC<-417869192_UmuD<-417869193_SRAP*||417869194_?->417869195_?->417869196_?->       458547492    SRAP*->UmuD->UmuC->                                                        B740_RS04910                    218   bacteria>proteobacteria>gammaproteobacteria    Acinetobacter baumannii AB_1536-8                                            hypothetical protein B740_RS04910 [Acinetobacter baumannii AB_1536-8].                                                         <-458547489_?||458547490_?->458547491_?->458547492_SRAP*->458547493_UmuD->458547494_UmuC->458547495_?->       457959062    SRAP*->UmuD->UmuC->                                                        B743_RS07495                    211   bacteria>proteobacteria>gammaproteobacteria    Acinetobacter baumannii AB_1594-8                                            hypothetical protein B743_RS07495 [Acinetobacter baumannii AB_1594-8].                                                         <-457959059_?<-457959060_?||457959061_?->457959062_SRAP*->457959063_UmuD->457959064_UmuC->457959065_?->       458423053    SRAP*->UmuD->UmuC->                                                        B749_RS16775                    214   bacteria>proteobacteria>gammaproteobacteria    Acinetobacter baumannii AB_2007-16-25-01-7                                   hypothetical protein B749_RS16775 [Acinetobacter baumannii AB_2007-16-25-01-7].                                                <-458423050_?||458423051_?-><-458423052_?||458423053_SRAP*->458423054_UmuD->458423055_UmuC-><-458423056_?       457881104    SRAP*->UmuD->UmuC->                                                        B759_RS04415                    222   bacteria>proteobacteria>gammaproteobacteria    Acinetobacter baumannii AB_2009-04-01-7                                      hypothetical protein B759_RS04415 [Acinetobacter baumannii AB_2009-04-01-7].                                                   <-457881101_?<-457881102_?||457881103_?->457881104_SRAP*->457881105_UmuD->457881106_UmuC->457881107_?->       458293899    <-UmuC<-UmuD<-SRAP*                                                        B780_RS07545                    170   bacteria>proteobacteria>gammaproteobacteria    Acinetobacter baumannii AB_TG27323                                           hypothetical protein B780_RS07545 [Acinetobacter baumannii AB_TG27323].                                                        <-458293896_?<-458293897_UmuC<-458293898_UmuD<-458293899_SRAP*||458293900_?->458293901_?->458293902_?->       458741835    <-UmuC<-UmuD<-SRAP*                                                        B785_RS01365                    214   bacteria>proteobacteria>gammaproteobacteria    Acinetobacter baumannii AB_TG27343                                           hypothetical protein B785_RS01365 [Acinetobacter baumannii AB_TG27343].                                                        <-458741832_?<-458741833_UmuC<-458741834_UmuD<-458741835_SRAP*||458741836_?-><-458741837_?<-458741838_?       457983682    <-UmuC<-UmuD<-SRAP*||?->SRAP->                                             B787_RS02025                    200   bacteria>proteobacteria>gammaproteobacteria    Acinetobacter baumannii AB_TG5064                                            hypothetical protein B787_RS02025 [Acinetobacter baumannii AB_TG5064].                                                         <-457983680_UmuC<-457983681_UmuD<-457983682_SRAP*||457983683_?->457983684_SRAP->       193077599    SRAP*->UmuC->                                                              A1S_2014                        260   bacteria>proteobacteria>gammaproteobacteria    Acinetobacter baumannii ATCC 17978                                           hypothetical protein A1S_2014 [Acinetobacter baumannii ATCC 17978].                                                            <-193077597_?<-126387942_?||193077598_?->193077599_SRAP*->193077600_UmuC-><-193077601_?<-193077602_?       424052997    SRAP*->UmuD->UmuC->                                                        -                               211   bacteria>proteobacteria>gammaproteobacteria    Acinetobacter baumannii Ab11111                                              hypothetical protein W9G_01686 [Acinetobacter baumannii Ab11111].                                                              424052994_?->424052995_?->424052996_?->424052997_SRAP*->424052998_UmuD->424052999_UmuC->424053000_?->       458727604    SRAP*->UmuD->UmuC->                                                        B312_RS14610                    209   bacteria>proteobacteria>gammaproteobacteria    Acinetobacter baumannii BZICU-2                                              hypothetical protein B312_RS14610 [Acinetobacter baumannii BZICU-2].                                                           458727601_?->458727602_?->458727603_?->458727604_SRAP*->458727605_UmuD->458727606_UmuC->458727607_?->       458724170    <-UmuC<-UmuD<-SRAP*                                                        B312_RS03935                    213   bacteria>proteobacteria>gammaproteobacteria    Acinetobacter baumannii BZICU-2                                              hypothetical protein B312_RS03935 [Acinetobacter baumannii BZICU-2].                                                           <-458724167_?<-458724168_UmuC<-458724169_UmuD<-458724170_SRAP*<-458724171_?<-458724172_?<-458724173_?       479918070    <-UmuC<-UmuD<-SRAP*                                                        F979_01702                      214   bacteria>proteobacteria>gammaproteobacteria    Acinetobacter baumannii NIPH 146                                             hypothetical protein F979_01702 [Acinetobacter baumannii NIPH 146].                                                            <-479918067_?<-479918068_UmuC<-479918069_UmuD<-479918070_SRAP*<-479918071_?<-479918072_?<-479918073_?       480104406    SRAP*->UmuD->UmuC->                                                        F920_02537                      211   bacteria>proteobacteria>gammaproteobacteria    Acinetobacter baumannii NIPH 335                                             hypothetical protein F920_02537 [Acinetobacter baumannii NIPH 335].                                                            480104403_?->480104404_?->480104405_?->480104406_SRAP*->480104407_UmuD->480104408_UmuC->480104409_?->       480124126    <-UmuC<-UmuD<-SRAP*                                                        F915_01480                      117   bacteria>proteobacteria>gammaproteobacteria    Acinetobacter baumannii NIPH 70                                              hypothetical protein F915_01480 [Acinetobacter baumannii NIPH 70].                                                             <-480124123_?<-480124124_UmuC<-480124125_UmuD<-480124126_SRAP*<-480124127_?<-480124128_?<-480124129_?       417549270    SRAP*->UmuD->UmuC->                                                        -                               211   bacteria>proteobacteria>gammaproteobacteria    Acinetobacter baumannii Naval-18                                             hypothetical protein ACINNAV18_3065 [Acinetobacter baumannii Naval-18].                                                        417549163_?->417549420_?->417550059_?->417549270_SRAP*->417549794_UmuD->417549667_UmuC->417549018_?->       417551425    <-UmuC<-UmuD<-SRAP*                                                        ACINNAV18_1324                  211   bacteria>proteobacteria>gammaproteobacteria    Acinetobacter baumannii Naval-18                                             hypothetical protein ACINNAV18_1324 [Acinetobacter baumannii Naval-18].                                                        <-417551418_?<-417551103_UmuC<-417551592_UmuD<-417551425_SRAP*<-417551345_?<-417551319_?<-417551274_?       421652132    SRAP*->UmuD->UmuC->                                                        ACIN5162_2239                   212   bacteria>proteobacteria>gammaproteobacteria    Acinetobacter baumannii OIFC0162                                             hypothetical protein ACIN5162_2239 [Acinetobacter baumannii OIFC0162].                                                         <-421652076_?||421652129_?->421652132_SRAP*->421652134_UmuD->421652136_UmuC->421652082_?->       445431225    SRAP*->UmuD->UmuC->                                                        ACIN5021_1054                   107   bacteria>proteobacteria>gammaproteobacteria    Acinetobacter baumannii OIFC021                                              hypothetical protein ACIN5021_1054 [Acinetobacter baumannii OIFC021].                                                          <-445431242_?<-445431221_?<-445431247_?||445431225_SRAP*->445431215_UmuD->445431249_UmuC->445431194_?->       421807455    SRAP*->UmuD->UmuC->                                                        ACIN5035_1130                   211   bacteria>proteobacteria>gammaproteobacteria    Acinetobacter baumannii OIFC035                                              hypothetical protein ACIN5035_1130 [Acinetobacter baumannii OIFC035].                                                          421807509_?->421807569_?->421807639_?->421807455_SRAP*->421807501_UmuD->421807541_UmuC->421807627_?->       445457857    SRAP*->UmuD->UmuC->                                                        ACIN5047_2035                   214   bacteria>proteobacteria>gammaproteobacteria    Acinetobacter baumannii OIFC047                                              hypothetical protein ACIN5047_2035 [Acinetobacter baumannii OIFC047].                                                          <-445457892_?||445457849_?->445457904_?->445457857_SRAP*->445457828_UmuD->445457876_UmuC->445457819_?->       421625278    SRAP*->UmuD->UmuC->                                                        ACIN5098_0948                   211   bacteria>proteobacteria>gammaproteobacteria    Acinetobacter baumannii OIFC098                                              hypothetical protein ACIN5098_0948 [Acinetobacter baumannii OIFC098].                                                          421625381_?-><-421625345_?<-421625373_?||421625278_SRAP*->421625323_UmuD->421625285_UmuC->421625298_?->       421671336    SRAP*->UmuD->UmuC->                                                        ACIN5099_1151                   177   bacteria>proteobacteria>gammaproteobacteria    Acinetobacter baumannii OIFC099                                              hypothetical protein ACIN5099_1151 [Acinetobacter baumannii OIFC099].                                                          <-421671334_?<-421671332_?||421671336_SRAP*->421671337_UmuD->421671333_UmuC->421671330_?->       421663288    SRAP*->UmuD->UmuC->                                                        ACIN5110_2502                   211   bacteria>proteobacteria>gammaproteobacteria    Acinetobacter baumannii OIFC110                                              hypothetical protein ACIN5110_2502 [Acinetobacter baumannii OIFC110].                                                          421663288_SRAP*->421663284_UmuD->421663287_UmuC->421663285_?->       424742978    <-UmuC<-UmuD<-SRAP*                                                        ACINWC141_1148                  170   bacteria>proteobacteria>gammaproteobacteria    Acinetobacter baumannii WC-141                                               hypothetical protein ACINWC141_1148 [Acinetobacter baumannii WC-141].                                                          <-424742982_?<-424742988_UmuC<-424742969_UmuD<-424742978_SRAP*<-424742954_?<-424742999_?<-424742968_?       425745088    SRAP*->UmuD->UmuC->                                                        ACINWC323_1325                  215   bacteria>proteobacteria>gammaproteobacteria    Acinetobacter baumannii WC-323                                               hypothetical protein ACINWC323_1325 [Acinetobacter baumannii WC-323].                                                          425745039_?-><-425745059_?||425745014_?->425745088_SRAP*->425745005_UmuD->425745008_UmuC-><-425745086_?       425745532    <-UmuC<-UmuD||?-><-SRAP*                                                   ACINWC323_1519                  210   bacteria>proteobacteria>gammaproteobacteria    Acinetobacter baumannii WC-323                                               hypothetical protein ACINWC323_1519 [Acinetobacter baumannii WC-323].                                                          <-425745419_UmuC<-425745483_UmuD||425745582_?-><-425745532_SRAP*||425745566_?->425745372_?->425745554_?->       425743349    SRAP*->UmuD->UmuC->                                                        ACINWC323_3616                  150   bacteria>proteobacteria>gammaproteobacteria    Acinetobacter baumannii WC-323                                               hypothetical protein ACINWC323_3616 [Acinetobacter baumannii WC-323].                                                          425743349_SRAP*->425743352_UmuD->425743353_UmuC->425743351_?->       421696682    <-UmuC<-UmuD<-SRAP*                                                        ACINWC692_2353                  211   bacteria>proteobacteria>gammaproteobacteria    Acinetobacter baumannii WC-692                                               hypothetical protein ACINWC692_2353 [Acinetobacter baumannii WC-692].                                                          421696640_?-><-421696651_UmuC<-421696575_UmuD<-421696682_SRAP*<-421696635_?<-421696714_?<-421696655_?       480066915    <-UmuC<-UmuD<-SRAP*                                                        F934_01124                      214   bacteria>proteobacteria>gammaproteobacteria    Acinetobacter beijerinckii ANC 3835                                          hypothetical protein F934_01124 [Acinetobacter beijerinckii ANC 3835].                                                         <-480066912_?<-480066913_UmuC<-480066914_UmuD<-480066915_SRAP*<-480066916_?<-480066917_?||480066918_?->       479976361    SRAP*->UmuD->UmuC->                                                        F963_03661                      214   bacteria>proteobacteria>gammaproteobacteria    Acinetobacter bereziniae NIPH 3                                              hypothetical protein F963_03661 [Acinetobacter bereziniae NIPH 3].                                                             <-479976358_?||479976359_?-><-479976360_?||479976361_SRAP*->479976362_UmuD->479976363_UmuC->479976364_?->       480055089    SRAP*-><-?||UmuD->UmuC->                                                   F937_03535                      206   bacteria>proteobacteria>gammaproteobacteria    Acinetobacter calcoaceticus ANC 3680                                         hypothetical protein F937_03535 [Acinetobacter calcoaceticus ANC 3680].                                                        <-480055086_?<-480055087_?<-480055088_?||480055089_SRAP*-><-480055090_?||480055091_UmuD->480055092_UmuC->       480060023    SRAP*->?->UmuD->UmuC->                                                     F936_02051                      206   bacteria>proteobacteria>gammaproteobacteria    Acinetobacter calcoaceticus DSM 30006 = CIP 81.8                             hypothetical protein F936_02051 [Acinetobacter calcoaceticus DSM 30006 = CIP 81.8].                                            480060020_?->480060021_?-><-480060022_?||480060023_SRAP*->480060024_?->480060025_UmuD->480060026_UmuC->       262279798    SRAP*-><-?||UmuD->UmuC->                                                   HMPREF0012_01751                210   bacteria>proteobacteria>gammaproteobacteria    Acinetobacter calcoaceticus RUH2202                                          conserved hypothetical protein [Acinetobacter calcoaceticus RUH2202].                                                          262279795_?->262279796_?->262279797_?->262279798_SRAP*-><-262279799_?||262279800_UmuD->262279801_UmuC->       479910719    <-UmuC<-UmuD<-SRAP*                                                        F981_01689                      214   bacteria>proteobacteria>gammaproteobacteria    Acinetobacter guillouiae CIP 63.46                                           hypothetical protein F981_01689 [Acinetobacter guillouiae CIP 63.46].                                                          479910716_?-><-479910717_UmuC<-479910718_UmuD<-479910719_SRAP*||479910720_?->479910721_?->479910722_?->       479972197    SRAP*->UmuD->UmuC->                                                        F964_03372                      214   bacteria>proteobacteria>gammaproteobacteria    Acinetobacter guillouiae NIPH 991                                            hypothetical protein F964_03372 [Acinetobacter guillouiae NIPH 991].                                                           <-479972194_?<-479972195_?||479972196_?->479972197_SRAP*->479972198_UmuD->479972199_UmuC->479972200_?->       480032464    SRAP*->UmuD->UmuC->                                                        F946_01954                      207   bacteria>proteobacteria>gammaproteobacteria    Acinetobacter johnsonii ANC 3681                                             hypothetical protein F946_01954 [Acinetobacter johnsonii ANC 3681].                                                            480032461_?->480032462_?-><-480032463_?||480032464_SRAP*->480032465_UmuD->480032466_UmuC->480032467_?->       479891237    <-UmuC<-UmuD<-SRAP*                                                        F986_00009                      171   bacteria>proteobacteria>gammaproteobacteria    Acinetobacter johnsonii CIP 64.6                                             hypothetical protein F986_00009 [Acinetobacter johnsonii CIP 64.6].                                                            <-479891234_?<-479891235_UmuC<-479891236_UmuD<-479891237_SRAP*<-479891238_?||479891239_?-><-479891240_?       479890290    SRAP*->UmuD->UmuC->                                                        F986_00864                      216   bacteria>proteobacteria>gammaproteobacteria    Acinetobacter johnsonii CIP 64.6                                             hypothetical protein F986_00864 [Acinetobacter johnsonii CIP 64.6].                                                            479890287_?->479890288_?-><-479890289_?||479890290_SRAP*->479890291_UmuD->479890292_UmuC->479890293_?->       262368259    SRAP*->UmuD->UmuC->                                                        HMPREF0016_00058                171   bacteria>proteobacteria>gammaproteobacteria    Acinetobacter johnsonii SH046                                                conserved hypothetical protein [Acinetobacter johnsonii SH046].                                                                262368256_?-><-262368257_?||262368258_?->262368259_SRAP*->262368260_UmuD->262368261_UmuC->262368262_?->       262371447    <-UmuC<-UmuD<-SRAP*                                                        HMPREF0016_03234                123   bacteria>proteobacteria>gammaproteobacteria    Acinetobacter johnsonii SH046                                                conserved hypothetical protein [Acinetobacter johnsonii SH046].                                                                <-262371444_?<-262371445_UmuC<-262371446_UmuD<-262371447_SRAP*       381196329    SRAP*->UmuD->UmuC->                                                        AlwoW_010100003555              216   bacteria>proteobacteria>gammaproteobacteria    Acinetobacter lwoffii WJ10621                                                hypothetical protein AlwoW_03555 [Acinetobacter lwoffii WJ10621].                                                              381196326_?->381196327_?->381196328_?->381196329_SRAP*->381196330_UmuD->381196331_UmuC->381196332_?->       424055295    <-UmuC<-UmuD<-SRAP*                                                        -                               170   bacteria>proteobacteria>gammaproteobacteria    Acinetobacter nosocomialis Ab22222                                           hypothetical protein W9I_01694 [Acinetobacter nosocomialis Ab22222].                                                           <-424055292_?<-424055293_UmuC<-424055294_UmuD<-424055295_SRAP*<-424055296_?<-424055297_?||424055298_?->       299769834    <-UmuC<-UmuD<-?<-SRAP*                                                     AOLE_07980                      208   bacteria>proteobacteria>gammaproteobacteria    Acinetobacter oleivorans DR1                                                 hypothetical protein AOLE_07980 [Acinetobacter oleivorans DR1].                                                                <-299769831_UmuC<-299769832_UmuD<-299769833_?<-299769834_SRAP*||299769835_?-><-299769836_?<-299769837_?       299771208    SRAP*->UmuD->UmuC->                                                        AOLE_14870                      211   bacteria>proteobacteria>gammaproteobacteria    Acinetobacter oleivorans DR1                                                 hypothetical protein AOLE_14870 [Acinetobacter oleivorans DR1].                                                                <-299771205_?<-299771206_?<-299771207_?||299771208_SRAP*->299771209_UmuD->299771210_UmuC->299771211_?->       406036185    SRAP*->UmuD->UmuC->                                                        AparD1_010200004361             214   bacteria>proteobacteria>gammaproteobacteria    Acinetobacter parvus DSM 16617 = CIP 108168                                  hypothetical protein AparD1_04361 [Acinetobacter parvus DSM 16617 = CIP 108168].                                               <-406036182_?||406036183_?->406036184_?->406036185_SRAP*->406036186_UmuD->406036187_UmuC->406036188_?->       480072772    <-UmuC<-UmuD<-SRAP*                                                        F930_01434                      211   bacteria>proteobacteria>gammaproteobacteria    Acinetobacter pittii ANC 3678                                                hypothetical protein F930_01434 [Acinetobacter pittii ANC 3678].                                                               <-480072769_?<-480072770_UmuC<-480072771_UmuD<-480072772_SRAP*<-480072773_?<-480072774_?<-480072775_?       480016678    <-UmuC<-UmuD<-SRAP*                                                        F951_01213                      214   bacteria>proteobacteria>gammaproteobacteria    Acinetobacter soli CIP 110264                                                hypothetical protein F951_01213 [Acinetobacter soli CIP 110264].                                                               <-480016675_?<-480016676_UmuC<-480016677_UmuD<-480016678_SRAP*<-480016679_?<-480016680_?<-480016681_?       480343471    <-UmuC<-UmuD<-SRAP*                                                        F885_00355                      214   bacteria>proteobacteria>gammaproteobacteria    Acinetobacter sp. ANC 3880                                                   hypothetical protein F885_00355 [Acinetobacter sp. ANC 3880].                                                                  <-480343468_?<-480343469_UmuC<-480343470_UmuD<-480343471_SRAP*<-480343472_?||480343473_?->480343474_?->       480160476    SRAP*-><-?||UmuD->UmuC->                                                   F904_00150                      211   bacteria>proteobacteria>gammaproteobacteria    Acinetobacter sp. ANC 4105                                                   hypothetical protein F904_00150 [Acinetobacter sp. ANC 4105].                                                                  480160473_?->480160474_?-><-480160475_?||480160476_SRAP*-><-480160477_?||480160478_UmuD->480160479_UmuC->       480352285    SRAP*->UmuD->UmuC->                                                        F884_00051                      216   bacteria>proteobacteria>gammaproteobacteria    Acinetobacter sp. CIP 102143                                                 hypothetical protein F884_00051 [Acinetobacter sp. CIP 102143].                                                                <-480352284_?||480352285_SRAP*->480352286_UmuD->480352287_UmuC->480352288_?->       480180670    SRAP*->UmuD->UmuC->                                                        F894_00054                      213   bacteria>proteobacteria>gammaproteobacteria    Acinetobacter sp. CIP 51.11                                                  hypothetical protein F894_00054 [Acinetobacter sp. CIP 51.11].                                                                 <-480180667_?||480180668_?->480180669_?->480180670_SRAP*->480180671_UmuD->480180672_UmuC->480180673_?->       479963070    UmuD->SRAP*->UmuC->                                                        F966_03615                      214   bacteria>proteobacteria>gammaproteobacteria    Acinetobacter sp. CIP 56.2                                                   hypothetical protein F966_03615 [Acinetobacter sp. CIP 56.2].                                                                  <-479963067_?<-479963068_?||479963069_UmuD->479963070_SRAP*->479963071_UmuC->479963072_?-><-479963073_?       479964365    <-UmuC<-UmuD<-SRAP*<-?||?->UmuD->                                          F966_02645                      214   bacteria>proteobacteria>gammaproteobacteria    Acinetobacter sp. CIP 56.2                                                   hypothetical protein F966_02645 [Acinetobacter sp. CIP 56.2].                                                                  <-479964362_?<-479964363_UmuC<-479964364_UmuD<-479964365_SRAP*<-479964366_?||479964367_?->479964368_UmuD->       480337926    SRAP*->UmuD->UmuC->                                                        F902_02531                      214   bacteria>proteobacteria>gammaproteobacteria    Acinetobacter sp. CIP 70.18                                                  hypothetical protein F902_02531 [Acinetobacter sp. CIP 70.18].                                                                 480337923_?-><-480337924_?<-480337925_?||480337926_SRAP*->480337927_UmuD->480337928_UmuC-><-480337929_?       402759048    UmuD->SRAP*->                                                              ANCT7_010100015330              214   bacteria>proteobacteria>gammaproteobacteria    Acinetobacter sp. NCTC 7422                                                  hypothetical protein ANCT7_15330 [Acinetobacter sp. NCTC 7422].                                                                <-402759045_?<-402759046_?||402759047_UmuD->402759048_SRAP*-><-402759049_?<-402759050_?<-402759051_?       480169651    SRAP*->UmuD->UmuC->                                                        F898_03189                      215   bacteria>proteobacteria>gammaproteobacteria    Acinetobacter sp. NIPH 1847                                                  hypothetical protein F898_03189 [Acinetobacter sp. NIPH 1847].                                                                 <-480169648_?<-480169649_?||480169650_?->480169651_SRAP*->480169652_UmuD->480169653_UmuC->480169654_?->       480169896    SRAP*->UmuD->UmuC->                                                        F898_03438                      214   bacteria>proteobacteria>gammaproteobacteria    Acinetobacter sp. NIPH 1847                                                  hypothetical protein F898_03438 [Acinetobacter sp. NIPH 1847].                                                                 <-480169893_?||480169894_?->480169895_?->480169896_SRAP*->480169897_UmuD->480169898_UmuC->480169899_?->       480198927    SRAP*->UmuD->UmuC->                                                        F889_01517                      214   bacteria>proteobacteria>gammaproteobacteria    Acinetobacter sp. NIPH 1859                                                  hypothetical protein F889_01517 [Acinetobacter sp. NIPH 1859].                                                                 480198924_?->480198925_?->480198926_?->480198927_SRAP*->480198928_UmuD->480198929_UmuC->480198930_?->       480186208    <-UmuC<-UmuD<-?<-SRAP*                                                     F892_01730                      210   bacteria>proteobacteria>gammaproteobacteria    Acinetobacter sp. NIPH 2168                                                  hypothetical protein F892_01730 [Acinetobacter sp. NIPH 2168].                                                                 <-480186205_UmuC<-480186206_UmuD<-480186207_?<-480186208_SRAP*<-480186209_?<-480186210_?||480186211_?->       480172262    <-UmuC<-UmuD<-SRAP*                                                        F897_01968                      211   bacteria>proteobacteria>gammaproteobacteria    Acinetobacter sp. NIPH 2171                                                  hypothetical protein F897_01968 [Acinetobacter sp. NIPH 2171].                                                                 480172259_?-><-480172260_UmuC<-480172261_UmuD<-480172262_SRAP*<-480172263_?||480172264_?->480172265_?->       480212543    SRAP*->UmuD->UmuC->                                                        F886_00025                      211   bacteria>proteobacteria>gammaproteobacteria    Acinetobacter sp. NIPH 542                                                   hypothetical protein F886_00025 [Acinetobacter sp. NIPH 542].                                                                  480212540_?->480212541_?->480212542_?->480212543_SRAP*->480212544_UmuD->480212545_UmuC-><-480212546_?       479958412    <-UmuC<-UmuD<-?<-SRAP*                                                     F968_01167                      212   bacteria>proteobacteria>gammaproteobacteria    Acinetobacter sp. NIPH 817                                                   hypothetical protein F968_01167 [Acinetobacter sp. NIPH 817].                                                                  <-479958409_UmuC<-479958410_UmuD<-479958411_?<-479958412_SRAP*<-479958413_?<-479958414_?<-479958415_?       479952171    SRAP*->UmuD->UmuC->                                                        F969_03271                      211   bacteria>proteobacteria>gammaproteobacteria    Acinetobacter sp. NIPH 899                                                   hypothetical protein F969_03271 [Acinetobacter sp. NIPH 899].                                                                  <-479952168_?<-479952169_?<-479952170_?||479952171_SRAP*->479952172_UmuD->479952173_UmuC-><-479952174_?       358013322    <-UmuC<-UmuD<-SRAP*                                                        AP8-3_010100017542              214   bacteria>proteobacteria>gammaproteobacteria    Acinetobacter sp. P8-3-8                                                     hypothetical protein AP8-3_17542 [Acinetobacter sp. P8-3-8].                                                                   <-358013319_?<-358013320_UmuC<-358013321_UmuD<-358013322_SRAP*<-358013323_?||358013324_?->       260551644    <-UmuC<-UmuD<-SRAP*                                                        HMPREF0014_02732                214   bacteria>proteobacteria>gammaproteobacteria    Acinetobacter sp. RUH2624                                                    conserved hypothetical protein [Acinetobacter sp. RUH2624].                                                                    <-260551641_?<-260551642_UmuC<-260551643_UmuD<-260551644_SRAP*<-260551645_?<-260551646_?||260551647_?->       445419133    SRAP*->UmuD->UmuC->                                                        ACINWC743_A0819                 214   bacteria>proteobacteria>gammaproteobacteria    Acinetobacter sp. WC-743                                                     hypothetical protein ACINWC743_A0819 [Acinetobacter sp. WC-743].                                                               <-445419182_?<-445419161_?||445419127_?->445419133_SRAP*->445419141_UmuD->445419173_UmuC->445419107_?->       406041431    SRAP*->UmuD->UmuC->                                                        AursD1_010200016789             200   bacteria>proteobacteria>gammaproteobacteria    Acinetobacter ursingii DSM 16037 = CIP 107286                                hypothetical protein AursD1_16789 [Acinetobacter ursingii DSM 16037 = CIP 107286].                                             <-406041428_?<-406041429_?||406041430_?->406041431_SRAP*->406041432_UmuD->406041433_UmuC->406041434_?->       406041410    SRAP*->UmuD->UmuC-><-SRAP                                                  AursD1_010200016684             213   bacteria>proteobacteria>gammaproteobacteria    Acinetobacter ursingii DSM 16037 = CIP 107286                                hypothetical protein AursD1_16684 [Acinetobacter ursingii DSM 16037 = CIP 107286].                                             <-406041407_?||406041408_?->406041409_?->406041410_SRAP*->406041411_UmuD->406041412_UmuC-><-406041413_SRAP       480037803    <-UmuC<-UmuD<-SRAP*<-?||?->SRAP->                                          F944_00271                      213   bacteria>proteobacteria>gammaproteobacteria    Acinetobacter ursingii DSM 16037 = CIP 107286                                hypothetical protein F944_00271 [Acinetobacter ursingii DSM 16037 = CIP 107286].                                               <-480037800_?<-480037801_UmuC<-480037802_UmuD<-480037803_SRAP*<-480037804_?||480037805_?->480037806_SRAP->       480209336    <-UmuC<-UmuD<-SRAP*                                                        F943_03396                      213   bacteria>proteobacteria>gammaproteobacteria    Acinetobacter ursingii NIPH 706                                              hypothetical protein F943_03396 [Acinetobacter ursingii NIPH 706].                                                             <-480209333_?<-480209334_UmuC<-480209335_UmuD<-480209336_SRAP*<-480209337_?<-480209338_?<-480209339_?       333894798    <-SRAP*||UmuD->UmuC->                                                      -                               230   bacteria>proteobacteria>gammaproteobacteria    Alteromonas sp. SN2                                                          hypothetical protein ambt_16830 [Alteromonas sp. SN2].                                                                         333894795_?-><-333894796_?<-333894797_?<-333894798_SRAP*||333894799_UmuD->333894800_UmuC->333894801_?->       365104453    <-DAM||SRAP*->UmuD->UmuC->                                                 HMPREF9428_04694                223   bacteria>proteobacteria>gammaproteobacteria    Citrobacter freundii 4_7_47CFAA                                              hypothetical protein HMPREF9428_04694 [Citrobacter freundii 4_7_47CFAA].                                                       <-365104450_?<-365104451_?<-365104452_DAM||365104453_SRAP*->365104454_UmuD->365104455_UmuC-><-365104456_?       237731167    UmuD->UmuC-><-SRAP*                                                        CSAG_00978                      223   bacteria>proteobacteria>gammaproteobacteria    Citrobacter sp. 30_2                                                         conserved hypothetical protein [Citrobacter sp. 30_2].                                                                         <-237731164_?||237731165_UmuD->237731166_UmuC-><-237731167_SRAP*||237731168_?->237731169_?->237731170_?->       395231193    <-DAM||SRAP*->UmuD->UmuC->                                                 WYG_4146                        223   bacteria>proteobacteria>gammaproteobacteria    Citrobacter sp. A1                                                           hypothetical protein WYG_4146 [Citrobacter sp. A1].                                                                            <-395231190_?<-395231191_?<-395231192_DAM||395231193_SRAP*->395231194_UmuD->395231195_UmuC-><-395231196_?       395229604    SRAP*-><-?<-UmuD                                                           WYG_2553                        223   bacteria>proteobacteria>gammaproteobacteria    Citrobacter sp. A1                                                           hypothetical protein WYG_2553 [Citrobacter sp. A1].                                                                            395229601_?-><-395229602_?||395229603_?->395229604_SRAP*-><-395229605_?<-395229606_UmuD||395229607_?->       365970608    SRAP*-><-UmuC<-UmuD                                                        -                               222   bacteria>proteobacteria>gammaproteobacteria    Enterobacter cloacae EcWSU1                                                  protein YedK [Enterobacter cloacae EcWSU1].                                                                                    <-365970605_?||365970606_?-><-365970607_?||365970608_SRAP*-><-365970609_UmuC<-365970610_UmuD<-365970611_?       365970713    SRAP*->?-><-UmuC<-UmuD                                                     -                               213   bacteria>proteobacteria>gammaproteobacteria    Enterobacter cloacae EcWSU1                                                  protein YedK [Enterobacter cloacae EcWSU1].                                                                                    <-365970710_?<-365970711_?<-365970712_?||365970713_SRAP*->365970714_?-><-365970715_UmuC<-365970716_UmuD       401763836    SRAP*-><-?<-UmuC<-UmuD                                                     -                               224   bacteria>proteobacteria>gammaproteobacteria    Enterobacter cloacae subsp. cloacae ENHKU01                                  hypothetical protein ECENHK_11790 [Enterobacter cloacae subsp. cloacae ENHKU01].                                               <-401763833_?<-401763834_?<-401763835_?||401763836_SRAP*-><-401763837_?<-401763838_UmuC<-401763839_UmuD       392979329    SRAP*->?-><-UmuC<-UmuD                                                     -                               227   bacteria>proteobacteria>gammaproteobacteria    Enterobacter cloacae subsp. dissolvens SDM                                   hypothetical protein A3UG_12435 [Enterobacter cloacae subsp. dissolvens SDM].                                                  <-392979326_?||392979327_?->392979328_?->392979329_SRAP*->392979330_?-><-392979331_UmuC<-392979332_UmuD       311279195    UmuD->UmuC-><-SRAP*                                                        -                               223   bacteria>proteobacteria>gammaproteobacteria    Enterobacter lignolyticus SCF1                                               hypothetical protein Entcl_1886 [Enterobacter lignolyticus SCF1].                                                              311279192_?->311279193_UmuD->311279194_UmuC-><-311279195_SRAP*||311279196_?-><-311279197_?||311279198_?->       395233074    <-DAM||SRAP*->UmuD->UmuC->                                                 A936_05440                      223   bacteria>proteobacteria>gammaproteobacteria    Enterobacter sp. Ag1                                                         hypothetical protein A936_05440 [Enterobacter sp. Ag1].                                                                        <-395233071_?<-395233072_?<-395233073_DAM||395233074_SRAP*->395233075_UmuD->395233076_UmuC->       458930851    UmuD->UmuC-><-SRAP*                                                        ESO_RS07400                     228   bacteria>proteobacteria>gammaproteobacteria    Escherichia albertii TW11588                                                 hypothetical protein ESO_RS07400 [Escherichia albertii TW11588].                                                               <-458930848_?||458930849_UmuD->458930850_UmuC-><-458930851_SRAP*<-458930852_?<-458930853_?<-458930854_?       432362104    UmuD->UmuC-><-SRAP*                                                        WCE_01131                       223   bacteria>proteobacteria>gammaproteobacteria    Escherichia coli KTE5                                                        hypothetical protein WCE_01131 [Escherichia coli KTE5].                                                                        <-432362101_?||432362102_UmuD->432362103_UmuC-><-432362104_SRAP*||432362105_?-><-432362106_?<-432362107_?       458003515    SRAP*->UmuD->UmuC->                                                        EQQ_RS16890                     223   bacteria>proteobacteria>gammaproteobacteria    Escherichia sp. TW09231                                                      hypothetical protein EQQ_RS16890 [Escherichia sp. TW09231].                                                                    <-458003514_?||458003515_SRAP*->458003516_UmuD->458003517_UmuC-><-458003518_?       159528149    <-UmuC<-UmuC<-UmuD<-SRAP*                                                  pLDTEXKL_p54                    222   bacteria>proteobacteria>gammaproteobacteria    Fluoribacter dumoffii Tex-KL                                                 conserved hypothetical protein [Fluoribacter dumoffii Tex-KL].                                                                 <-159528146_UmuC<-159528147_UmuC<-159528148_UmuD<-159528149_SRAP*<-159528150_?<-159528151_?<-159528152_?       410625130    <-UmuC<-UmuD||SRAP*->                                                      GMES_0378                       229   bacteria>proteobacteria>gammaproteobacteria    Glaciecola mesophila KMM 241                                                 hypothetical protein GMES_0378 [Glaciecola mesophila KMM 241].                                                                 410625127_?-><-410625128_UmuC<-410625129_UmuD||410625130_SRAP*->410625131_?-><-410625132_?<-410625133_?       423108537    SRAP*->?-><-UmuC<-UmuD                                                     -                               223   bacteria>proteobacteria>gammaproteobacteria    Klebsiella oxytoca 10-5243                                                   hypothetical protein HMPREF9687_01783 [Klebsiella oxytoca 10-5243].                                                            423108534_?-><-423108535_?<-423108536_?||423108537_SRAP*->423108538_?-><-423108539_UmuC<-423108540_UmuD       423114563    SRAP*->?-><-UmuC<-UmuD                                                     -                               223   bacteria>proteobacteria>gammaproteobacteria    Klebsiella oxytoca 10-5245                                                   hypothetical protein HMPREF9689_02311 [Klebsiella oxytoca 10-5245].                                                            <-423114560_?<-423114561_?||423114562_?->423114563_SRAP*->423114564_?-><-423114565_UmuC<-423114566_UmuD       474884541    <-DAM||?->SRAP*->UmuD->UmuC->                                              KPC_048                         223   bacteria>proteobacteria>gammaproteobacteria    Klebsiella pneumoniae                                                        Gifsy-2 prophage protein [Klebsiella pneumoniae].                                                                              <-474884538_?<-474884539_DAM||474884540_?->474884541_SRAP*->474884542_UmuD->474884543_UmuC-><-474884544_?       206577616    SRAP*-><-UmuC<-UmuD                                                        -                               223   bacteria>proteobacteria>gammaproteobacteria    Klebsiella pneumoniae 342                                                    hypothetical protein KPK_3126 [Klebsiella pneumoniae 342].                                                                     206579496_?->206579760_?->206580306_?->206577616_SRAP*-><-206578390_UmuC<-206576780_UmuD<-206578377_?       472293282    UmuD->UmuC-><-?<-SRAP*                                                     KP700603_10654                  225   bacteria>proteobacteria>gammaproteobacteria    Klebsiella pneumoniae 700603                                                 hypothetical protein KP700603_10654 [Klebsiella pneumoniae 700603].                                                            472293279_UmuD->472293280_UmuC-><-472293281_?<-472293282_SRAP*||472293283_?-><-472293284_?<-472293285_?       470928763    SRAP*-><-UmuD                                                              -                               184   bacteria>proteobacteria>gammaproteobacteria    Klebsiella pneumoniae RYC492                                                 hypothetical protein KPRYC492_14730 [Klebsiella pneumoniae RYC492].                                                            470928760_?->470928761_?->470928762_?->470928763_SRAP*-><-470928764_UmuD<-470928765_?||470928766_?->       470928662    SRAP*-><-UmuC<-UmuD                                                        -                               225   bacteria>proteobacteria>gammaproteobacteria    Klebsiella pneumoniae RYC492                                                 hypothetical protein KPRYC492_14105 [Klebsiella pneumoniae RYC492].                                                            470928659_?->470928660_?->470928661_?->470928662_SRAP*-><-470928663_UmuC<-470928664_UmuD<-470928665_?       419976225    UmuD->UmuC-><-SRAP*                                                        KPNIH1_22814                    223   bacteria>proteobacteria>gammaproteobacteria    Klebsiella pneumoniae subsp. pneumoniae KPNIH1                               hypothetical protein KPNIH1_22814 [Klebsiella pneumoniae subsp. pneumoniae KPNIH1].                                            419976222_?->419976223_UmuD->419976224_UmuC-><-419976225_SRAP*<-419976226_?<-419976227_?<-419976228_?       425081249    UmuD->UmuC->?-><-SRAP*                                                     -                               230   bacteria>proteobacteria>gammaproteobacteria    Klebsiella pneumoniae subsp. pneumoniae WGLW2                                hypothetical protein HMPREF1306_01997 [Klebsiella pneumoniae subsp. pneumoniae WGLW2].                                         425081246_UmuD->425081247_UmuC->425081248_?-><-425081249_SRAP*<-425081250_?<-425081251_?<-425081252_?       290509913    SRAP*-><-UmuC<-UmuD                                                        HMPREF0485_01684                223   bacteria>proteobacteria>gammaproteobacteria    Klebsiella sp. 1_1_55                                                        hypothetical protein HMPREF0485_01684 [Klebsiella sp. 1_1_55].                                                                 290509910_?->290509911_?->290509912_?->290509913_SRAP*-><-290509914_UmuC<-290509915_UmuD<-290509916_?       290512886    SRAP*->?-><-UmuC<-UmuD                                                     HMPREF0485_04655                223   bacteria>proteobacteria>gammaproteobacteria    Klebsiella sp. 1_1_55                                                        hypothetical protein HMPREF0485_04655 [Klebsiella sp. 1_1_55].                                                                 <-290512883_?<-290512884_?<-290512885_?||290512886_SRAP*->290512887_?-><-290512888_UmuC<-290512889_UmuD       270159933    SRAP*->UmuD->UmuC->                                                        -                               222   bacteria>proteobacteria>gammaproteobacteria    Legionella longbeachae D-4968                                                conserved hypothetical protein [Legionella longbeachae D-4968].                                                                270159930_?-><-270159931_?||270159932_?->270159933_SRAP*->270159934_UmuD->270159935_UmuC->270159936_?->       270160320    SRAP*->UmuD->UmuC->UmuC->                                                  LLB_3782                        221   bacteria>proteobacteria>gammaproteobacteria    Legionella longbeachae D-4968                                                conserved hypothetical protein [Legionella longbeachae D-4968].                                                                270160317_?-><-270160318_?||270160319_?->270160320_SRAP*->270160321_UmuD->270160322_UmuC->270160323_UmuC->       289165319    <-UmuC<-UmuD<-SRAP*                                                        -                               222   bacteria>proteobacteria>gammaproteobacteria    Legionella longbeachae NSW150                                                hypothetical protein LLO_1988 [Legionella longbeachae NSW150].                                                                 <-289165316_?<-289165317_UmuC<-289165318_UmuD<-289165319_SRAP*||289165320_?-><-289165321_?<-289165322_?       289165201    <-UmuC<-UmuC<-UmuD<-SRAP*                                                  -                               222   bacteria>proteobacteria>gammaproteobacteria    Legionella longbeachae NSW150                                                hypothetical protein LLO_1864 [Legionella longbeachae NSW150].                                                                 <-289165198_UmuC<-289165199_UmuC<-289165200_UmuD<-289165201_SRAP*<-289165202_?||289165203_?->289165204_?->       296106560    SRAP*->UmuD->UmuC->                                                        lpa_01467                       222   bacteria>proteobacteria>gammaproteobacteria    Legionella pneumophila 2300/99 Alcoy                                         hypothetical protein lpa_01467 [Legionella pneumophila 2300/99 Alcoy].                                                         296106557_?->296106558_?->296106559_?->296106560_SRAP*->296106561_UmuD->296106562_UmuC->296106563_?->       54293946     SRAP*->UmuD->UmuC->                                                        -                               222   bacteria>proteobacteria>gammaproteobacteria    Legionella pneumophila str. Lens                                             hypothetical protein lpl1003 [Legionella pneumophila str. Lens].                                                               54293943_?->54293944_?->54293945_?->54293946_SRAP*->54293947_UmuD->54293948_UmuC->54293949_?->       54292963     <-UmuC<-UmuD<-SRAP*                                                        plpl0057                        222   bacteria>proteobacteria>gammaproteobacteria    Legionella pneumophila str. Lens                                             hypothetical protein plpl0057 [Legionella pneumophila str. Lens].                                                              <-54292960_?<-54292961_UmuC<-54292962_UmuD<-54292963_SRAP*       52841462     SRAP*->UmuD->UmuC->                                                        -                               222   bacteria>proteobacteria>gammaproteobacteria    Legionella pneumophila subsp. pneumophila str. Philadelphia 1                hypothetical protein lpg1230 [Legionella pneumophila subsp. pneumophila str. Philadelphia 1].                                  52841459_?->52841460_?->52841461_?->52841462_SRAP*->52841463_UmuD->52841464_UmuC-><-52841465_?       387812932    SRAP*->?->?-><-UmuD                                                        -                               224   bacteria>proteobacteria>gammaproteobacteria    Marinobacter hydrocarbonoclasticus ATCC 49840                                hypothetical protein MARHY0490 [Marinobacter hydrocarbonoclasticus ATCC 49840].                                                387812929_?-><-387812930_?||387812931_?->387812932_SRAP*->387812933_?->387812934_?-><-387812935_UmuD       333983690    <-UmuC<-UmuD||?->SRAP*->                                                   -                               221   bacteria>proteobacteria>gammaproteobacteria    Methylomonas methanica MC09                                                  hypothetical protein [Methylomonas methanica MC09].                                                                            <-333983687_UmuC<-333983688_UmuD||333983689_?->333983690_SRAP*->333983691_?->333983692_?->333983693_?->       353334426    <-SRAP*||UmuD->                                                            -                               227   bacteria>proteobacteria>gammaproteobacteria    Pseudomonas aeruginosa                                                       Gifsy-2 prophage protein [Pseudomonas aeruginosa].                                                                             <-353334424_?||353334425_?-><-353334506_?<-353334426_SRAP*||353334427_UmuD->353334428_?->353334429_?->       478480910    <-UmuC<-UmuD||SRAP*->                                                      -                               227   bacteria>proteobacteria>gammaproteobacteria    Pseudomonas aeruginosa B136-33                                               hypothetical protein G655_23625 [Pseudomonas aeruginosa B136-33].                                                              478480907_?-><-478480908_UmuC<-478480909_UmuD||478480910_SRAP*-><-478480911_?||478480912_?->478480913_?->       254238744    <-SRAP*||UmuD->UmuC->                                                      PACG_04908                      227   bacteria>proteobacteria>gammaproteobacteria    Pseudomonas aeruginosa C3719                                                 hypothetical protein PACG_04908 [Pseudomonas aeruginosa C3719].                                                                <-254238741_?<-254238742_?<-254238743_?<-254238744_SRAP*||254238745_UmuD->254238746_UmuC-><-254238747_?       458878590    <-SRAP*||UmuD->                                                            H123_01280                      227   bacteria>proteobacteria>gammaproteobacteria    Pseudomonas aeruginosa PA21_ST175                                            hypothetical protein H123_01280 [Pseudomonas aeruginosa PA21_ST175].                                                           458878587_?-><-458878588_?||458878589_?-><-458878590_SRAP*||458878591_UmuD->458878592_?-><-458878593_?       452878104    <-SRAP*||UmuD->                                                            G039_14678                      230   bacteria>proteobacteria>gammaproteobacteria    Pseudomonas aeruginosa VRFPA01                                               hypothetical protein G039_14678 [Pseudomonas aeruginosa VRFPA01].                                                              452878103_?-><-452878104_SRAP*||452878105_UmuD->452878106_?->452878107_?->       395652512    <-SRAP*<-SRAP||UmuD->UmuC->                                                Pext1s1_010100028193            142   bacteria>proteobacteria>gammaproteobacteria    Pseudomonas extremaustralis 14-3 substr. 14-3b                               hypothetical protein Pext1s1_28193 [Pseudomonas extremaustralis 14-3 substr. 14-3b].                                           395652509_?-><-395652510_?<-395652511_?<-395652512_SRAP*<-395652513_SRAP||395652514_UmuD->395652515_UmuC-><-395652516_?       421137798    <-SRAP*||UmuD->UmuC->                                                      MHB_01030                       233   bacteria>proteobacteria>gammaproteobacteria    Pseudomonas fluorescens BBc6R8                                               hypothetical protein MHB_01030 [Pseudomonas fluorescens BBc6R8].                                                               421137795_?->421137796_?->421137797_?-><-421137798_SRAP*||421137799_UmuD->421137800_UmuC->421137801_?->       421142470    <-UmuC<-UmuD||SRAP*->                                                      MHB_24000                       232   bacteria>proteobacteria>gammaproteobacteria    Pseudomonas fluorescens BBc6R8                                               hypothetical protein MHB_24000 [Pseudomonas fluorescens BBc6R8].                                                               <-421142467_?<-421142468_UmuC<-421142469_UmuD||421142470_SRAP*-><-421142471_?||421142472_?->421142473_?->       378951195    <-SRAP*||UmuD->UmuC->                                                      -                               215   bacteria>proteobacteria>gammaproteobacteria    Pseudomonas fluorescens F113                                                 Gifsy-2 prophage protein [Pseudomonas fluorescens F113].                                                                       378951192_?->378951193_?->378951194_?-><-378951195_SRAP*||378951196_UmuD->378951197_UmuC-><-378951198_?       378952186    <-UmuC<-UmuD||SRAP*-><-methyltransferase                                   -                               134   bacteria>proteobacteria>gammaproteobacteria    Pseudomonas fluorescens F113                                                 Gifsy-2 prophage protein [Pseudomonas fluorescens F113].                                                                       378952183_?-><-378952184_UmuC<-378952185_UmuD||378952186_SRAP*-><-378952187_methyltransferase<-378952188_?<-378952189_?       423691819    <-SRAP*||UmuD->UmuC->                                                      -                               236   bacteria>proteobacteria>gammaproteobacteria    Pseudomonas fluorescens SS101                                                protein of unknown function, DUF159 family [Pseudomonas fluorescens SS101].                                                    423691816_?->423691817_?-><-423691818_?<-423691819_SRAP*||423691820_UmuD->423691821_UmuC->423691822_?->       402702075    <-UmuC<-UmuD||SRAP*->                                                      PfraA_010100019673              236   bacteria>proteobacteria>gammaproteobacteria    Pseudomonas fragi A22                                                        hypothetical protein PfraA_19673 [Pseudomonas fragi A22].                                                                      <-402702072_?<-402702073_UmuC<-402702074_UmuD||402702075_SRAP*-><-402702076_?<-402702077_?<-402702078_?       333899671    methyltransferase->?-><-SRAP*||UmuD->UmuC->                                -                               231   bacteria>proteobacteria>gammaproteobacteria    Pseudomonas fulva 12-X                                                       hypothetical protein Psefu_1474 [Pseudomonas fulva 12-X].                                                                      <-333899668_?||333899669_methyltransferase->333899670_?-><-333899671_SRAP*||333899672_UmuD->333899673_UmuC-><-333899674_?       421506208    <-SRAP*||UmuD->UmuC->DinB->                                                A471_23113                      218   bacteria>proteobacteria>gammaproteobacteria    Pseudomonas mendocina DLHK                                                   hypothetical protein A471_23113 [Pseudomonas mendocina DLHK].                                                                  421506205_?-><-421506206_?||421506207_?-><-421506208_SRAP*||421506209_UmuD->421506210_UmuC->421506211_DinB->       70729493     <-SRAP*<-?<-?<-UmuD                                                        -                               225   bacteria>proteobacteria>gammaproteobacteria    Pseudomonas protegens Pf-5                                                   hypothetical protein PFL_2124 [Pseudomonas protegens Pf-5].                                                                    70729489_?->70729490_?->70729492_?-><-70729493_SRAP*<-346642876_?<-70729494_?<-70729495_UmuD       429332680    <-UmuC<-UmuD||SRAP*->                                                      CSV86_12675                     230   bacteria>proteobacteria>gammaproteobacteria    Pseudomonas putida CSV86                                                     hypothetical protein CSV86_12675 [Pseudomonas putida CSV86].                                                                   <-429332678_UmuC<-429332679_UmuD||429332680_SRAP*-><-429332681_?<-429332682_?||429332683_?->       397696939    <-SRAP*||UmuD->UmuC->                                                      T1E_4199                        236   bacteria>proteobacteria>gammaproteobacteria    Pseudomonas putida DOT-T1E                                                   hypothetical protein T1E_4199 [Pseudomonas putida DOT-T1E].                                                                    <-397696936_?<-397696937_?<-397696938_?<-397696939_SRAP*||397696940_UmuD->397696941_UmuC->397696942_?->       148548162    <-UmuC<-UmuD||SRAP*->                                                      -                               242   bacteria>proteobacteria>gammaproteobacteria    Pseudomonas putida F1                                                        hypothetical protein Pput_2952 [Pseudomonas putida F1].                                                                        <-148548159_?<-148548160_UmuC<-148548161_UmuD||148548162_SRAP*->148548163_?->148548164_?->148548165_?->       170721920    <-UmuC<-UmuD||SRAP*->                                                      -                               239   bacteria>proteobacteria>gammaproteobacteria    Pseudomonas putida W619                                                      hypothetical protein PputW619_2747 [Pseudomonas putida W619].                                                                  <-170721917_?<-170721918_UmuC<-170721919_UmuD||170721920_SRAP*->170721921_?->170721922_?->170721923_?->       409397416    <-UmuC<-UmuD||SRAP*->                                                      C211_17989                      230   bacteria>proteobacteria>gammaproteobacteria    Pseudomonas sp. Chol1                                                        hypothetical protein C211_17989 [Pseudomonas sp. Chol1].                                                                       <-409397413_?<-409397414_UmuC<-409397415_UmuD||409397416_SRAP*->       398875355    <-SRAP*||UmuD->UmuC->                                                      PMI33_00172                     209   bacteria>proteobacteria>gammaproteobacteria    Pseudomonas sp. GM67                                                         hypothetical protein PMI33_00172 [Pseudomonas sp. GM67].                                                                       398875352_?->398875353_?->398875354_?-><-398875355_SRAP*||398875356_UmuD->398875357_UmuC-><-398875358_?       398848681    <-UmuC||?-><-UmuD||SRAP*->                                                 PMI38_04923                     239   bacteria>proteobacteria>gammaproteobacteria    Pseudomonas sp. GM84                                                         hypothetical protein PMI38_04923 [Pseudomonas sp. GM84].                                                                       <-398848678_UmuC||398848679_?-><-398848680_UmuD||398848681_SRAP*-><-398848682_?       325272249    <-SRAP*||UmuD->UmuC->                                                      G1E_05118                       239   bacteria>proteobacteria>gammaproteobacteria    Pseudomonas sp. TJI-51                                                       hypothetical protein G1E_05118 [Pseudomonas sp. TJI-51].                                                                       <-325272246_?||325272247_?->325272248_?-><-325272249_SRAP*||325272250_UmuD->325272251_UmuC->       146283673    <-UmuC<-UmuD||SRAP*->                                                      -                               237   bacteria>proteobacteria>gammaproteobacteria    Pseudomonas stutzeri A1501                                                   hypothetical protein PST_3356 [Pseudomonas stutzeri A1501].                                                                    <-146283670_?<-146283671_UmuC<-146283672_UmuD||146283673_SRAP*->146283674_?-><-146283675_?||146283676_?->       339494870    <-UmuC<-UmuD||SRAP*->                                                      -                               249   bacteria>proteobacteria>gammaproteobacteria    Pseudomonas stutzeri ATCC 17588 = LMG 11199                                  hypothetical protein PSTAB_2793 [Pseudomonas stutzeri ATCC 17588 = LMG 11199].                                                 339494867_?-><-339494868_UmuC<-339494869_UmuD||339494870_SRAP*->339494871_?-><-339494872_?<-339494873_?       392422619    <-UmuC<-UmuD||SRAP*->                                                      A458_17875                      237   bacteria>proteobacteria>gammaproteobacteria    Pseudomonas stutzeri CCUG 29243                                              hypothetical protein A458_17875 [Pseudomonas stutzeri CCUG 29243].                                                             392422616_?-><-392422617_UmuC<-392422618_UmuD||392422619_SRAP*-><-392422620_?||392422621_?->392422622_?->       419956359    <-UmuC<-UmuD||SRAP*->                                                      YO5_13771                       237   bacteria>proteobacteria>gammaproteobacteria    Pseudomonas stutzeri TS44                                                    hypothetical protein YO5_13771 [Pseudomonas stutzeri TS44].                                                                    <-419956356_?<-419956357_UmuC<-419956358_UmuD||419956359_SRAP*-><-419956360_?       440724225    <-UmuC||?->SRAP*->SRAP->                                                   A979_25340                      83    bacteria>proteobacteria>gammaproteobacteria    Pseudomonas syringae BRIP34876                                               hypothetical protein A979_25340 [Pseudomonas syringae BRIP34876].                                                              <-440724222_?<-440724223_UmuC||440724224_?->440724225_SRAP*->440724226_SRAP->       440745965    ClpB-><-?<-?<-SRAP*||UmuD->UmuC->                                          A988_21172                      230   bacteria>proteobacteria>gammaproteobacteria    Pseudomonas syringae BRIP39023                                               hypothetical protein A988_21172 [Pseudomonas syringae BRIP39023].                                                              440745962_ClpB-><-440745963_?<-440745964_?<-440745965_SRAP*||440745966_UmuD->440745967_UmuC->440745968_?->       422634162    <-UmuD||SRAP*->                                                            PSYCIT7_29046                   229   bacteria>proteobacteria>gammaproteobacteria    Pseudomonas syringae Cit 7                                                   hypothetical protein PSYCIT7_29046 [Pseudomonas syringae Cit 7].                                                               <-422634161_UmuD||422634162_SRAP*->       470897576    <-SRAP*||UmuD->                                                            B195_21550                      229   bacteria>proteobacteria>gammaproteobacteria    Pseudomonas syringae Lz4W                                                    hypothetical protein B195_21550 [Pseudomonas syringae Lz4W].                                                                   470897573_?->470897574_?->470897575_?-><-470897576_SRAP*||470897577_UmuD->470897578_?-><-470897579_?       422674183    <-UmuC<-UmuD||SRAP*->                                                      PSYAR_15592                     142   bacteria>proteobacteria>gammaproteobacteria    Pseudomonas syringae pv. aceris str. M302273                                 hypothetical protein PSYAR_15592, partial [Pseudomonas syringae pv. aceris str. M302273].                                      422674180_?-><-422674181_UmuC<-422674182_UmuD||422674183_SRAP*->422674184_?->422674185_?-><-422674186_?       449041112    <-SRAP*||UmuD->UmuC->                                                      -                               230   bacteria>proteobacteria>gammaproteobacteria    Pseudomonas syringae pv. actinidiae                                          protein of unknown function DUF159 [Pseudomonas syringae pv. actinidiae].                                                      <-449041110_?<-449041111_?<-449041112_SRAP*||449041113_UmuD->449041114_UmuC->449041115_?->       449041610    <-SRAP*||UmuD->UmuC->                                                      -                               230   bacteria>proteobacteria>gammaproteobacteria    Pseudomonas syringae pv. actinidiae                                          protein of unknown function DUF159 [Pseudomonas syringae pv. actinidiae].                                                      449041607_?->449041608_?-><-449041609_?<-449041610_SRAP*||449041611_UmuD->449041612_UmuC-><-449041613_?       449041353    <-SRAP*||UmuD->UmuC->                                                      -                               230   bacteria>proteobacteria>gammaproteobacteria    Pseudomonas syringae pv. actinidiae                                          protein of unknown function DUF159 [Pseudomonas syringae pv. actinidiae].                                                      449041350_?->449041351_?-><-449041352_?<-449041353_SRAP*||449041354_UmuD->449041355_UmuC->449041356_?->       422655191    <-UmuC<-UmuC<-UmuD||SRAP*->                                                PSYAC_27346                     74    bacteria>proteobacteria>gammaproteobacteria    Pseudomonas syringae pv. actinidiae str. M302091                             hypothetical protein PSYAC_27346, partial [Pseudomonas syringae pv. actinidiae str. M302091].                                  <-422655188_UmuC<-422655189_UmuC<-422655190_UmuD||422655191_SRAP*->422655192_?->422655193_?->422655194_?->       424065896    ClpB-><-?<-?<-SRAP*||UmuD->UmuC->                                          Pav013_0750                     122   bacteria>proteobacteria>gammaproteobacteria    Pseudomonas syringae pv. avellanae str. ISPaVe013                            Protein of unknown function DUF159 [Pseudomonas syringae pv. avellanae str. ISPaVe013].                                        424065893_ClpB-><-424065894_?<-424065895_?<-424065896_SRAP*||424065897_UmuD->424065898_UmuC->424065899_?->       68637934     <-UmuC<-UmuD||SRAP*->                                                      pph98                           220   bacteria>proteobacteria>gammaproteobacteria    Pseudomonas syringae pv. phaseolicola                                        hypothetical protein [Pseudomonas syringae pv. phaseolicola].                                                                  <-68637931_?<-68637932_UmuC<-68637933_UmuD||68637934_SRAP*->68637935_?->68637936_?->       422632084    <-SRAP<-SRAP*||UmuD->SRAP->                                                PSYPI_21040                     230   bacteria>proteobacteria>gammaproteobacteria    Pseudomonas syringae pv. pisi str. 1704B                                     hypothetical protein PSYPI_21040 [Pseudomonas syringae pv. pisi str. 1704B].                                                   <-422632081_?<-422632082_?<-422632083_SRAP<-422632084_SRAP*||422632085_UmuD->422632086_SRAP-><-422632087_?       66045999     <-UmuC<-UmuD||SRAP*->                                                      -                               229   bacteria>proteobacteria>gammaproteobacteria    Pseudomonas syringae pv. syringae B728a                                      hypothetical protein Psyr_2763 [Pseudomonas syringae pv. syringae B728a].                                                      66045996_?-><-66045997_UmuC<-66045998_UmuD||66045999_SRAP*-><-66046000_?<-66046001_?<-66046002_?       66044671     <-SRAP*||UmuD->UmuC->                                                      -                               230   bacteria>proteobacteria>gammaproteobacteria    Pseudomonas syringae pv. syringae B728a                                      hypothetical protein Psyr_1423 [Pseudomonas syringae pv. syringae B728a].                                                      66044668_?-><-66044669_?<-66044670_?<-66044671_SRAP*||66044672_UmuD->66044673_UmuC-><-66044674_?       66043995     ClpB-><-?<-SRAP*||UmuD->UmuC->                                             -                               147   bacteria>proteobacteria>gammaproteobacteria    Pseudomonas syringae pv. syringae B728a                                      hypothetical protein Psyr_0734 [Pseudomonas syringae pv. syringae B728a].                                                      66043992_?->66043993_ClpB-><-66043994_?<-66043995_SRAP*||66043996_UmuD->66043997_UmuC-><-66043998_?       257486997    <-UmuC<-UmuC<-UmuD||SRAP*->?->SRAP->                                       PsyrptA_020100027230            230   bacteria>proteobacteria>gammaproteobacteria    Pseudomonas syringae pv. tabaci str. ATCC 11528                              hypothetical protein PsyrptA_27230 [Pseudomonas syringae pv. tabaci str. ATCC 11528].                                          <-257486994_UmuC<-257486995_UmuC<-257486996_UmuD||257486997_SRAP*->257486998_?->257486999_SRAP-><-257487000_?       213971545    <-UmuC<-UmuD||SRAP*->                                                      PSPTOT1_0857                    232   bacteria>proteobacteria>gammaproteobacteria    Pseudomonas syringae pv. tomato T1                                           hypothetical protein PSPTOT1_0857 [Pseudomonas syringae pv. tomato T1].                                                        213971542_?-><-213971543_UmuC<-213971544_UmuD||213971545_SRAP*->213971546_?-><-213971547_?<-213971548_?       213972205    <-SRAP*||UmuD->UmuC->                                                      PSPTOT1_3120                    230   bacteria>proteobacteria>gammaproteobacteria    Pseudomonas syringae pv. tomato T1                                           hypothetical protein PSPTOT1_3120 [Pseudomonas syringae pv. tomato T1].                                                        213972204_?-><-213972205_SRAP*||213972206_UmuD->213972207_UmuC->213972208_?->       410090249    <-SRAP*||UmuD->UmuC->                                                      AAI_06343                       229   bacteria>proteobacteria>gammaproteobacteria    Pseudomonas viridiflava UASWS0038                                            hypothetical protein AAI_06343 [Pseudomonas viridiflava UASWS0038].                                                            <-410090249_SRAP*||410090250_UmuD->410090251_UmuC-><-410090252_?       384257654    UmuD->UmuC-><-SRAP*<-SRAP                                                  -                               160   bacteria>proteobacteria>gammaproteobacteria    Rahnella aquatilis HX2                                                       hypothetical protein Q7S_08915 [Rahnella aquatilis HX2].                                                                       384257651_?->384257652_UmuD->384257653_UmuC-><-384257654_SRAP*<-384257655_SRAP<-384257656_?<-384257657_?<-384257658_?       469818150    <-ImuB+ImuB-C<-SulA<-UmuD<-SRAP*                                           -                               224   bacteria>proteobacteria>gammaproteobacteria    Rhodanobacter sp. 2APBS1                                                     hypothetical protein R2APBS1_1875 [Rhodanobacter sp. 2APBS1].                                                                  <-469818147_ImuB+ImuB-C<-469818148_SulA<-469818149_UmuD<-469818150_SRAP*<-469818151_?<-469818152_?<-469818153_?       339999443    SRAP*-><-UmuC<-UmuD                                                        -                               223   bacteria>proteobacteria>gammaproteobacteria    Salmonella bongori NCTC 12419                                                hypothetical protein SBG_1461 [Salmonella bongori NCTC 12419].                                                                 <-339999440_?<-339999441_?||339999442_?->339999443_SRAP*-><-339999444_UmuC<-339999445_UmuD<-339999446_?       452119512    <-UmuD<-SRAP*                                                              CFSAN001992_00025               223   bacteria>proteobacteria>gammaproteobacteria    Salmonella enterica subsp. enterica serovar Javiana str. CFSAN001992         hypothetical protein CFSAN001992_00025 [Salmonella enterica subsp. enterica serovar Javiana str. CFSAN001992].                 <-452119510_?<-452119511_UmuD<-452119512_SRAP*||452119513_?->452119514_?->452119515_?->       458380968    SRAP*-><-UmuC<-UmuD                                                        B389_RS03840                    223   bacteria>proteobacteria>gammaproteobacteria    Salmonella enterica subsp. enterica serovar Mississippi str. 2010K-1406      hypothetical protein B389_RS03840 [Salmonella enterica subsp. enterica serovar Mississippi str. 2010K-1406].                   <-458380965_?||458380966_?->458380967_?->458380968_SRAP*-><-458380969_UmuC<-458380970_UmuD<-458380971_?       418805154    <-DAM||SRAP*->UmuD->UmuC->                                                 SEEN202_20747                   223   bacteria>proteobacteria>gammaproteobacteria    Salmonella enterica subsp. enterica serovar Newport str. CVM 35202           hypothetical protein SEEN202_20747 [Salmonella enterica subsp. enterica serovar Newport str. CVM 35202].                       <-418805151_?<-418805152_?<-418805153_DAM||418805154_SRAP*->418805155_UmuD->418805156_UmuC-><-418805157_?       167553750    SRAP*-><-UmuC<-UmuD                                                        SeSPB_A2094                     223   bacteria>proteobacteria>gammaproteobacteria    Salmonella enterica subsp. enterica serovar Saintpaul str. SARA29            conserved hypothetical protein [Salmonella enterica subsp. enterica serovar Saintpaul str. SARA29].                            <-167553746_?<-167553747_?||167553749_?->167553750_SRAP*-><-167553751_UmuC<-167553752_UmuD<-167553753_?       238912037    UmuD->UmuC-><-SRAP*<-?||?->NUDIX->                                         SentesTe_010100013026           223   bacteria>proteobacteria>gammaproteobacteria    Salmonella enterica subsp. enterica serovar Tennessee str. CDC07-0191        hypothetical protein SentesTe_13026 [Salmonella enterica subsp. enterica serovar Tennessee str. CDC07-0191].                   238912034_?->238912035_UmuD->238912036_UmuC-><-238912037_SRAP*<-238912038_?||238912039_?->238912040_NUDIX->       409248417    <-UmuD<-?<-?<-SRAP*<-?<-?||DAM->                                           SENTW_5552                      174   bacteria>proteobacteria>gammaproteobacteria    Salmonella enterica subsp. enterica serovar Weltevreden str. 2007-60-3289-1  Uncharacterized protein yedK [Salmonella enterica subsp. enterica serovar Weltevreden str. 2007-60-3289-1].                    <-409248414_UmuD<-409248415_?<-409248416_?<-409248417_SRAP*<-409248418_?<-409248419_?||409248420_DAM->       410690654    <-DAM||?->?->SRAP*-><-?||UmuD->UmuC->                                      D670_p1116                      243   bacteria>proteobacteria>gammaproteobacteria    Salmonella sp. 14                                                            Gifsy-2 prophage protein [Salmonella sp. 14].                                                                                  <-410690651_DAM||410690652_?->410690653_?->410690654_SRAP*-><-410690655_?||410690656_UmuD->410690657_UmuC->       270265395    <-UmuC<-?<-SRAP*                                                           SOD_n00150                      239   bacteria>proteobacteria>gammaproteobacteria    Serratia odorifera 4Rx13                                                     hypothetical protein SOD_n00150 [Serratia odorifera 4Rx13].                                                                    270265392_?-><-270265393_UmuC<-270265394_?<-270265395_SRAP*<-270265396_?<-270265397_?||270265398_?->       421780905    SRAP*->?->UmuD->UmuC->                                                     B194_5393                       228   bacteria>proteobacteria>gammaproteobacteria    Serratia plymuthica A30                                                      hypothetical protein B194_5393 [Serratia plymuthica A30].                                                                      421780902_?->421780903_?-><-421780904_?||421780905_SRAP*->421780906_?->421780907_UmuD->421780908_UmuC->       333926836    UmuD-><-?||?-><-SRAP*                                                      -                               95    bacteria>proteobacteria>gammaproteobacteria    Serratia sp. AS12                                                            hypothetical protein SerAS12_1980 [Serratia sp. AS12].                                                                         333926833_UmuD-><-333926834_?||333926835_?-><-333926836_SRAP*||333926837_?->333926838_?-><-333926839_?       418025787    <-UmuC<-UmuD<-?||SRAP*->                                                   Sbal625DRAFT_3891               189   bacteria>proteobacteria>gammaproteobacteria    Shewanella baltica OS625                                                     protein of unknown function DUF159 [Shewanella baltica OS625].                                                                 <-418025784_UmuC<-418025785_UmuD<-418025786_?||418025787_SRAP*-><-418025788_?<-418025789_?<-418025790_?       91794582     SRAP*-><-UmuC<-UmuD                                                        -                               190   bacteria>proteobacteria>gammaproteobacteria    Shewanella denitrificans OS217                                               hypothetical protein Sden_3234 [Shewanella denitrificans OS217].                                                               <-91794579_?<-91794580_?||91794581_?->91794582_SRAP*-><-91794583_UmuC<-91794584_UmuD<-91794585_?       424667637    <-UmuC<-UmuD||?->SRAP*->                                                   -                               315   bacteria>proteobacteria>gammaproteobacteria    Stenotrophomonas maltophilia Ab55555                                         hypothetical protein A1OC_01215 [Stenotrophomonas maltophilia Ab55555].                                                        <-424667634_UmuC<-424667635_UmuD||424667636_?->424667637_SRAP*->424667638_?-><-424667639_?||424667640_?->       254524336    SRAP*->?-><-UmuC<-UmuD                                                     SSKA14_3474                     310   bacteria>proteobacteria>gammaproteobacteria    Stenotrophomonas sp. SKA14                                                   conserved hypothetical protein [Stenotrophomonas sp. SKA14].                                                                   254523366_?->254524653_?-><-254523276_?||254524336_SRAP*->254523179_?-><-254521829_UmuC<-254521402_UmuD       344339221    SRAP*-><-UmuD<-?<-UmuC                                                     ThimaDRAFT_1889                 230   bacteria>proteobacteria>gammaproteobacteria    Thiocapsa marina 5811                                                        protein of unknown function DUF159 [Thiocapsa marina 5811].                                                                    344339218_?->344339219_?-><-344339220_?||344339221_SRAP*-><-344339222_UmuD<-344339223_?<-344339224_UmuC       381156877    <-SRAP*<-RecD<-UmuD                                                        Thi970DRAFT_00465               238   bacteria>proteobacteria>gammaproteobacteria    Thiorhodovibrio sp. 970                                                      hypothetical protein Thi970DRAFT_00465 [Thiorhodovibrio sp. 970].                                                              <-381156874_?<-381156875_?<-381156876_?<-381156877_SRAP*<-381156878_RecD<-381156879_UmuD<-381156880_?       470044600    <-SRAP*<-UmuC<-UmuD                                                        LEP1GSC172_4315                 237   bacteria>spirochaetes                          Leptospira interrogans str. HAI1536                                          hypothetical protein LEP1GSC172_4315 [Leptospira interrogans str. HAI1536].                                                    <-470044593_?<-470044663_?<-470044576_?<-470044600_SRAP*<-470044651_UmuC<-470044647_UmuD||470044659_?->       470028681    <-SRAP*<-UmuC<-UmuD                                                        LEP1GSC186_3291                 237   bacteria>spirochaetes                          Leptospira noguchii serovar Autumnalis str. ZUN142                           hypothetical protein LEP1GSC186_3291 [Leptospira noguchii serovar Autumnalis str. ZUN142].                                     <-470028663_?<-470028720_?<-470028692_?<-470028681_SRAP*<-470028696_UmuC<-470028690_UmuD||470028703_?->       469870369    <-SRAP*<-UmuC<-UmuD||SRAP->                                                LEP1GSC084_0019                 177   bacteria>spirochaetes                          Leptospira interrogans serovar Medanensis str. L0448                         hypothetical protein LEP1GSC084_0019 [Leptospira interrogans serovar Medanensis str. L0448].                                   <-469870369_SRAP*<-469870368_UmuC<-469870367_UmuD||469870366_SRAP->       418670631    <-SRAP*<-UmuC<-UmuD                                                        LEP1GSC019_1656                 237   bacteria>spirochaetes                          Leptospira interrogans serovar Pyrogenes str. 2006006960                     hypothetical protein LEP1GSC019_1656 [Leptospira interrogans serovar Pyrogenes str. 2006006960].                               <-418670630_?||418670638_?-><-418670623_?<-418670631_SRAP*<-418670632_UmuC<-418670639_UmuD||418670626_?->       410942113    <-SRAP*<-UmuC<-UmuD                                                        LEP1GSC041_0608                 158   bacteria>spirochaetes                          Leptospira noguchii str. 2006001870                                          hypothetical protein LEP1GSC041_0608 [Leptospira noguchii str. 2006001870].                                                    <-410942064_?<-410942038_?<-410942069_?<-410942113_SRAP*<-410942119_UmuC<-410942087_UmuD||410942080_?->       398348608    SRAP*->UmuD->UmuC->                                                        Lbro5_010100015529              237   bacteria>spirochaetes                          Leptospira broomii serovar Hurstbridge str. 5399                             hypothetical protein Lbro5_15529 [Leptospira broomii serovar Hurstbridge str. 5399].                                           398348605_?-><-398348606_?<-398348607_?||398348608_SRAP*->398348609_UmuD->398348610_UmuC-><-398348611_?       398334214    SRAP*->UmuD->UmuC->                                                        LkmesMB_010100000300            241   bacteria>spirochaetes                          Leptospira kmetyi serovar Malaysia str. Bejo-Iso9                            hypothetical protein LkmesMB_00300 [Leptospira kmetyi serovar Malaysia str. Bejo-Iso9].                                        <-398334211_?<-398334212_?<-398334213_?||398334214_SRAP*->398334215_UmuD->398334216_UmuC-><-398334217_?       470063573    <-SRAP*<-UmuC<-UmuD                                                        LEP1GSC132_0009                 237   bacteria>spirochaetes                          Leptospira kirschneri str. 200803703                                         hypothetical protein LEP1GSC132_0009 [Leptospira kirschneri str. 200803703].                                                   <-470063577_?<-470063568_?<-470063571_?<-470063573_SRAP*<-470063578_UmuC<-470063576_UmuD||470063575_?->       189220308    SRAP*->UmuD->UmuC->                                                        Minf_2297                       232   bacteria>verrucomicrobia                       Methylacidiphilum infernorum V4                                              hypothetical protein Minf_2297 [Methylacidiphilum infernorum V4].                                                              189220305_?->189220306_?->189220307_?->189220308_SRAP*->189220309_UmuD->189220310_UmuC-><-189220311_?       126642059    SRAP*->UmuC->                                                              -                               216   bacteria>proteobacteria>gammaproteobacteria    Acinetobacter baumannii ATCC 17978                                           hypothetical protein A1S_2014 [Acinetobacter baumannii ATCC 17978].                                                            126642056_?-><-126642057_?<-126642058_?||126642059_SRAP*->126642060_UmuC-><-126642061_?<-126642062_?       146311861    SRAP*->SRAP-><-?<-UmuC                                                     -                               85    bacteria>proteobacteria>gammaproteobacteria    Enterobacter sp. 638                                                         hypothetical protein Ent638_2209 [Enterobacter sp. 638].                                                                       146311858_?->146311859_?->146311860_?->146311861_SRAP*->146311862_SRAP-><-146311863_?<-146311864_UmuC       167992478    SRAP*-><-UmuC                                                              SeI_A1030                       223   bacteria>proteobacteria>gammaproteobacteria    Salmonella enterica subsp. enterica serovar 4,[5],12:i:- str. CVM23701       conserved hypothetical protein [Salmonella enterica subsp. enterica serovar 4,[5],12:i:- str. CVM23701].                       167992474_?->167992475_?->167992476_?->167992478_SRAP*-><-205357825_UmuC||167992480_?-><-167992481_?       251797724    <-UmuC<-?||SRAP*-><-?<-RecG                                                -                               232   bacteria>firmicutes                            Paenibacillus sp. JDR-2                                                      hypothetical protein Pjdr2_3739 [Paenibacillus sp. JDR-2].                                                                     <-251797721_?<-251797722_UmuC<-251797723_?||251797724_SRAP*-><-251797725_?<-251797726_RecG<-251797727_?       257868358    <-SRAP*<-?<-UmuC                                                           EGAG_03229                      196   bacteria>firmicutes                            Enterococcus casseliflavus EC30                                              conserved hypothetical protein [Enterococcus casseliflavus EC30].                                                              257868355_?->257868356_?->257868357_?-><-257868358_SRAP*<-257868359_?<-257868360_UmuC<-257868361_?       288934698    UmuC-><-SRAP*                                                              -                               223   bacteria>proteobacteria>gammaproteobacteria    Klebsiella variicola At-22                                                   hypothetical protein Kvar_1824 [Klebsiella variicola At-22].                                                                   288934695_?->288934696_?->288934697_UmuC-><-288934698_SRAP*<-288934699_?<-288934700_?||288934701_?->       295425709    SRAP*-><-?<-?<-UmuC                                                        HMPREF0493_0837                 198   bacteria>firmicutes                            Lactobacillus amylolyticus DSM 11664                                         conserved hypothetical protein [Lactobacillus amylolyticus DSM 11664].                                                         <-295425706_?<-295425707_?<-295425708_?||295425709_SRAP*-><-295425710_?<-295425711_?<-295425712_UmuC       304320275    <-UmuC||SRAP*->                                                            PB2503_03507                    134   bacteria>proteobacteria>alphaproteobacteria    Parvularcula bermudensis HTCC2503                                            hypothetical protein PB2503_03507 [Parvularcula bermudensis HTCC2503].                                                         304320272_?-><-304320273_?<-304320274_UmuC||304320275_SRAP*->304320276_?-><-304320277_?||304320278_?->       326387350    <-UmuC||?->SRAP*->                                                         -                               208   bacteria>proteobacteria>alphaproteobacteria    Novosphingobium nitrogenifigens DSM 19370                                    hypothetical protein Y88_1400 [Novosphingobium nitrogenifigens DSM 19370].                                                     <-326387353_?<-326387352_UmuC||326387351_?->326387350_SRAP*->326387349_?->326387348_?->326387347_?->       336250894    SRAP*->?->?-><-UmuC                                                        -                               66    bacteria>proteobacteria>gammaproteobacteria    Enterobacter aerogenes KCTC 2190                                             hypothetical protein EAE_22130 [Enterobacter aerogenes KCTC 2190].                                                             336250891_?->336250892_?->336250893_?->336250894_SRAP*->336250895_?->336250896_?-><-336250897_UmuC       354582490    RecG->?-><-SRAP*||?->?->UmuC->                                             PaelaDRAFT_2493                 236   bacteria>firmicutes                            Paenibacillus lactis 154                                                     protein of unknown function DUF159 [Paenibacillus lactis 154].                                                                 354582487_?->354582488_RecG->354582489_?-><-354582490_SRAP*||354582491_?->354582492_?->354582493_UmuC->       374602063    <-SRAP*||?-><-?<-UmuC                                                      PDENDC454_03914                 227   bacteria>firmicutes                            Paenibacillus dendritiformis C454                                            hypothetical protein PDENDC454_03914 [Paenibacillus dendritiformis C454].                                                      374602060_?->374602061_?->374602062_?-><-374602063_SRAP*||374602064_?-><-374602065_?<-374602066_UmuC       381201468    <-SRAP*<-?<-UmuC                                                           SyanX_010100013278              210   bacteria>proteobacteria>alphaproteobacteria    Sphingobium yanoikuyae XLDN2-5                                               hypothetical protein SyanX_13278 [Sphingobium yanoikuyae XLDN2-5].                                                             381201465_?->381201466_?->381201467_?-><-381201468_SRAP*<-381201469_?<-381201470_UmuC       419958497    UmuC->?-><-SRAP*                                                           PGS1_12001                      223   bacteria>proteobacteria>gammaproteobacteria    Enterobacter cloacae subsp. cloacae GS1                                      hypothetical protein PGS1_12001 [Enterobacter cloacae subsp. cloacae GS1].                                                     419958494_?->419958495_UmuC->419958496_?-><-419958497_SRAP*<-419958498_?||419958499_?-><-419958500_?       422684213    <-UmuC<-UmuD||SRAP*->                                                      PSYTB_28592                     75    bacteria>proteobacteria>gammaproteobacteria    Pseudomonas syringae pv. tabaci str. ATCC 11528                              hypothetical protein PSYTB_28592, partial [Pseudomonas syringae pv. tabaci str. ATCC 11528].                                   422684210_?-><-422684211_UmuC<-422684212_UmuD||422684213_SRAP*->       423123604    UmuC-><-?<-SRAP*                                                           -                               225   bacteria>proteobacteria>gammaproteobacteria    Klebsiella oxytoca 10-5250                                                   hypothetical protein HMPREF9694_00295 [Klebsiella oxytoca 10-5250].                                                            <-423123601_?||423123602_UmuC-><-423123603_?<-423123604_SRAP*||423123605_?-><-423123606_?<-423123607_?       458964721    UmuC-><-SRAP*                                                              SASAL_RS19515                   138   bacteria>proteobacteria>gammaproteobacteria    Salmonella enterica subsp. salamae str. 3588/07                              hypothetical protein SASAL_RS19515, partial [Salmonella enterica subsp. salamae str. 3588/07].                                 <-458964718_?<-458964719_?||458964720_UmuC-><-458964721_SRAP*       479190884    UmuC->UmuD->?->SRAP*->                                                     -                               207   bacteria>firmicutes                            Butyrivibrio fibrisolvens 16/4                                               Uncharacterized conserved protein [Butyrivibrio fibrisolvens 16/4].                                                            479190881_UmuC->479190882_UmuD->479190883_?->479190884_SRAP*-><-479190885_?<-479190886_?<-479190887_?       430750378    <-YolD<-UmuC||SRAP*-><-?<-RecG                                             -                               226   bacteria>firmicutes                            Thermobacillus composti KWC4                                                 hypothetical protein Theco_2167 [Thermobacillus composti KWC4].                                                                <-430750375_?<-430750376_YolD<-430750377_UmuC||430750378_SRAP*-><-430750379_?<-430750380_RecG<-430750381_?       458383039    SRAP*-><-UmuC<-SRAP                                                        B389_RS14720                    223   bacteria>proteobacteria>gammaproteobacteria    Salmonella enterica subsp. enterica serovar Mississippi str. 2010K-1406      hypothetical protein B389_RS14720 [Salmonella enterica subsp. enterica serovar Mississippi str. 2010K-1406].                   <-458383036_?||458383037_?->458383038_?->458383039_SRAP*-><-458383040_UmuC<-458383041_SRAP       479872022    <-SRAP*<-?||UmuC->                                                         F993_01642                      220   bacteria>proteobacteria>gammaproteobacteria    Acinetobacter sp. NIPH 809                                                   hypothetical protein F993_01642 [Acinetobacter sp. NIPH 809].                                                                  <-479872019_?||479872020_?->479872021_?-><-479872022_SRAP*<-479872023_?||479872024_UmuC->479872025_?->       479954842    <-UmuC<-UmuD<-SRAP*                                                        F969_00742                      112   bacteria>proteobacteria>gammaproteobacteria    Acinetobacter sp. NIPH 899                                                   hypothetical protein F969_00742 [Acinetobacter sp. NIPH 899].                                                                  <-479954839_?<-479954840_UmuC<-479954841_UmuD<-479954842_SRAP*<-479954843_?<-479954844_?||479954845_?->       479977072    SRAP*->UmuC->                                                              F963_03183                      214   bacteria>proteobacteria>gammaproteobacteria    Acinetobacter bereziniae NIPH 3                                              hypothetical protein F963_03183 [Acinetobacter bereziniae NIPH 3].                                                             479977069_?->479977070_?->479977071_?->479977072_SRAP*->479977073_UmuC->479977074_?->479977075_?->       480338091    <-SRAP*<-?||UmuC->                                                         F902_02696                      220   bacteria>proteobacteria>gammaproteobacteria    Acinetobacter sp. CIP 70.18                                                  hypothetical protein F902_02696 [Acinetobacter sp. CIP 70.18].                                                                 <-480338088_?||480338089_?->480338090_?-><-480338091_SRAP*<-480338092_?||480338093_UmuC->480338094_?->       480476667    SRAP*-><-UmuC                                                              RORB6_09995                     223   bacteria>proteobacteria>gammaproteobacteria    Raoultella ornithinolytica B6                                                hypothetical protein RORB6_09995 [Raoultella ornithinolytica B6].                                                              480476664_?->480476665_?-><-480476666_?||480476667_SRAP*-><-480476668_UmuC<-480476669_?||480476670_?->       9187583      <-UmuC<-UmuD||SRAP*->                                                      -                               283   bacteria>proteobacteria>gammaproteobacteria    Pseudomonas syringae pv. pisi                                                hypothetical protein [Pseudomonas syringae pv. pisi].                                                                          <-9187580_?<-9187581_UmuC<-9187582_UmuD||9187583_SRAP*->       70729402     <-SRAP*||UmuC->                                                            -                               227   bacteria>proteobacteria>gammaproteobacteria    Pseudomonas protegens Pf-5                                                   hypothetical protein PFL_2021 [Pseudomonas protegens Pf-5].                                                                    70729399_?->70729400_?->70729401_?-><-70729402_SRAP*||70729403_UmuC-><-70729404_?<-70729405_?       9187583      <-UmuC<-UmuD||SRAP*->                                                      -                               283   bacteria>proteobacteria>gammaproteobacteria    Pseudomonas syringae pv. pisi                                                hypothetical protein [Pseudomonas syringae pv. pisi].                                                                          <-9187580_?<-9187581_UmuC<-9187582_UmuD||9187583_SRAP*->        # Variant DnaE2-associated       89889520     <-LexA||SRAP*->?->inac_DinB->DnaE2->                                       -                               275   bacteria>bacteroidetes                         Flavobacteria bacterium BBFL7                                                conserved hypothetical protein [Flavobacteria bacterium BBFL7].                                                                89889517_?-><-89889518_?<-89889519_LexA||89889520_SRAP*->89889521_?->89889522_inac_DinB->89889523_DnaE2->        # ImuAB-DnaE2 associated       407001119    DnaE2->?->?->SRAP*->                                                       ACD_10C00087G0005               226   bacteria                                       uncultured bacterium                                                         hypothetical protein ACD_10C00087G0005 [uncultured bacterium].                                                                 407001116_DnaE2->407001117_?->407001118_?->407001119_SRAP*->       374312068    <-SRAP*||RecA->ImuB+ImuB-C->DnaE2->                                        -                               214   bacteria>acidobacteria                         Granulicella mallensis MP5ACTX8                                              hypothetical protein [Granulicella mallensis MP5ACTX8].                                                                        <-374312065_?<-374312066_?||374312067_?-><-374312068_SRAP*||374312069_RecA->374312070_ImuB+ImuB-C->374312071_DnaE2->       225873139    <-SRAP*||RecA->ImuB+ImuB-C->DnaE2->                                        -                               211   bacteria>acidobacteria                         Acidobacterium capsulatum ATCC 51196                                         hypothetical protein ACP_1516 [Acidobacterium capsulatum ATCC 51196].                                                          225873136_?-><-225873137_?||225873138_?-><-225873139_SRAP*||225873140_RecA->225873141_ImuB+ImuB-C->225873142_DnaE2->       225874746    <-DnaE2<-ImuB+ImuB-C<-RecA||SRAP*->                                        -                               212   bacteria>acidobacteria                         Acidobacterium capsulatum ATCC 51196                                         hypothetical protein ACP_3200 [Acidobacterium capsulatum ATCC 51196].                                                          <-225874743_DnaE2<-225874744_ImuB+ImuB-C<-225874745_RecA||225874746_SRAP*->225874747_?->225874748_?-><-225874749_?       403251007    SRAP*->?->DnaE2->                                                          A27L6_005800000020              242   bacteria>actinobacteria                        actinobacterium SCGC AAA027-L06                                              hypothetical protein A27L6_005800000020 [actinobacterium SCGC AAA027-L06].                                                     403251006_?->403251007_SRAP*->403251008_?->403251009_DnaE2->       255036111    DnaE2->?->?->SRAP*->                                                       -                               274   bacteria>bacteroidetes                         Dyadobacter fermentans DSM 18053                                             hypothetical protein Dfer_2348 [Dyadobacter fermentans DSM 18053].                                                             255036108_DnaE2->255036109_?->255036110_?->255036111_SRAP*-><-255036112_?<-255036113_?<-255036114_?       409097669    <-ImuA<-?<-?<-SRAP*                                                        PagrP_010100004404              273   bacteria>bacteroidetes                         Pedobacter agri PB92                                                         hypothetical protein PagrP_04404 [Pedobacter agri PB92].                                                                       <-409097666_ImuA<-409097667_?<-409097668_?<-409097669_SRAP*<-409097670_?<-409097671_?||409097672_?->       479338688    SRAP*->ImuB-C->                                                            -                               272   bacteria>firmicutes                            Clostridium cf. saccharolyticum K10                                          Uncharacterized conserved protein [Clostridium cf. saccharolyticum K10].                                                       <-479338685_?<-479338686_?||479338687_?->479338688_SRAP*->479338689_ImuB-C-><-479338690_?||479338691_?->       296121583    <-DnaE2<-ImuB+ImuB-C<-ImuA||SRAP*->                                        Plim_1328                       224   bacteria>planctomycetes                        Planctomyces limnophilus DSM 3776                                            hypothetical protein Plim_1328 [Planctomyces limnophilus DSM 3776].                                                            <-296121580_DnaE2<-296121581_ImuB+ImuB-C<-296121582_ImuA||296121583_SRAP*-><-296121584_?<-296121585_?<-296121586_?       87307674     <-DnaE2<-ImuB+ImuB-C<-ImuA||SRAP*->                                        -                               227   bacteria>planctomycetes                        Blastopirellula marina DSM 3645                                              hypothetical protein DSM3645_29172 [Blastopirellula marina DSM 3645].                                                          <-87307671_DnaE2<-87307672_ImuB+ImuB-C<-87307673_ImuA||87307674_SRAP*->87307675_?->87307676_?->87307677_?->       148243811    <-DnaE2<-ImuB+ImuB-C||UvrD->SRAP*->                                        Acry_3296                       244   bacteria>proteobacteria>alphaproteobacteria    Acidiphilium cryptum JF-5                                                    hypothetical protein Acry_3296 [Acidiphilium cryptum JF-5].                                                                    <-148243808_DnaE2<-148243809_ImuB+ImuB-C||148243810_UvrD->148243811_SRAP*-><-148243812_?||148243813_?->148243814_?->       16119325     <-SRAP*<-ATP-dependent-ligase||ImuA->ImuB+ImuB-C->                         Atu5096                         257   bacteria>proteobacteria>alphaproteobacteria    Agrobacterium fabrum str. C58                                                hypothetical protein Atu5096 [Agrobacterium fabrum str. C58].                                                                  <-16119322_?<-16119323_?||16119324_?-><-16119325_SRAP*<-16119326_ATP-dependent-ligase||16119327_ImuA->16119328_ImuB+ImuB-C->       335033132    ImuA->ImuB+ImuB-C->DnaE2->SRAP->SRAP*->                                    AGRO_0473                       214   bacteria>proteobacteria>alphaproteobacteria    Agrobacterium sp. ATCC 31749                                                 hypothetical protein AGRO_0473 [Agrobacterium sp. ATCC 31749].                                                                 335033128_ImuA->335033129_ImuB+ImuB-C->335033130_DnaE2->335033131_SRAP->335033132_SRAP*-><-335033133_?||335033134_?->335033135_?->       325168582    <-SRAP*<-ATP-dependent-ligase||ImuA->ImuB+ImuB-C->                         AGROH133_14624                  254   bacteria>proteobacteria>alphaproteobacteria    Agrobacterium sp. H13-3                                                      hypothetical protein [Agrobacterium sp. H13-3].                                                                                325168579_?->325168580_?->325168581_?-><-325168582_SRAP*<-325168583_ATP-dependent-ligase||325168584_ImuA->325168585_ImuB+ImuB-C->       424725319    <-DinB||?->?-><-SRAP*<-ATP-dependent-ligase||ImuA->ImuB+ImuB-C->           F500_p025                       257   bacteria>proteobacteria>alphaproteobacteria    Agrobacterium tumefaciens                                                    Hypothetical protein [Agrobacterium tumefaciens].                                                                              <-424725316_DinB||424725317_?->424725318_?-><-424725319_SRAP*<-424725320_ATP-dependent-ligase||424725321_ImuA->424725322_ImuB+ImuB-C->       418409665    <-ImuB+ImuB-C<-ImuA||ATP-dependent-ligase->SRAP*->                         AT5A_20651                      254   bacteria>proteobacteria>alphaproteobacteria    Agrobacterium tumefaciens 5A                                                 hypothetical protein AT5A_20651 [Agrobacterium tumefaciens 5A].                                                                <-418409662_ImuB+ImuB-C<-418409663_ImuA||418409664_ATP-dependent-ligase->418409665_SRAP*-><-418409666_?<-418409667_?||418409668_?->       421597929    DnaE2->SRAP*->                                                             BCCGELA001_10772                251   bacteria>proteobacteria>alphaproteobacteria    Bradyrhizobium sp. CCGE-LA001                                                hypothetical protein BCCGELA001_10772 [Bradyrhizobium sp. CCGE-LA001].                                                         <-421597926_?<-421597927_?||421597928_DnaE2->421597929_SRAP*->       167621853    <-DnaE2<-ImuB+ImuB-C<-ImuA||SRAP*->                                        Caul_5204                       257   bacteria>proteobacteria>alphaproteobacteria    Caulobacter sp. K31                                                          hypothetical protein Caul_5204 [Caulobacter sp. K31].                                                                          <-167621850_DnaE2<-167621851_ImuB+ImuB-C<-167621852_ImuA||167621853_SRAP*-><-167621854_?<-167621855_?<-167621856_?       110636136    DnaE2->DinB->?-><-SRAP*                                                    -                               199   bacteria>proteobacteria>alphaproteobacteria    Chelativorans sp. BNC1                                                       hypothetical protein Meso_3812 [Chelativorans sp. BNC1].                                                                       110636133_DnaE2->110636134_DinB->110636135_?-><-110636136_SRAP*||110636137_?->110636138_?->110636139_?->       159046533    SRAP*->ImuA->ImuB+ImuB-C->DnaE2->                                          Dshi_3995                       226   bacteria>proteobacteria>alphaproteobacteria    Dinoroseobacter shibae DFL 12                                                hypothetical protein Dshi_3995 [Dinoroseobacter shibae DFL 12].                                                                <-159046530_?<-159046531_?<-159046532_?||159046533_SRAP*->159046534_ImuA->159046535_ImuB+ImuB-C->159046536_DnaE2->       162145935    <-SRAP*<-?||?->ImuA->                                                      -                               219   bacteria>proteobacteria>alphaproteobacteria    Gluconacetobacter diazotrophicus PAl 5                                       hypothetical protein GDI_0099 [Gluconacetobacter diazotrophicus PAl 5].                                                        162145932_?->162145933_?->162145934_?-><-162145935_SRAP*<-162145936_?||162145937_?->162145938_ImuA->       340779643    <-SRAP*||ImuA->                                                            AaceN1_010100017428             215   bacteria>proteobacteria>alphaproteobacteria    Acetobacter aceti NBRC 14818                                                 hypothetical protein AaceN1_17428 [Acetobacter aceti NBRC 14818].                                                              <-340779643_SRAP*||340779644_ImuA->       349701738    <-ImuA||SRAP*->                                                            GeurL1_010100013149             216   bacteria>proteobacteria>alphaproteobacteria    Gluconacetobacter europaeus LMG 18494                                        hypothetical protein GeurL1_13149 [Gluconacetobacter europaeus LMG 18494].                                                     <-349701736_?<-349701737_ImuA||349701738_SRAP*-><-349701739_?<-349701740_?<-349701741_?       296115457    <-ImuB+ImuB-C<-ImuA||UvrD->SRAP*->                                         -                               215   bacteria>proteobacteria>alphaproteobacteria    Gluconacetobacter hansenii ATCC 23769                                        hypothetical protein GXY_06730 [Gluconacetobacter hansenii ATCC 23769].                                                        <-296115454_ImuB+ImuB-C<-296115455_ImuA||296115456_UvrD->296115457_SRAP*-><-296115458_?||296115459_?->296115460_?->       296114711    <-ImuA||?->?->SRAP*->                                                      -                               215   bacteria>proteobacteria>alphaproteobacteria    Gluconacetobacter hansenii ATCC 23769                                        hypothetical protein GXY_03003 [Gluconacetobacter hansenii ATCC 23769].                                                        <-296114708_ImuA||296114709_?->296114710_?->296114711_SRAP*-><-296114712_?<-296114713_?<-296114714_?       349689327    <-ImuB+ImuB-C<-ImuA||SRAP*->                                               Gobo1_010100019206              216   bacteria>proteobacteria>alphaproteobacteria    Gluconacetobacter oboediens 174Bp2                                           hypothetical protein Gobo1_19206 [Gluconacetobacter oboediens 174Bp2].                                                         <-349689325_ImuB+ImuB-C<-349689326_ImuA||349689327_SRAP*-><-349689328_?||349689329_?->349689330_?->       330990451    <-ImuB+ImuB-C<-ImuA<-?||SRAP*-><-?||?-><-DnaE2                             SXCC_00363                      217   bacteria>proteobacteria>alphaproteobacteria    Gluconacetobacter sp. SXCC-1                                                 hypothetical protein SXCC_00363 [Gluconacetobacter sp. SXCC-1].                                                                <-330990448_ImuB+ImuB-C<-330990449_ImuA<-330990450_?||330990451_SRAP*-><-330990452_?||330990453_?-><-330990454_DnaE2       347822854    <-DnaE2<-ImuB+ImuB-C<-ImuA||SRAP*->                                        GLX_29730                       169   bacteria>proteobacteria>alphaproteobacteria    Gluconacetobacter xylinus NBRC 3288                                          hypothetical protein GLX_29730 [Gluconacetobacter xylinus NBRC 3288].                                                          <-347822851_DnaE2<-347822852_ImuB+ImuB-C<-347822853_ImuA||347822854_SRAP*->347822855_?->347822856_?->347822857_?->       410945405    <-ImuB+ImuB-C<-ImuA<-?||SRAP*-><-?<-?||SRAP->                              GfraN1_010100013211             208   bacteria>proteobacteria>alphaproteobacteria    Gluconobacter frateurii NBRC 101659                                          hypothetical protein GfraN1_13211 [Gluconobacter frateurii NBRC 101659].                                                       <-410945402_ImuB+ImuB-C<-410945403_ImuA<-410945404_?||410945405_SRAP*-><-410945406_?<-410945407_?||410945408_SRAP->       389691870    <-DnaE2||?-><-SRAP*||?-><-SRAP                                             MicloDRAFT_00026820             225   bacteria>proteobacteria>alphaproteobacteria    Microvirga sp. WSM3557                                                       hypothetical protein MicloDRAFT_00026820 [Microvirga sp. WSM3557].                                                             <-389691867_?<-389691868_DnaE2||389691869_?-><-389691870_SRAP*||389691871_?-><-389691872_SRAP       92119224     <-ImuA||RecD->Transposase->SRAP*->                                         -                               151   bacteria>proteobacteria>alphaproteobacteria    Nitrobacter hamburgensis X14                                                 hypothetical protein Nham_3770 [Nitrobacter hamburgensis X14].                                                                 <-92119221_ImuA||92119222_RecD->92119223_Transposase->92119224_SRAP*-><-92119225_?<-92119226_?||92119227_?->       393772356    <-ImuA<-ImuA<-?<-SRAP*                                                     WSK_1789                        194   bacteria>proteobacteria>alphaproteobacteria    Novosphingobium sp. Rr 2-17                                                  hypothetical protein WSK_1789 [Novosphingobium sp. Rr 2-17].                                                                   <-393772353_ImuA<-393772354_ImuA<-393772355_?<-393772356_SRAP*<-393772357_?<-393772358_?<-393772359_?       163746769    <-SRAP*||ImuA->ImuB+ImuB-C->DnaE2->                                        OIHEL45_15239                   212   bacteria>proteobacteria>alphaproteobacteria    Oceanibulbus indolifex HEL-45                                                hypothetical protein OIHEL45_15239 [Oceanibulbus indolifex HEL-45].                                                            <-163746766_?||163746767_?->163746768_?-><-163746769_SRAP*||163746770_ImuA->163746771_ImuB+ImuB-C->163746772_DnaE2->       162148663    <-ImuB+ImuB-C<-ImuA||UvrD->SRAP*->                                         -                               213   bacteria>proteobacteria>alphaproteobacteria    Gluconacetobacter diazotrophicus PAl 5                                       hypothetical protein GDI_2890 [Gluconacetobacter diazotrophicus PAl 5].                                                        <-162148660_ImuB+ImuB-C<-162148661_ImuA||162148662_UvrD->162148663_SRAP*-><-162148664_?<-162148665_?<-162148666_?       444311981    <-DnaE2<-ImuB+ImuB-C<-ImuA||SRAP->SRAP*->                                  D584_19468                      215   bacteria>proteobacteria>alphaproteobacteria    Ochrobactrum intermedium M86                                                 hypothetical protein D584_19468 [Ochrobactrum intermedium M86].                                                                <-444311977_DnaE2<-444311978_ImuB+ImuB-C<-444311979_ImuA||444311980_SRAP->444311981_SRAP*-><-444311982_?<-444311983_?||444311984_?->       86360878     <-SRAP*||SRAP-><-DinB||ImuA->ImuB+ImuB-C->                                 RHE_PF00147                     263   bacteria>proteobacteria>alphaproteobacteria    Rhizobium etli CFN 42                                                        hypothetical protein RHE_PF00147 [Rhizobium etli CFN 42].                                                                      <-86360875_?||86360876_?->86360877_?-><-86360878_SRAP*||86360879_SRAP-><-86360880_DinB||86360881_ImuA->86360882_ImuB+ImuB-C->       190892915    <-DnaE2<-ImuB+ImuB-C<-ImuA||SRAP*->                                        -                               259   bacteria>proteobacteria>alphaproteobacteria    Rhizobium etli CIAT 652                                                      hypothetical protein RHECIAT_CH0003331 [Rhizobium etli CIAT 652].                                                              <-190892912_DnaE2<-190892913_ImuB+ImuB-C<-190892914_ImuA||190892915_SRAP*-><-190892916_?||190892917_?->190892918_?->       417111393    <-ImuB+ImuB-C<-ImuA||ATP-dependent-ligase->SRAP*->                         RHECNPAF_970010                 251   bacteria>proteobacteria>alphaproteobacteria    Rhizobium etli CNPAF512                                                      hypothetical protein RHECNPAF_970010 [Rhizobium etli CNPAF512].                                                                <-417111390_ImuB+ImuB-C<-417111391_ImuA||417111392_ATP-dependent-ligase->417111393_SRAP*-><-417111394_?||417111395_?-><-417111396_?       218462717    <-SRAP||SRAP*-><-DnaE2<-SRAP                                               RetlK5_010100026162             172   bacteria>proteobacteria>alphaproteobacteria    Rhizobium etli Kim 5                                                         hypothetical protein RetlK5_26162 [Rhizobium etli Kim 5].                                                                      218462715_?-><-218462716_SRAP||218462717_SRAP*-><-218462718_DnaE2<-218462719_SRAP       478271730    <-ImuB+ImuB-C<-ImuA||ATP-dependent-ligase->SRAP*->SRAP->                   RHSP_74457                      257   bacteria>proteobacteria>alphaproteobacteria    Rhizobium freirei PRF 81                                                     hypothetical protein RHSP_74457 [Rhizobium freirei PRF 81].                                                                    <-478271727_ImuB+ImuB-C<-478271728_ImuA||478271729_ATP-dependent-ligase->478271730_SRAP*->478271731_SRAP->478271732_?->478271733_?->478271734_?->       209547181    <-SRAP*||SRAP-><-DinB<-?||ImuA->                                           Rleg2_5153                      196   bacteria>proteobacteria>alphaproteobacteria    Rhizobium leguminosarum bv. trifolii WSM2304                                 hypothetical protein Rleg2_5153 [Rhizobium leguminosarum bv. trifolii WSM2304].                                                <-209547178_?||209547179_?->209547180_?-><-209547181_SRAP*||209547182_SRAP-><-209547183_DinB<-209547184_?||209547185_ImuA->       424917327    <-SRAP*||SRAP-><-DinB<-?||ImuA->                                           Rleg9DRAFT_4926                 196   bacteria>proteobacteria>alphaproteobacteria    Rhizobium leguminosarum bv. trifolii WSM597                                  hypothetical protein Rleg9DRAFT_4926 [Rhizobium leguminosarum bv. trifolii WSM597].                                            <-424917324_?||424917325_?->424917326_?-><-424917327_SRAP*||424917328_SRAP-><-424917329_DinB<-424917330_?||424917331_ImuA->       116254504    <-DinB<-SRAP*<-?||ImuA->                                                   pRL100040                       225   bacteria>proteobacteria>alphaproteobacteria    Rhizobium leguminosarum bv. viciae 3841                                      hypothetical protein pRL100040 [Rhizobium leguminosarum bv. viciae 3841].                                                      <-116254501_?||116254502_?-><-116254503_DinB<-116254504_SRAP*<-116254505_?||116254506_ImuA-><-116254507_?       424913073    <-SRAP*||ImuA->ImuB+ImuB-C->DnaE2->                                        Rleg13DRAFT_00334               257   bacteria>proteobacteria>alphaproteobacteria    Rhizobium leguminosarum bv. viciae USDA 2370                                 hypothetical protein Rleg13DRAFT_00334 [Rhizobium leguminosarum bv. viciae USDA 2370].                                         <-424913070_?||424913071_?-><-424913072_?<-424913073_SRAP*||424913074_ImuA->424913075_ImuB+ImuB-C->424913076_DnaE2->       424912830    <-SRAP*<-SRAP<-DnaE2<-ImuB+ImuB-C<-ImuA                                    Rleg13DRAFT_00073               214   bacteria>proteobacteria>alphaproteobacteria    Rhizobium leguminosarum bv. viciae USDA 2370                                 hypothetical protein Rleg13DRAFT_00073 [Rhizobium leguminosarum bv. viciae USDA 2370].                                         <-424912827_?<-424912828_?<-424912829_?<-424912830_SRAP*<-424912831_SRAP<-424912832_DnaE2<-424912833_ImuB+ImuB-C<-424912834_ImuA       424876884    <-SRAP*<-ATP-dependent-ligase||?->ImuA->                                   Rleg5DRAFT_1268                 257   bacteria>proteobacteria>alphaproteobacteria    Rhizobium leguminosarum bv. viciae WSM1455                                   hypothetical protein Rleg5DRAFT_1268 [Rhizobium leguminosarum bv. viciae WSM1455].                                             <-424876881_?||424876882_?->424876883_?-><-424876884_SRAP*<-424876885_ATP-dependent-ligase||424876886_?->424876887_ImuA->       424876815    <-SRAP*<-ATP-dependent-ligase||?->ImuA->                                   Rleg5DRAFT_1192                 257   bacteria>proteobacteria>alphaproteobacteria    Rhizobium leguminosarum bv. viciae WSM1455                                   hypothetical protein Rleg5DRAFT_1192 [Rhizobium leguminosarum bv. viciae WSM1455].                                             424876812_?-><-424876813_?||424876814_?-><-424876815_SRAP*<-424876816_ATP-dependent-ligase||424876817_?->424876818_ImuA->       424877133    <-DinB<-SRAP*<-?||ImuA->ImuB+ImuB-C->                                      Rleg5DRAFT_1560                 225   bacteria>proteobacteria>alphaproteobacteria    Rhizobium leguminosarum bv. viciae WSM1455                                   hypothetical protein Rleg5DRAFT_1560 [Rhizobium leguminosarum bv. viciae WSM1455].                                             424877130_?->424877131_?-><-424877132_DinB<-424877133_SRAP*<-424877134_?||424877135_ImuA->424877136_ImuB+ImuB-C->       408787408    <-ImuA||?->?->SRAP*->                                                      C241_15208                      258   bacteria>proteobacteria>alphaproteobacteria    Rhizobium lupini HPC(L)                                                      hypothetical protein C241_15208 [Rhizobium lupini HPC(L)].                                                                     <-408787405_ImuA||408787406_?->408787407_?->408787408_SRAP*->408787409_?->408787410_?->408787411_?->       409438641    <-ATP-dependent-ligase<-SRAP*||SNase-><-?||ImuA->                          -                               257   bacteria>proteobacteria>alphaproteobacteria    Rhizobium mesoamericanum STM3625                                             conserved hypothetical protein [Rhizobium mesoamericanum STM3625].                                                             409438638_?->409438639_?-><-409438640_ATP-dependent-ligase<-409438641_SRAP*||409438642_SNase-><-409438643_?||409438644_ImuA->       402487091    <-ImuA||?->DinB->SRAP*->                                                   RCCGE510_05277                  196   bacteria>proteobacteria>alphaproteobacteria    Rhizobium sp. CCGE 510                                                       hypothetical protein RCCGE510_05277 [Rhizobium sp. CCGE 510].                                                                  <-402487088_ImuA||402487089_?->402487090_DinB->402487091_SRAP*-><-402487092_?||402487093_?-><-402487094_?       420239996    Family_Y_DNApol->DnaE2->?->SRAP->SRAP*->                                   PMI07_02028                     214   bacteria>proteobacteria>alphaproteobacteria    Rhizobium sp. CF080                                                          hypothetical protein PMI07_02028 [Rhizobium sp. CF080].                                                                        420239992_Family_Y_DNApol->420239993_DnaE2->420239994_?->420239995_SRAP->420239996_SRAP*-><-420239997_?<-420239998_?<-420239999_?       418936246    <-ImuA||UvrD->SRAP*->DinB->                                                PDO_1142                        225   bacteria>proteobacteria>alphaproteobacteria    Rhizobium sp. PDO1-076                                                       protein of unknown function DUF159 [Rhizobium sp. PDO1-076].                                                                   <-418936244_ImuA||418936245_UvrD->418936246_SRAP*->418936247_DinB-><-418936248_?<-418936249_?       378827937    <-ImuB+ImuB-C<-ImuA||DinB-><-SRAP||SRAP*->                                 -                               196   bacteria>proteobacteria>alphaproteobacteria    Sinorhizobium fredii HH103                                                   hypothetical protein SFHH103_03352 [Sinorhizobium fredii HH103].                                                               <-378827933_ImuB+ImuB-C<-378827934_ImuA||378827935_DinB-><-378827936_SRAP||378827937_SRAP*->378827938_?-><-378827939_?<-378827940_?       470184462    <-SRAP||SRAP*->?->ImuA->ImuB+ImuB-C->                                      SM2011_a0883                    165   bacteria>proteobacteria>alphaproteobacteria    Sinorhizobium meliloti 2011                                                  hypothetical protein SM2011_a0883 [Sinorhizobium meliloti 2011].                                                               <-470184459_?<-470184460_?<-470184461_SRAP||470184462_SRAP*->470184463_?->470184464_ImuA->470184465_ImuB+ImuB-C->       334318886    <-SRAP*||ImuA->ImuB+ImuB-C->DnaE2->                                        Sinme_5784                      196   bacteria>proteobacteria>alphaproteobacteria    Sinorhizobium meliloti AK83                                                  hypothetical protein Sinme_5784 [Sinorhizobium meliloti AK83].                                                                 <-334318883_?||334318884_?->334318885_?-><-334318886_SRAP*||334318887_ImuA->334318888_ImuB+ImuB-C->334318889_DnaE2->       418404268    <-ImuA<-?||?->SRAP*->SRAP->                                                SM0020_29185                    196   bacteria>proteobacteria>alphaproteobacteria    Sinorhizobium meliloti CCNWSX0020                                            hypothetical protein SM0020_29185 [Sinorhizobium meliloti CCNWSX0020].                                                         <-418404265_ImuA<-418404266_?||418404267_?->418404268_SRAP*->418404269_SRAP->       384540866    <-ImuB+ImuB-C<-ImuA||ImuB+ImuB-C-><-SRAP||SRAP*->                          SM11_pC1067                     196   bacteria>proteobacteria>alphaproteobacteria    Sinorhizobium meliloti SM11                                                  hypothetical protein SM11_pC1067 [Sinorhizobium meliloti SM11].                                                                <-384540862_ImuB+ImuB-C<-384540863_ImuA||384540864_ImuB+ImuB-C-><-384540865_SRAP||384540866_SRAP*->384540867_?->384540868_?->384540869_?->       190410006    <-DnaE2<-?||?-><-SRAP*<-ATP-dependent-ligase||ImuA->ImuB+ImuB-C->          pSmeSM11b_p031                  257   bacteria>proteobacteria>alphaproteobacteria    Sinorhizobium meliloti SM11                                                  hypothetical protein [Sinorhizobium meliloti SM11].                                                                            <-190410003_DnaE2<-190410004_?||190410005_?-><-190410006_SRAP*<-190410007_ATP-dependent-ligase||190410008_ImuA->190410009_ImuB+ImuB-C->       294010400    ImuA->ImuB+ImuB-C->DnaE2-><-SRAP*||SRAP->                                  SJA_C1-04140                    196   bacteria>proteobacteria>alphaproteobacteria    Sphingobium japonicum UT26S                                                  hypothetical protein SJA_C1-04140 [Sphingobium japonicum UT26S].                                                               294010397_ImuA->294010398_ImuB+ImuB-C->294010399_DnaE2-><-294010400_SRAP*||294010401_SRAP-><-294010402_?||294010403_?->294010404_?->       398386558    <-DnaE2<-ImuB+ImuB-C<-ImuA||SRAP->SRAP*->                                  PMI04_04341                     201   bacteria>proteobacteria>alphaproteobacteria    Sphingobium sp. AP49                                                         hypothetical protein PMI04_04341 [Sphingobium sp. AP49].                                                                       <-398386554_DnaE2<-398386555_ImuB+ImuB-C<-398386556_ImuA||398386557_SRAP->398386558_SRAP*-><-398386559_?||398386560_?->398386561_?->       347529036    <-SRAP||SRAP*->ImuA->ImuB+ImuB-C->DnaE2->                                  -                               215   bacteria>proteobacteria>alphaproteobacteria    Sphingobium sp. SYK-6                                                        hypothetical protein SLG_26510 [Sphingobium sp. SYK-6].                                                                        <-347529033_?||347529034_?-><-347529035_SRAP||347529036_SRAP*->347529037_ImuA->347529038_ImuB+ImuB-C->347529039_DnaE2->       427409847    <-DnaE2<-ImuB+ImuB-C<-ImuA||SRAP->SRAP*->                                  -                               201   bacteria>proteobacteria>alphaproteobacteria    Sphingobium yanoikuyae ATCC 51230                                            hypothetical protein HMPREF9718_02523 [Sphingobium yanoikuyae ATCC 51230].                                                     <-427409843_DnaE2<-427409844_ImuB+ImuB-C<-427409845_ImuA||427409846_SRAP->427409847_SRAP*-><-427409848_?||427409849_?->427409850_?->       427412044    SRAP->SRAP*-><-DnaE2                                                       -                               214   bacteria>proteobacteria>alphaproteobacteria    Sphingobium yanoikuyae ATCC 51230                                            hypothetical protein HMPREF9718_04720 [Sphingobium yanoikuyae ATCC 51230].                                                     427412040_?->427412041_?->427412042_?->427412043_SRAP->427412044_SRAP*-><-427412045_DnaE2||427412046_?-><-427412047_?       381203304    <-SRAP*<-?<-SRAP||ImuA->ImuB+ImuB-C->DnaE2->                               SyanX_010100022438              167   bacteria>proteobacteria>alphaproteobacteria    Sphingobium yanoikuyae XLDN2-5                                               hypothetical protein SyanX_22438 [Sphingobium yanoikuyae XLDN2-5].                                                             381203301_?-><-381203302_?<-381203303_?<-381203304_SRAP*<-381203305_?<-381203306_SRAP||381203307_ImuA->381203308_ImuB+ImuB-C->381203309_DnaE2->       470215756    SRAP*->?-><-?||ImuA->                                                      -                               221   bacteria>proteobacteria>alphaproteobacteria    Sphingomonas sp. MM-1                                                        hypothetical protein G432_19020 [Sphingomonas sp. MM-1].                                                                       <-470215753_?<-470215754_?<-470215755_?||470215756_SRAP*->470215757_?-><-470215758_?||470215759_ImuA->       470214900    <-SRAP||SRAP*-><-DnaE2<-ImuB+ImuB-C<-ImuA                                  -                               206   bacteria>proteobacteria>alphaproteobacteria    Sphingomonas sp. MM-1                                                        hypothetical protein G432_14745 [Sphingomonas sp. MM-1].                                                                       470214896_?-><-470214897_?<-470214898_?<-470214899_SRAP||470214900_SRAP*-><-470214901_DnaE2<-470214902_ImuB+ImuB-C<-470214903_ImuA       395491574    <-DnaE2<-ImuB+ImuB-C<-ImuA||SRAP->?->SRAP*->                               SPAM26_010100007075             217   bacteria>proteobacteria>alphaproteobacteria    Sphingomonas sp. PAMC 26617                                                  hypothetical protein SPAM26_07075 [Sphingomonas sp. PAMC 26617].                                                               <-395491569_DnaE2<-395491570_ImuB+ImuB-C<-395491571_ImuA||395491572_SRAP->395491573_?->395491574_SRAP*-><-395491575_?||395491576_?->395491577_?->       154244761    <-DnaE2<-ImuB+ImuB-C<-ImuA||SRAP*->                                        -                               229   bacteria>proteobacteria>alphaproteobacteria    Xanthobacter autotrophicus Py2                                               hypothetical protein Xaut_0811 [Xanthobacter autotrophicus Py2].                                                               <-154244758_DnaE2<-154244759_ImuB+ImuB-C<-154244760_ImuA||154244761_SRAP*->154244762_?->154244763_?->154244764_?->       452753327    <-SRAP*<-?<-?||ImuA->                                                      C725_2846                       189   bacteria>proteobacteria>alphaproteobacteria    alpha proteobacterium JLT2015                                                hypothetical protein C725_2846 [alpha proteobacterium JLT2015].                                                                <-452753324_?<-452753325_?||452753326_?-><-452753327_SRAP*<-452753328_?<-452753329_?||452753330_ImuA->       153010467    <-SRAP*||ImuA->ImuB+ImuB-C->DnaE2->                                        Oant_3144                       256   bacteria>proteobacteria>alphaproteobacteria    Ochrobactrum anthropi ATCC 49188                                             hypothetical protein Oant_3144 [Ochrobactrum anthropi ATCC 49188].                                                             153010464_?->153010465_?-><-153010466_?<-153010467_SRAP*||153010468_ImuA->153010469_ImuB+ImuB-C->153010470_DnaE2->       384529378    ImuB-C->?->SRAP*->                                                         -                               224   bacteria>proteobacteria>alphaproteobacteria    Sinorhizobium meliloti BL225C                                                hypothetical protein [Sinorhizobium meliloti BL225C].                                                                          384529375_?->384529376_ImuB-C->384529377_?->384529378_SRAP*-><-384529379_?||384529380_?-><-384529381_?       405379046    <-ImuB-C<-SRAP*                                                            PMI11_02928                     152   bacteria>proteobacteria>alphaproteobacteria    Rhizobium sp. CF142                                                          hypothetical protein PMI11_02928, partial [Rhizobium sp. CF142].                                                               <-405379043_?<-405379044_?<-405379045_ImuB-C<-405379046_SRAP*       218680946    <-ImuB-C<-SRAP*                                                            RetlC8_010100019703             158   bacteria>proteobacteria>alphaproteobacteria    Rhizobium etli CIAT 894                                                      hypothetical protein RetlC8_19703, partial [Rhizobium etli CIAT 894].                                                          <-218680943_?<-218680944_?<-218680945_ImuB-C<-218680946_SRAP*       424895979    <-ImuB+ImuB-C||?->?->SRAP*->                                               Rleg4DRAFT_1865                 256   bacteria>proteobacteria>alphaproteobacteria    Rhizobium leguminosarum bv. trifolii WSM2297                                 hypothetical protein Rleg4DRAFT_1865 [Rhizobium leguminosarum bv. trifolii WSM2297].                                           <-424895976_ImuB+ImuB-C||424895977_?->424895978_?->424895979_SRAP*->424895980_?-><-424895981_?<-424895982_?       440227249    SRAP*->ImuB-C->                                                            -                               242   bacteria>proteobacteria>alphaproteobacteria    Rhizobium tropici CIAT 899                                                   hypothetical protein RTCIAT899_CH12065 [Rhizobium tropici CIAT 899].                                                           <-440227246_?<-440227247_?||440227248_?->440227249_SRAP*->440227250_ImuB-C->440227251_?->440227252_?->       76808876     <-DnaE2||SRAP*-><-?<-?<-Resolvase                                          -                               64    bacteria>proteobacteria>betaproteobacteria     Burkholderia pseudomallei 1710b                                              gp33 [Burkholderia pseudomallei 1710b].                                                                                        76808808_?->76810264_?-><-76809389_DnaE2||76808876_SRAP*-><-76810333_?<-76811478_?<-76808986_Resolvase       323530204    <-SRAP*||ImuA->                                                            -                               320   bacteria>proteobacteria>betaproteobacteria     Burkholderia sp. CCGE1001                                                    hypothetical protein BC1001_5932 [Burkholderia sp. CCGE1001].                                                                  323530201_?-><-323530202_?||323530203_?-><-323530204_SRAP*||323530205_ImuA->323530206_?-><-323530207_?       295676322    <-SRAP*<-DnaE2<-ImuB+ImuB-C                                                BC1002_1251                     319   bacteria>proteobacteria>betaproteobacteria     Burkholderia sp. CCGE1002                                                    hypothetical protein BC1002_1251 [Burkholderia sp. CCGE1002].                                                                  <-295676319_?<-295676320_?<-295676321_?<-295676322_SRAP*<-295676323_DnaE2<-295676324_ImuB+ImuB-C||295676325_?->       307730065    <-SRAP*<-DnaE2<-ImuB+ImuB-C<-ImuA                                          -                               319   bacteria>proteobacteria>betaproteobacteria     Burkholderia sp. CCGE1003                                                    hypothetical protein [Burkholderia sp. CCGE1003].                                                                              307730062_?-><-307730063_?||307730064_?-><-307730065_SRAP*<-307730066_DnaE2<-307730067_ImuB+ImuB-C<-307730068_ImuA       385210529    ImuA->ImuB+ImuB-C->DnaE2->SRAP*->                                          BCh11DRAFT_07724                319   bacteria>proteobacteria>betaproteobacteria     Burkholderia sp. Ch1-1                                                       hypothetical protein BCh11DRAFT_07724 [Burkholderia sp. Ch1-1].                                                                385210526_ImuA->385210527_ImuB+ImuB-C->385210528_DnaE2->385210529_SRAP*->385210530_?->385210531_?-><-385210532_?       209515640    <-SRAP*<-DnaE2<-ImuB+ImuB-C<-ImuA                                          BH160DRAFT_0780                 319   bacteria>proteobacteria>betaproteobacteria     Burkholderia sp. H160                                                        putative uncharacterized conserved protein [Burkholderia sp. H160].                                                            <-209515637_?<-209515638_?<-209515639_?<-209515640_SRAP*<-209515641_DnaE2<-209515642_ImuB+ImuB-C<-209515643_ImuA       413960088    <-SRAP*<-?<-?||ImuA->                                                      BURK_009246                     319   bacteria>proteobacteria>betaproteobacteria     Burkholderia sp. SJ98                                                        hypothetical protein BURK_009246 [Burkholderia sp. SJ98].                                                                      <-413960085_?<-413960086_?||413960087_?-><-413960088_SRAP*<-413960089_?<-413960090_?||413960091_ImuA->       390566757    <-ImuA||SRAP*->                                                            WQE_00805                       320   bacteria>proteobacteria>betaproteobacteria     Burkholderia terrae BS001                                                    hypothetical protein WQE_00805 [Burkholderia terrae BS001].                                                                    <-390566754_?<-390566755_?<-390566756_ImuA||390566757_SRAP*->390566758_?->390566759_?->390566760_?->       390569715    ImuA->?-><-SRAP*                                                           WQE_15351                       228   bacteria>proteobacteria>betaproteobacteria     Burkholderia terrae BS001                                                    hypothetical protein WQE_15351 [Burkholderia terrae BS001].                                                                    <-390569712_?||390569713_ImuA->390569714_?-><-390569715_SRAP*<-390569716_?<-390569717_?||390569718_?->       91785552     <-ImuA||SRAP*->                                                            -                               227   bacteria>proteobacteria>betaproteobacteria     Burkholderia xenovorans LB400                                                hypothetical protein Bxe_A0226 [Burkholderia xenovorans LB400].                                                                <-91785549_?<-91785550_?<-91785551_ImuA||91785552_SRAP*->91785553_?->91785554_?->91785555_?->       188591400    <-ATP-dependent-ligase||SRAP*->?-><-?<-DnaE2                               pRALTA_0096                     321   bacteria>proteobacteria>betaproteobacteria     Cupriavidus taiwanensis LMG 19424                                            hypothetical protein pRALTA_0096 [Cupriavidus taiwanensis LMG 19424].                                                          188591397_?-><-188591398_?<-188591399_ATP-dependent-ligase||188591400_SRAP*->188591401_?-><-188591402_?<-188591403_DnaE2       445498653    ImuB+ImuB-C->DnaE2->?->SRAP*->                                             -                               267   bacteria>proteobacteria>betaproteobacteria     Janthinobacterium sp. HH01                                                   hypothetical protein Jab_2c22830 [Janthinobacterium sp. HH01].                                                                 445498650_ImuB+ImuB-C->445498651_DnaE2->445498652_?->445498653_SRAP*-><-445498654_?<-445498655_?<-445498656_?       383757666    <-DnaE2<-ImuB+ImuB-C<-SulA||SRAP*->                                        -                               319   bacteria>proteobacteria>betaproteobacteria     Rubrivivax gelatinosus IL144                                                 hypothetical protein RGE_18110 [Rubrivivax gelatinosus IL144].                                                                 <-383757663_DnaE2<-383757664_ImuB+ImuB-C<-383757665_SulA||383757666_SRAP*->383757667_?->383757668_?-><-383757669_?       217968738    SRAP*->?-><-DnaE2                                                          -                               243   bacteria>proteobacteria>betaproteobacteria     Thauera sp. MZ1T                                                             hypothetical protein Tmz1t_0284 [Thauera sp. MZ1T].                                                                            <-217968735_?<-217968736_?<-217968737_?||217968738_SRAP*->217968739_?-><-217968740_DnaE2<-217968741_?       296136340    <-SRAP*||ImuA->ImuB+ImuB-C->DnaE2->                                        Tint_1887                       224   bacteria>proteobacteria>betaproteobacteria     Thiomonas intermedia K12                                                     hypothetical protein Tint_1887 [Thiomonas intermedia K12].                                                                     <-296136337_?<-296136338_?||296136339_?-><-296136340_SRAP*||296136341_ImuA->296136342_ImuB+ImuB-C->296136343_DnaE2->       410692869    <-DnaE2<-ImuB+ImuB-C<-SulA||SRAP*->                                        THI_0947                        229   bacteria>proteobacteria>betaproteobacteria     Thiomonas sp. 3As                                                            Conserved hypothetical protein [Thiomonas sp. 3As].                                                                            <-410692866_DnaE2<-410692867_ImuB+ImuB-C<-410692868_SulA||410692869_SRAP*-><-410692870_?<-410692871_?<-410692872_?       239817502    <-DnaE2<-ImuB+ImuB-C<-SulA||SRAP*->                                        -                               310   bacteria>proteobacteria>betaproteobacteria     Variovorax paradoxus S110                                                    hypothetical protein Vapar_4539 [Variovorax paradoxus S110].                                                                   <-239817499_DnaE2<-239817500_ImuB+ImuB-C<-239817501_SulA||239817502_SRAP*->239817503_?->239817504_?->239817505_?->       170697438    <-DnaE2<-ImuB+ImuB-C<-ImuA<-SRAP*                                          BamIOP4010DRAFT_0591            305   bacteria>proteobacteria>betaproteobacteria     Burkholderia ambifaria IOP40-10                                              putative uncharacterized conserved protein [Burkholderia ambifaria IOP40-10].                                                  <-170697435_DnaE2<-170697436_ImuB+ImuB-C<-170697437_ImuA<-170697438_SRAP*||170697439_?->170697440_?->170697441_?->       116687204    SRAP*->ImuA->ImuB+ImuB-C->DnaE2->                                          Bcen2424_6828                   303   bacteria>proteobacteria>betaproteobacteria     Burkholderia cenocepacia HI2424                                              hypothetical protein Bcen2424_6828 [Burkholderia cenocepacia HI2424].                                                          116687201_?->116687202_?->116687203_?->116687204_SRAP*->116687205_ImuA->116687206_ImuB+ImuB-C->116687207_DnaE2->       116687244    SRAP*->ImuA->ImuB+ImuB-C->DnaE2->                                          Bcen2424_6868                   303   bacteria>proteobacteria>betaproteobacteria     Burkholderia cenocepacia HI2424                                              hypothetical protein Bcen2424_6868 [Burkholderia cenocepacia HI2424].                                                          116687241_?->116687242_?->116687243_?->116687244_SRAP*->116687245_ImuA->116687246_ImuB+ImuB-C->116687247_DnaE2->       221215345    SRAP*->ImuA->ImuB+ImuB-C->DnaE2->                                          BURMUCGD1_6587                  304   bacteria>proteobacteria>betaproteobacteria     Burkholderia multivorans CGD1                                                conserved hypothetical protein [Burkholderia multivorans CGD1].                                                                <-221215342_?<-221215343_?||221215344_?->221215345_SRAP*->221215346_ImuA->221215347_ImuB+ImuB-C->221215348_DnaE2->       170690325    SulA->ImuB+ImuB-C->DnaE2->SRAP*->                                          BgramDRAFT_0301                 320   bacteria>proteobacteria>betaproteobacteria     Burkholderia graminis C4D1M                                                  putative uncharacterized conserved protein [Burkholderia graminis C4D1M].                                                      170690322_SulA->170690323_ImuB+ImuB-C->170690324_DnaE2->170690325_SRAP*->170690326_?-><-170690327_?<-170690328_?       186474588    ImuA->SRAP*->                                                              Bphy_7577                       320   bacteria>proteobacteria>betaproteobacteria     Burkholderia phymatum STM815                                                 hypothetical protein Bphy_7577 [Burkholderia phymatum STM815].                                                                 <-186474585_?<-186474586_?||186474587_ImuA->186474588_SRAP*-><-186474589_?<-186474590_?||186474591_?->       392547904    <-DnaE2<-ImuB+ImuB-C<-ImuA||SRAP*->                                        PrubA2_010100016086             237   bacteria>proteobacteria>gammaproteobacteria    Pseudoalteromonas rubra ATCC 29570                                           hypothetical protein PrubA2_16086 [Pseudoalteromonas rubra ATCC 29570].                                                        <-392547901_DnaE2<-392547902_ImuB+ImuB-C<-392547903_ImuA||392547904_SRAP*-><-392547905_?<-392547906_?||392547907_?->       442611719    <-DnaE2<-ImuB+ImuB-C<-ImuA||SRAP*->                                        PALB_34230                      230   bacteria>proteobacteria>gammaproteobacteria    Pseudoalteromonas luteoviolacea B = ATCC 29581                               conserved hypothetical protein [Pseudoalteromonas luteoviolacea B = ATCC 29581].                                               <-442611716_DnaE2<-442611717_ImuB+ImuB-C<-442611718_ImuA||442611719_SRAP*-><-442611720_?<-442611721_?||442611722_?->       443471394    <-SRAP*||?->SulA->ImuB+ImuB-C->                                            ppKF707_4742                    231   bacteria>proteobacteria>gammaproteobacteria    Pseudomonas pseudoalcaligenes KF707                                          protein of unknown function DUF159 [Pseudomonas pseudoalcaligenes KF707].                                                      <-443471391_?<-443471392_?<-443471393_?<-443471394_SRAP*||443471395_?->443471396_SulA->443471397_ImuB+ImuB-C->       469818142    <-SRAP*||?-><-DnaE2                                                        -                               322   bacteria>proteobacteria>gammaproteobacteria    Rhodanobacter sp. 2APBS1                                                     hypothetical protein R2APBS1_1867 [Rhodanobacter sp. 2APBS1].                                                                  <-469818139_?<-469818140_?||469818141_?-><-469818142_SRAP*||469818143_?-><-469818144_DnaE2||469818145_?->       330822176    <-SRAP*<-?||SulA->ImuB+ImuB-C->                                            bgla_2p0880                     252   bacteria>proteobacteria>betaproteobacteria     Burkholderia gladioli BSR3                                                   ATP synthase protein I [Burkholderia gladioli BSR3].                                                                           330822173_?-><-330822174_?<-330822175_?<-330822176_SRAP*<-330822177_?||330822178_SulA->330822179_ImuB+ImuB-C->       206479934    <-ImuB+ImuB-C<-SulA||?-><-SRAP*                                            pBCA008                         320   bacteria>proteobacteria>betaproteobacteria     Burkholderia cenocepacia J2315                                               hypothetical protein pBCA008 [Burkholderia cenocepacia J2315].                                                                 <-206479931_ImuB+ImuB-C<-206479932_SulA||206479933_?-><-206479934_SRAP*<-206479935_?<-206479936_?<-206479937_?       420239996    Family_Y_DNApol->DnaE2->?->SRAP->SRAP*->                                   PMI07_02028                     214   bacteria>proteobacteria>alphaproteobacteria    Rhizobium sp. CF080                                                          hypothetical protein PMI07_02028 [Rhizobium sp. CF080].                                                                        420239992_Family_Y_DNApol->420239993_DnaE2->420239994_?->420239995_SRAP->420239996_SRAP*-><-420239997_?<-420239998_?<-420239999_?        # Ku-ATP-dependent DNA ligase and ZnR associated       409124139    <-SSB<-SRAP*||Ku->ATP-dependent-ligase->                                   GCBA3_010100012386              254   bacteria>bacteroidetes                         Gillisia sp. CBA3202                                                         hypothetical protein GCBA3_12386 [Gillisia sp. CBA3202].                                                                       <-409124137_?<-409124138_SSB<-409124139_SRAP*||409124140_Ku->409124141_ATP-dependent-ligase->       403385211    <-SRAP<-?||SRAP*->                                                         KJC30_010100010943              104   bacteria>firmicutes                            Kurthia sp. JC30                                                             hypothetical protein KJC30_10943 [Kurthia sp. JC30].                                                                           403385208_?-><-403385209_SRAP<-403385210_?||403385211_SRAP*-><-403385212_?<-403385213_?||403385214_?->       160934665    <-Ku||DinB->ImuBC->SRAP*->                                                 CLOLEP_03538                    187   bacteria>firmicutes                            Clostridium leptum DSM 753                                                   hypothetical protein CLOLEP_03538 [Clostridium leptum DSM 753].                                                                <-160934662_Ku||160934663_DinB->160934664_ImuBC->160934665_SRAP*-><-160934666_?||160934667_?->160934668_?->       414176364    <-ZnR2||SRAP*-><-?||?->Ku->                                                -                               261   bacteria>proteobacteria>alphaproteobacteria    Afipia broomeae ATCC 49717                                                   hypothetical protein HMPREF9695_04239 [Afipia broomeae ATCC 49717].                                                            414176361_?->414176362_?-><-414176363_ZnR2||414176364_SRAP*-><-414176365_?||414176366_?->414176367_Ku->       414176059    <-Ku||ZnR1->ZnR2->SRAP*->DinB->                                            -                               241   bacteria>proteobacteria>alphaproteobacteria    Afipia broomeae ATCC 49717                                                   hypothetical protein HMPREF9695_03934 [Afipia broomeae ATCC 49717].                                                            <-414176056_Ku||414176057_ZnR1->414176058_ZnR2->414176059_SRAP*->414176060_DinB->414176061_?->414176062_?->       414172033    <-SRAP*<-ZnR2<-?||Ku->                                                     -                               231   bacteria>proteobacteria>alphaproteobacteria    Afipia broomeae ATCC 49717                                                   hypothetical protein HMPREF9695_00590 [Afipia broomeae ATCC 49717].                                                            <-414172030_?<-414172031_?||414172032_?-><-414172033_SRAP*<-414172034_ZnR2<-414172035_?||414172036_Ku->       338971676    <-Ku||ZnR1->ZnR2->SRAP*->                                                  -                               241   bacteria>proteobacteria>alphaproteobacteria    Bradyrhizobiaceae bacterium SG-6C                                            protein of unknown function DUF159 [Bradyrhizobiaceae bacterium SG-6C].                                                        <-338971673_Ku||338971674_ZnR1->338971675_ZnR2->338971676_SRAP*-><-338971677_?<-338971678_?<-338971679_?       27381218     ZnR2->SRAP*->                                                              -                               243   bacteria>proteobacteria>alphaproteobacteria    Bradyrhizobium diazoefficiens USDA 110                                       hypothetical protein blr6107 [Bradyrhizobium diazoefficiens USDA 110].                                                         <-27381215_?<-27381216_?||27381217_ZnR2->27381218_SRAP*-><-27381219_?||27381220_?-><-27381221_?       384215690    <-DinB<-SRAP*<-ZnR2<-ZnR1||Ku->                                            -                               241   bacteria>proteobacteria>alphaproteobacteria    Bradyrhizobium japonicum USDA 6                                              hypothetical protein BJ6T_19890 [Bradyrhizobium japonicum USDA 6].                                                             384215687_?->384215688_?-><-384215689_DinB<-384215690_SRAP*<-384215691_ZnR2<-384215692_ZnR1||384215693_Ku->       148254742    ZnR1->ZnR2->SRAP*->                                                        -                               244   bacteria>proteobacteria>alphaproteobacteria    Bradyrhizobium sp. BTAi1                                                     hypothetical protein BBta_3320 [Bradyrhizobium sp. BTAi1].                                                                     148254739_?->148254740_ZnR1->148254741_ZnR2->148254742_SRAP*->148254743_?-><-148254744_?<-148254745_?       148254847    ZnR1->ZnR2->SRAP*->?->UspA->UspA->                                         -                               244   bacteria>proteobacteria>alphaproteobacteria    Bradyrhizobium sp. BTAi1                                                     hypothetical protein BBta_3432 [Bradyrhizobium sp. BTAi1].                                                                     <-148254844_?||148254845_ZnR1->148254846_ZnR2->148254847_SRAP*->148254848_?->148254849_UspA->148254850_UspA->       421603408    <-Ku||SRAP*->                                                              BCCGELA001_33513                122   bacteria>proteobacteria>alphaproteobacteria    Bradyrhizobium sp. CCGE-LA001                                                hypothetical protein BCCGELA001_33513 [Bradyrhizobium sp. CCGE-LA001].                                                         <-421603405_?<-421603406_?<-421603407_Ku||421603408_SRAP*-><-421603409_?<-421603410_?<-421603411_?       367478223    ZnR1->ZnR2->SRAP*->                                                        BRAO285_880026                  244   bacteria>proteobacteria>alphaproteobacteria    Bradyrhizobium sp. ORS 285                                                   conserved hypothetical protein [Bradyrhizobium sp. ORS 285].                                                                   <-367478220_?||367478221_ZnR1->367478222_ZnR2->367478223_SRAP*->367478224_?->367478225_?-><-367478226_?       383772056    ZnR2->SRAP*->                                                              -                               240   bacteria>proteobacteria>alphaproteobacteria    Bradyrhizobium sp. S23321                                                    hypothetical protein S23_38150 [Bradyrhizobium sp. S23321].                                                                    <-383772053_?<-383772054_?||383772055_ZnR2->383772056_SRAP*->383772057_?->383772058_?->383772059_?->       386400441    <-SRAP*<-?<-DnaE2<-ZnR2                                                    Bra1253DRAFT_06090              254   bacteria>proteobacteria>alphaproteobacteria    Bradyrhizobium sp. WSM1253                                                   hypothetical protein Bra1253DRAFT_06090 [Bradyrhizobium sp. WSM1253].                                                          386400438_?->386400439_?-><-386400440_?<-386400441_SRAP*<-386400442_?<-386400443_DnaE2<-386400444_ZnR2       374573392    <-SRAP*<-ZnR2<-ZnR1||Ku->                                                  Bra471DRAFT_01948               240   bacteria>proteobacteria>alphaproteobacteria    Bradyrhizobium sp. WSM471                                                    hypothetical protein Bra471DRAFT_01948 [Bradyrhizobium sp. WSM471].                                                            <-374573389_?||374573390_?-><-374573391_?<-374573392_SRAP*<-374573393_ZnR2<-374573394_ZnR1||374573395_Ku->       374577755    ZnR2->SRAP*->                                                              Bra471DRAFT_06433               241   bacteria>proteobacteria>alphaproteobacteria    Bradyrhizobium sp. WSM471                                                    hypothetical protein Bra471DRAFT_06433 [Bradyrhizobium sp. WSM471].                                                            <-374577752_?<-374577753_?||374577754_ZnR2->374577755_SRAP*-><-374577756_?||374577757_?-><-374577758_?       386397205    <-Ku||ZnR1->ZnR2->SRAP*->                                                  Bra1253DRAFT_02716              221   bacteria>proteobacteria>alphaproteobacteria    Bradyrhizobium sp. WSM1253                                                   hypothetical protein Bra1253DRAFT_02716 [Bradyrhizobium sp. WSM1253].                                                          <-386397202_Ku||386397203_ZnR1->386397204_ZnR2->386397205_SRAP*-><-386397206_?<-386397207_?||386397208_?->       429767754    <-Ku<-?<-?<-SRAP*                                                          HMPREF0185_00204                243   bacteria>proteobacteria>alphaproteobacteria    Brevundimonas diminuta 470-4                                                 hypothetical protein HMPREF0185_00204 [Brevundimonas diminuta 470-4].                                                          <-429767751_Ku<-429767752_?<-429767753_?<-429767754_SRAP*<-429767755_?<-429767756_?<-429767757_?       85716261     ZnR2->SRAP*->?-><-?||ATP-dependent-ligase->                                NB311A_19642                    241   bacteria>proteobacteria>alphaproteobacteria    Nitrobacter sp. Nb-311A                                                      hypothetical protein NB311A_19642 [Nitrobacter sp. Nb-311A].                                                                   <-85716258_?<-85716259_?||85716260_ZnR2->85716261_SRAP*->85716262_?-><-85716263_?||85716264_ATP-dependent-ligase->       241666873    Ku->Ku->SRAP*->                                                            Rleg_6967                       198   bacteria>proteobacteria>alphaproteobacteria    Rhizobium leguminosarum bv. trifolii WSM1325                                 hypothetical protein Rleg_6967 [Rhizobium leguminosarum bv. trifolii WSM1325].                                                 <-241666870_?||241666871_Ku->241666872_Ku->241666873_SRAP*-><-241666874_?||241666875_?->241666876_?->       192292177    ZnR1->ZnR2->SRAP*->DinB->                                                  -                               246   bacteria>proteobacteria>alphaproteobacteria    Rhodopseudomonas palustris TIE-1                                             hypothetical protein Rpal_3808 [Rhodopseudomonas palustris TIE-1].                                                             <-192292174_?||192292175_ZnR1->192292176_ZnR2->192292177_SRAP*->192292178_DinB-><-192292179_?<-192292180_?       91974492     ZnR2->SRAP*->                                                              -                               230   bacteria>proteobacteria>alphaproteobacteria    Rhodopseudomonas palustris BisB5                                             hypothetical protein RPD_0010 [Rhodopseudomonas palustris BisB5].                                                              <-91974489_?<-91974490_?||91974491_ZnR2->91974492_SRAP*-><-91974493_?<-91974494_?<-91974495_?       192289546    ZnR2->SRAP*->                                                              -                               241   bacteria>proteobacteria>alphaproteobacteria    Rhodopseudomonas palustris TIE-1                                             hypothetical protein Rpal_1134 [Rhodopseudomonas palustris TIE-1].                                                             <-192289543_?<-192289544_?||192289545_ZnR2->192289546_SRAP*->192289547_?->192289548_?->192289549_?->       91978100     ZnR1->ZnR2->SRAP*->DinB->                                                  -                               241   bacteria>proteobacteria>alphaproteobacteria    Rhodopseudomonas palustris BisB5                                             hypothetical protein RPD_3635 [Rhodopseudomonas palustris BisB5].                                                              <-91978097_?||91978098_ZnR1->91978099_ZnR2->91978100_SRAP*->91978101_DinB-><-91978102_?<-91978103_?       339324038    <-Ku<-ATP-dependent-ligase||SRAP*->?-><-DnaE2<-ImuB+ImuB-C                 CNE_BB2p02860                   321   bacteria>proteobacteria>betaproteobacteria     Cupriavidus necator N-1                                                      hypothetical protein CNE_BB2p02860 [Cupriavidus necator N-1].                                                                  <-339324035_?<-339324036_Ku<-339324037_ATP-dependent-ligase||339324038_SRAP*->339324039_?-><-339324040_DnaE2<-339324041_ImuB+ImuB-C       413959086    <-Ku||SRAP*->                                                              BURK_004172                     236   bacteria>proteobacteria>betaproteobacteria     Burkholderia sp. SJ98                                                        hypothetical protein BURK_004172 [Burkholderia sp. SJ98].                                                                      <-413959083_?<-413959084_?<-413959085_Ku||413959086_SRAP*->413959087_?-><-413959088_?<-413959089_?       399001936    <-SRAP*||?-><-Ku                                                           PMI21_03225                     225   bacteria>proteobacteria>gammaproteobacteria    Pseudomonas sp. GM18                                                         hypothetical protein PMI21_03225 [Pseudomonas sp. GM18].                                                                       <-399001933_?<-399001934_?<-399001935_?<-399001936_SRAP*||399001937_?-><-399001938_Ku||399001939_?->        # AB-hydrolase associated       383782474    <-Sigma<-abhydrolase<-?<-SRAP*                                             -                               230   bacteria>actinobacteria                        Actinoplanes missouriensis 431                                               hypothetical protein AMIS_73050 [Actinoplanes missouriensis 431].                                                              <-383782471_Sigma<-383782472_abhydrolase<-383782473_?<-383782474_SRAP*||383782475_?->383782476_?->383782477_?->       256380285    <-Sigma||aminoacyl-tRNA_deacylase-><-abhydrolase<-SRAP*                    -                               253   bacteria>actinobacteria                        Actinosynnema mirum DSM 43827                                                hypothetical protein Amir_6295 [Actinosynnema mirum DSM 43827].                                                                <-256380282_Sigma||256380283_aminoacyl-tRNA_deacylase-><-256380284_abhydrolase<-256380285_SRAP*||256380286_?->256380287_?->256380288_?->       309812141    <-DoxX||?->SRAP*->abhydrolase->Sigma->                                     HMPREF0321_2716                 281   bacteria>actinobacteria                        Dermacoccus sp. Ellin185                                                     conserved hypothetical protein [Dermacoccus sp. Ellin185].                                                                     <-309812170_?<-309812122_DoxX||309812198_?->309812141_SRAP*->309812164_abhydrolase->309812224_Sigma->309812163_?->       317125651    <-Sigma<-abhydrolase<-SRAP*||DoxX->                                        -                               280   bacteria>actinobacteria                        Intrasporangium calvum DSM 43043                                             hypothetical protein Intca_2530 [Intrasporangium calvum DSM 43043].                                                            <-317125648_?<-317125649_Sigma<-317125650_abhydrolase<-317125651_SRAP*||317125652_DoxX->317125653_?->317125654_?->       406575234    <-DoxX||SRAP*->abhydrolase->                                               B277_10740                      211   bacteria>actinobacteria                        Janibacter hoylei PVAS-1                                                     hypothetical protein B277_10740 [Janibacter hoylei PVAS-1].                                                                    <-406575231_?<-406575232_?<-406575233_DoxX||406575234_SRAP*->406575235_abhydrolase-><-406575236_?<-406575237_?       84494572     <-Sigma||?-><-abhydrolase<-SRAP*||DoxX->                                   -                               280   bacteria>actinobacteria                        Janibacter sp. HTCC2649                                                      hypothetical protein JNB_07239 [Janibacter sp. HTCC2649].                                                                      <-84494569_Sigma||84494570_?-><-84494571_abhydrolase<-84494572_SRAP*||84494573_DoxX->84494574_?->84494575_?->       357391811    SRAP*->abhydrolase->Sigma->                                                -                               245   bacteria>actinobacteria                        Kitasatospora setae KM-6054                                                  hypothetical protein KSE_49180 [Kitasatospora setae KM-6054].                                                                  <-357391808_?<-357391809_?<-357391810_?||357391811_SRAP*->357391812_abhydrolase->357391813_Sigma->357391814_?->       284033101    <-Sigma<-abhydrolase<-SRAP*                                                -                               269   bacteria>actinobacteria                        Kribbella flavida DSM 17836                                                  hypothetical protein Kfla_5218 [Kribbella flavida DSM 17836].                                                                  <-284033098_?<-284033099_Sigma<-284033100_abhydrolase<-284033101_SRAP*<-284033102_?||284033103_?->284033104_?->       336118739    <-Sigma<-abhydrolase<-SRAP*<-?<-?||DoxX->                                  -                               273   bacteria>actinobacteria                        Microlunatus phosphovorus NM-1                                               hypothetical protein MLP_30940 [Microlunatus phosphovorus NM-1].                                                               <-336118736_?<-336118737_Sigma<-336118738_abhydrolase<-336118739_SRAP*<-336118740_?<-336118741_?||336118742_DoxX->       119715939    <-DoxX<-?||?->SRAP*->abhydrolase->Sigma->                                  -                               253   bacteria>actinobacteria                        Nocardioides sp. JS614                                                       hypothetical protein Noca_1704 [Nocardioides sp. JS614].                                                                       <-119715936_DoxX<-119715937_?||119715938_?->119715939_SRAP*->119715940_abhydrolase->119715941_Sigma->119715942_?->       331698911    aminoacyl-tRNA_deacylase-><-?<-abhydrolase<-SRAP*                          -                               270   bacteria>actinobacteria                        Pseudonocardia dioxanivorans CB1190                                          hypothetical protein Psed_5160 [Pseudonocardia dioxanivorans CB1190].                                                          331698908_aminoacyl-tRNA_deacylase-><-331698909_?<-331698910_abhydrolase<-331698911_SRAP*||331698912_?->331698913_?->331698914_?->       257057046    Sigma->?-><-abhydrolase<-SRAP*                                             -                               268   bacteria>actinobacteria                        Saccharomonospora viridis DSM 43017                                          hypothetical protein Svir_30760 [Saccharomonospora viridis DSM 43017].                                                         257057043_Sigma->257057044_?-><-257057045_abhydrolase<-257057046_SRAP*||257057047_?->257057048_?->257057049_?->       348168918    SRAP*->SRAP->abhydrolase-><-aminoacyl-tRNA_deacylase||Sigma->              SspiN1_010100000045             254   bacteria>actinobacteria                        Saccharopolyspora spinosa NRRL 18395                                         putative bacteriophage protein [Saccharopolyspora spinosa NRRL 18395].                                                         <-348168915_?<-348168916_?<-348168917_?||348168918_SRAP*->348168919_SRAP->348168920_abhydrolase-><-348168921_aminoacyl-tRNA_deacylase||348168922_Sigma->       145596229    <-Sigma<-abhydrolase<-?<-SRAP*                                             -                               242   bacteria>actinobacteria                        Salinispora tropica CNB-440                                                  hypothetical protein Strop_3717 [Salinispora tropica CNB-440].                                                                 <-145596226_Sigma<-145596227_abhydrolase<-145596228_?<-145596229_SRAP*||145596230_?->145596231_?->145596232_?->       440705367    SRAP*->abhydrolase->Sigma->                                                STRTUCAR8_07336                 318   bacteria>actinobacteria                        Streptomyces turgidiscabies Car8                                             PF02586 family protein [Streptomyces turgidiscabies Car8].                                                                     <-440705366_?<-440705364_?<-440705357_?||440705367_SRAP*->440705356_abhydrolase->440705361_Sigma->440705365_?->       478758661    <-DoxX||SRAP*->abhydrolase->Sigma->                                        BN10_1730023                    283   bacteria>actinobacteria                        Tetrasphaera elongata Lp2                                                    conserved hypothetical protein [Tetrasphaera elongata Lp2].                                                                    <-478758658_?<-478758659_?<-478758660_DoxX||478758661_SRAP*->478758662_abhydrolase->478758663_Sigma-><-478758664_?       406663315    abhydrolase->SRAP*->                                                       B879_03405                      233   bacteria>bacteroidetes                         Cecembia lonarensis LW9                                                      hypothetical protein B879_03405 [Cecembia lonarensis LW9].                                                                     <-406663312_?<-406663313_?||406663314_abhydrolase->406663315_SRAP*->406663316_?->406663317_?->406663318_?->       110638263    abhydrolase->SRAP*->                                                       -                               232   bacteria>bacteroidetes                         Cytophaga hutchinsonii ATCC 33406                                            hypothetical protein CHU_1864 [Cytophaga hutchinsonii ATCC 33406].                                                             <-110638260_?<-110638261_?||110638262_abhydrolase->110638263_SRAP*->110638264_?-><-110638265_?||110638266_?->       431799206    abhydrolase->SRAP*->                                                       -                               232   bacteria>bacteroidetes                         Echinicola vietnamensis DSM 17526                                            hypothetical protein Echvi_3888 [Echinicola vietnamensis DSM 17526].                                                           <-431799203_?<-431799204_?||431799205_abhydrolase->431799206_SRAP*->431799207_?->431799208_?->431799209_?->       441501428    abhydrolase->SRAP*->                                                       C900_00610                      231   bacteria>bacteroidetes                         Fulvivirga imtechensis AK7                                                   hypothetical protein C900_00610 [Fulvivirga imtechensis AK7].                                                                  <-441501425_?<-441501426_?||441501427_abhydrolase->441501428_SRAP*->441501429_?->441501430_?->441501431_?->       390444946    abhydrolase->SRAP*->                                                       A3SI_14399                      232   bacteria>bacteroidetes                         Nitritalea halalkaliphila LW7                                                hypothetical protein A3SI_14399 [Nitritalea halalkaliphila LW7].                                                               <-390444943_?<-390444944_?||390444945_abhydrolase->390444946_SRAP*->390444947_?->390444948_?->390444949_?->       254446224    abhydrolase-><-SRAP*                                                       VDG1235_4473                    244   bacteria>verrucomicrobia                       Verrucomicrobiae bacterium DG1235                                            conserved hypothetical protein [Verrucomicrobiae bacterium DG1235].                                                            <-254442551_?||254442176_?->254442879_abhydrolase-><-254446224_SRAP*<-254442225_?<-254446556_?||254445609_?->        # LexA associated       254431381    <-LexA<-?<-Metallopeptidase||SRAP*->                                       CPCC7001_1272                   215   bacteria>cyanobacteria                         Cyanobium sp. PCC 7001                                                       conserved hypothetical protein [Cyanobium sp. PCC 7001].                                                                       <-254432149_LexA<-254431613_?<-254430431_Metallopeptidase||254431381_SRAP*->254430962_?->254430391_?-><-254431638_?       33862650     <-SRAP*||Metallopeptidase->?->LexA->                                       -                               218   bacteria>cyanobacteria                         Prochlorococcus marinus str. MIT 9313                                        hypothetical protein PMT0377 [Prochlorococcus marinus str. MIT 9313].                                                          33862647_?->33862648_?-><-33862649_?<-33862650_SRAP*||33862651_Metallopeptidase->33862652_?->33862653_LexA->       116072383    <-SRAP*||?->?->LexA->                                                      -                               215   bacteria>cyanobacteria                         Synechococcus sp. BL107                                                      hypothetical protein BL107_11366 [Synechococcus sp. BL107].                                                                    116072380_?->116072381_?-><-116072382_?<-116072383_SRAP*||116072384_?->116072385_?->116072386_LexA->       78185051     <-LexA<-?<-?||SRAP*->                                                      Syncc9902_1484                  215   bacteria>cyanobacteria                         Synechococcus sp. CC9902                                                     hypothetical protein Syncc9902_1484 [Synechococcus sp. CC9902].                                                                <-78185048_LexA<-78185049_?<-78185050_?||78185051_SRAP*->78185052_?-><-78185053_?<-78185054_?       317969091    <-LexA<-?<-?||SRAP*->                                                      SCB02_010100006120              215   bacteria>cyanobacteria                         Synechococcus sp. CB0205                                                     hypothetical protein SCB02_06120 [Synechococcus sp. CB0205].                                                                   <-317969088_LexA<-317969089_?<-317969090_?||317969091_SRAP*->317969092_?-><-317969093_?<-317969094_?       148242852    <-SRAP*||Metallopeptidase->?->LexA->                                       SynRCC307_1753                  220   bacteria>cyanobacteria                         Synechococcus sp. RCC307                                                     hypothetical protein SynRCC307_1753 [Synechococcus sp. RCC307].                                                                148242849_?->148242850_?-><-148242851_?<-148242852_SRAP*||148242853_Metallopeptidase->148242854_?->148242855_LexA->       87301140     <-SRAP*||?->?->LexA->                                                      -                               210   bacteria>cyanobacteria                         Synechococcus sp. WH 5701                                                    hypothetical protein WH5701_14681 [Synechococcus sp. WH 5701].                                                                 87301137_?-><-87301138_?<-87301139_?<-87301140_SRAP*||87301141_?->87301142_?->87301143_LexA->       398814251    LexA-><-?||?-><-SRAP*                                                      PMI05_01344                     229   bacteria>firmicutes                            Brevibacillus sp. BC25                                                       hypothetical protein PMI05_01344 [Brevibacillus sp. BC25].                                                                     398814248_LexA-><-398814249_?||398814250_?-><-398814251_SRAP*<-398814252_?<-398814253_?||398814254_?->       223986047    <-SRAP*<-ImuBC||LexA->                                                     HOLDEFILI_03382                 192   bacteria>firmicutes                            Holdemania filiformis DSM 12042                                              hypothetical protein HOLDEFILI_03382 [Holdemania filiformis DSM 12042].                                                        <-223986044_?||223986045_?->223986046_?-><-223986047_SRAP*<-223986048_ImuBC||223986049_LexA->223986050_?->       406830325    SRAP*-><-?||LexA->                                                         SpalD1_010100001759             131   bacteria>planctomycetes                        Schlesneria paludicola DSM 18645                                             hypothetical protein SpalD1_01759, partial [Schlesneria paludicola DSM 18645].                                                 <-406830322_?<-406830323_?||406830324_?->406830325_SRAP*-><-406830326_?||406830327_LexA->406830328_?->       365090507    <-LexA<-?||SRAP*->                                                         KYG_07131                       217   bacteria>proteobacteria>betaproteobacteria     Acidovorax sp. NO-1                                                          hypothetical protein KYG_07131 [Acidovorax sp. NO-1].                                                                          <-365090504_?<-365090505_LexA<-365090506_?||365090507_SRAP*-><-365090508_?<-365090509_?<-365090510_?       118578633    RecD->?->?-><-SRAP*<-DinB<-ImuBC<-LexA                                     -                               238   bacteria>proteobacteria>deltaproteobacteria    Pelobacter propionicus DSM 2379                                              hypothetical protein Ppro_0189 [Pelobacter propionicus DSM 2379].                                                              118578630_RecD->118578631_?->118578632_?-><-118578633_SRAP*<-118578634_DinB<-118578635_ImuBC<-118578636_LexA       39995151     SRAP*->LexA->ImuBC->DinB->                                                 -                               223   bacteria>proteobacteria>deltaproteobacteria    Geobacter sulfurreducens PCA                                                 hypothetical protein GSU0040 [Geobacter sulfurreducens PCA].                                                                   <-39995147_?||39995148_?->39995149_?->39995151_SRAP*->39995152_LexA->39995153_ImuBC->39995154_DinB->       352104018    <-LexA||?-><-?||SRAP*->                                                    HAL1_12828                      220   bacteria>proteobacteria>gammaproteobacteria    Halomonas sp. HAL1                                                           hypothetical protein HAL1_12828 [Halomonas sp. HAL1].                                                                          <-352104015_LexA||352104016_?-><-352104017_?||352104018_SRAP*-><-352104019_?<-352104020_?<-352104021_?       374262520    SRAP*->LexA->                                                              LDG_7504                        230   bacteria>proteobacteria>gammaproteobacteria    Legionella drancourtii LLAP12                                                hypothetical protein LDG_7504 [Legionella drancourtii LLAP12].                                                                 374262517_?-><-374262518_?<-374262519_?||374262520_SRAP*->374262521_LexA->374262522_?-><-374262523_?       469817936    SRAP*-><-?<-?||LexA->                                                      -                               329   bacteria>proteobacteria>gammaproteobacteria    Rhodanobacter sp. 2APBS1                                                     hypothetical protein R2APBS1_1653 [Rhodanobacter sp. 2APBS1].                                                                  469817933_?->469817934_?->469817935_?->469817936_SRAP*-><-469817937_?<-469817938_?||469817939_LexA->       344339114    SRAP*-><-?<-LexA                                                           ThimaDRAFT_1782                 230   bacteria>proteobacteria>gammaproteobacteria    Thiocapsa marina 5811                                                        protein of unknown function DUF159 [Thiocapsa marina 5811].                                                                    <-344339111_?<-344339112_?||344339113_?->344339114_SRAP*-><-344339115_?<-344339116_LexA<-344339117_?       381156558    LexA->RecD->SRAP*->                                                        Thi970DRAFT_00117               238   bacteria>proteobacteria>gammaproteobacteria    Thiorhodovibrio sp. 970                                                      hypothetical protein Thi970DRAFT_00117 [Thiorhodovibrio sp. 970].                                                              <-381156555_?||381156556_LexA->381156557_RecD->381156558_SRAP*-><-381156559_?<-381156560_?||381156561_?->        # Other DinB-associated       158338582    <-RecA||?->SRAP*->DinB->                                                   -                               216   bacteria>cyanobacteria                         Acaryochloris marina MBIC11017                                               hypothetical protein AM1_5485 [Acaryochloris marina MBIC11017].                                                                158338579_?-><-158338580_RecA||158338581_?->158338582_SRAP*->158338583_DinB->158338584_?-><-158338585_?       154503607    DinB->ImuBC->SRAP*->                                                       RUMGNA_01431                    202   bacteria>firmicutes                            Ruminococcus gnavus ATCC 29149                                               hypothetical protein RUMGNA_01431 [Ruminococcus gnavus ATCC 29149].                                                            <-154503604_?||154503605_DinB->154503606_ImuBC->154503607_SRAP*-><-154503608_?<-154503609_?<-154503610_?       160882042    <-DinB<-?<-ImuBC||SRAP*->                                                  -                               220   bacteria>firmicutes                            Clostridium phytofermentans ISDg                                             hypothetical protein Cphy_3924 [Clostridium phytofermentans ISDg].                                                             <-160882039_DinB<-160882040_?<-160882041_ImuBC||160882042_SRAP*->160882043_?-><-160882044_?<-160882045_?       154498604    <-ImuBC<-DinB||SRAP*->                                                     BACCAP_02594                    193   bacteria>firmicutes                            Pseudoflavonifractor capillosus ATCC 29799                                   hypothetical protein BACCAP_02594 [Pseudoflavonifractor capillosus ATCC 29799].                                                154498601_?-><-154498602_ImuBC<-154498603_DinB||154498604_SRAP*-><-154498605_?<-154498607_?||154498606_?->       160933264    DinB->ImuBC->SRAP*->                                                       CLOLEP_02109                    110   bacteria>firmicutes                            Clostridium leptum DSM 753                                                   hypothetical protein CLOLEP_02109 [Clostridium leptum DSM 753].                                                                160933262_DinB->160933263_ImuBC->160933264_SRAP*->160933265_?-><-160933266_?||160933267_?->       169334029    <-ImuBC<-DinB||SRAP*->                                                     ANASTE_00422                    194   bacteria>firmicutes                            Anaerofustis stercorihominis DSM 17244                                       hypothetical protein ANASTE_00422 [Anaerofustis stercorihominis DSM 17244].                                                    169334026_?-><-169334027_ImuBC<-169334028_DinB||169334029_SRAP*-><-169334030_?<-169334031_?<-169334032_?       410657807    ImuBC->DinB-><-SRAP*                                                       DHBDCA_p1165                    227   bacteria>firmicutes                            Dehalobacter sp. DCA                                                         hypothetical protein DHBDCA_p1165 [Dehalobacter sp. DCA].                                                                      <-410657804_?||410657805_ImuBC->410657806_DinB-><-410657807_SRAP*<-410657808_?||410657809_?->410657810_?->       225568823    <-ImuBC<-DinB<-SRAP*                                                       -                               192   bacteria>firmicutes                            Clostridium hylemonae DSM 15053                                              hypothetical protein CLOHYLEM_04902 [Clostridium hylemonae DSM 15053].                                                         <-225568820_?<-225568821_ImuBC<-225568822_DinB<-225568823_SRAP*||225568824_?-><-225568825_?<-225568826_?       323485052    DinB->SRAP*->                                                              HMPREF9474_02156                197   bacteria>firmicutes                            Clostridium symbiosum WAL-14163                                              hypothetical protein HMPREF9474_02156 [Clostridium symbiosum WAL-14163].                                                       323485049_?->323485050_?->323485051_DinB->323485052_SRAP*->323485053_?->323485054_?->323485055_?->       326201829    <-SRAP*<-ImuBC<-DinB                                                       Cpap_2871                       206   bacteria>firmicutes                            Clostridium papyrosolvens DSM 2782                                           protein of unknown function DUF159 [Clostridium papyrosolvens DSM 2782].                                                       326201828_?-><-326201829_SRAP*<-326201830_ImuBC<-326201831_DinB||326201832_?->       336427048    DinB->ImuBC-><-SRAP*                                                       HMPREF0994_03059                194   bacteria>firmicutes                            Lachnospiraceae bacterium 3_1_57FAA_CT1                                      hypothetical protein HMPREF0994_03059 [Lachnospiraceae bacterium 3_1_57FAA_CT1].                                               <-336427045_?||336427046_DinB->336427047_ImuBC-><-336427048_SRAP*<-336427049_?<-336427050_?<-336427051_?       160931760    DinB->ImuBC->SRAP*->                                                       CLOLEP_00591                    187   bacteria>firmicutes                            Clostridium leptum DSM 753                                                   hypothetical protein CLOLEP_00591 [Clostridium leptum DSM 753].                                                                160931757_?->160931758_DinB->160931759_ImuBC->160931760_SRAP*-><-160931761_?||160931762_?->160931763_?->       358064251    <-SRAP*<-ImuBC<-DinB                                                       -                               208   bacteria>firmicutes                            Clostridium hathewayi WAL-18680                                              hypothetical protein HMPREF9473_02894 [Clostridium hathewayi WAL-18680].                                                       <-358064248_?<-358064249_?<-358064250_?<-358064251_SRAP*<-358064252_ImuBC<-358064253_DinB<-358064254_?       366164362    <-SRAP*<-ImuBC<-DinB                                                       AcelC_020100011886              207   bacteria>firmicutes                            Acetivibrio cellulolyticus CD2                                               hypothetical protein AcelC_11886 [Acetivibrio cellulolyticus CD2].                                                             366164359_?-><-366164360_?||366164361_?-><-366164362_SRAP*<-366164363_ImuBC<-366164364_DinB<-366164365_?       374296587    <-SRAP*<-ImuBC<-DinB                                                       -                               205   bacteria>firmicutes                            Clostridium clariflavum DSM 19732                                            hypothetical protein [Clostridium clariflavum DSM 19732].                                                                      374296584_?-><-374296585_?||374296586_?-><-374296587_SRAP*<-374296588_ImuBC<-374296589_DinB<-374296590_?       376261627    DinB->ImuBC->SRAP*->                                                       -                               206   bacteria>firmicutes                            Clostridium sp. BNL1100                                                      hypothetical protein [Clostridium sp. BNL1100].                                                                                <-376261624_?||376261625_DinB->376261626_ImuBC->376261627_SRAP*-><-376261628_?<-376261629_?<-376261630_?       479337227    <-NAD-dependent-ligase||DinB->ImuBC->SRAP*->                               -                               196   bacteria>firmicutes                            Clostridium cf. saccharolyticum K10                                          Uncharacterized conserved protein [Clostridium cf. saccharolyticum K10].                                                       <-479337224_NAD-dependent-ligase||479337225_DinB->479337226_ImuBC->479337227_SRAP*->479337228_?->479337229_?->479337230_?->       417305145    SRAP*->DinB->                                                              RBWH47_01016                    306   bacteria>planctomycetes                        Rhodopirellula baltica WH47                                                  protein containing DUF159 [Rhodopirellula baltica WH47].                                                                       417305142_?-><-417305143_?<-417305144_?||417305145_SRAP*->417305146_DinB->417305147_?-><-417305148_?       13488544     <-DinB<-SRAP||SRAP*->                                                      mlr9712                         196   bacteria>proteobacteria>alphaproteobacteria    Mesorhizobium loti MAFF303099                                                hypothetical protein mlr9712 [Mesorhizobium loti MAFF303099].                                                                  13488540_?->13488541_?-><-13488542_DinB<-13488543_SRAP||13488544_SRAP*-><-13488545_?||13488546_?->13488547_?->       146301312    DinB->DnaE2->SRAP*->                                                       -                               208   bacteria>bacteroidetes                         Flavobacterium johnsoniae UW101                                              hypothetical protein Fjoh_3570 [Flavobacterium johnsoniae UW101].                                                              <-146301309_?||146301310_DinB->146301311_DnaE2->146301312_SRAP*->146301313_?-><-146301314_?<-146301315_?       344203866    CLP_protease->?-><-SRAP*||DinB->DnaE2->                                    -                               252   bacteria>bacteroidetes                         Muricauda ruestringensis DSM 13258                                           hypothetical protein [Muricauda ruestringensis DSM 13258].                                                                     344203863_?->344203864_CLP_protease->344203865_?-><-344203866_SRAP*||344203867_DinB->344203868_DnaE2->344203869_?->       443243089    <-DUF4130<-SplB-photolyase<-DnaE2<-DinB<-?<-SRAP*||LexA->                  -                               275   bacteria>bacteroidetes                         Nonlabens dokdonensis DSW-6                                                  YoaM [Nonlabens dokdonensis DSW-6].                                                                                            <-443243084_DUF4130<-443243085_SplB-photolyase<-443243086_DnaE2<-443243087_DinB<-443243088_?<-443243089_SRAP*||443243090_LexA-><-443243091_?||443243092_?->       375010696    DinB->DnaE2-><-?||SRAP*->                                                  -                               261   bacteria>bacteroidetes                         Owenweeksia hongkongensis DSM 17368                                          hypothetical protein [Owenweeksia hongkongensis DSM 17368].                                                                    375010693_DinB->375010694_DnaE2-><-375010695_?||375010696_SRAP*-><-375010697_?<-375010698_?<-375010699_?       395243328    SRAP*-><-?<-?<-DinB                                                        BN55_07115                      204   bacteria>firmicutes                            Lactobacillus hominis CRBIP 24.179                                           Putative uncharacterized protein [Lactobacillus hominis CRBIP 24.179].                                                         <-395243325_?<-395243326_?<-395243327_?||395243328_SRAP*-><-395243329_?<-395243330_?<-395243331_DinB       220932161    <-SRAP*<-DinB                                                              -                               197   bacteria>firmicutes                            Halothermothrix orenii H 168                                                 hypothetical protein Hore_13240 [Halothermothrix orenii H 168].                                                                <-220932158_?<-220932159_?<-220932160_?<-220932161_SRAP*<-220932162_DinB<-220932163_?<-220932164_?       190606546    <-Resolvase<-SRAP*<-?<-DinB                                                -pVEF3_p62                      201   bacteria>firmicutes                            Enterococcus faecium                                                         hypothetical protein -pVEF3_p62 [Enterococcus faecium].                                                                        190606543_?-><-190606544_?<-190606545_Resolvase<-190606546_SRAP*<-190606547_?<-190606548_DinB<-190606549_?       257868687    <-SRAP*<-DinB                                                              EGBG_00275                      193   bacteria>firmicutes                            Enterococcus gallinarum EG2                                                  conserved hypothetical protein [Enterococcus gallinarum EG2].                                                                  257868684_?-><-257868685_?||257868686_?-><-257868687_SRAP*<-257868688_DinB<-257868690_?||257868689_?->       386284370    UDG->SRAP*-><-LexA||DinB->                                                 SULAR_03957                     225   bacteria>proteobacteria>epsilonproteobacteria  Sulfurovum sp. AR                                                            hypothetical protein SULAR_03957 [Sulfurovum sp. AR].                                                                          <-386284367_?<-386284368_?||386284369_UDG->386284370_SRAP*-><-386284371_LexA||386284372_DinB-><-386284373_?       325571863    DinB->?->SRAP*-><-?||?-><-ClpB                                             HMPREF9087_3474                 196   bacteria>firmicutes                            Enterococcus casseliflavus ATCC 12755                                        hypothetical protein HMPREF9087_3474 [Enterococcus casseliflavus ATCC 12755].                                                  <-325571829_?||325571861_DinB->325571862_?->325571863_SRAP*-><-325571864_?||325571865_?-><-325571866_ClpB       69246177     <-Resolvase||?-><-SRAP*<-?<-DinB                                           EfaeDRAFT_1518                  196   bacteria>firmicutes                            Enterococcus faecium DO                                                      Protein of unknown function DUF159 [Enterococcus faecium DO].                                                                  <-69246174_?<-69246175_Resolvase||69246176_?-><-69246177_SRAP*<-69246178_?<-69246179_DinB||69246180_?->       268611913    <-DinB<-?<-?<-SRAP*                                                        RflaF_010100020701              228   bacteria>firmicutes                            Ruminococcus flavefaciens FD-1                                               hypothetical protein RflaF_20701 [Ruminococcus flavefaciens FD-1].                                                             <-268611910_DinB<-268611911_?<-268611912_?<-268611913_SRAP*<-268611914_?<-268611915_?<-268611916_?       317133846    <-DinB<-?<-SRAP*                                                           Rumal_3418                      228   bacteria>firmicutes                            Ruminococcus albus 7                                                         protein of unknown function DUF159 [Ruminococcus albus 7].                                                                     <-317133843_?<-317133844_DinB<-317133845_?<-317133846_SRAP*<-317133847_?<-317133848_?<-317133849_?       421610151    <-DinB<-SRAP*                                                              RBSH_01133                      306   bacteria>planctomycetes                        Rhodopirellula baltica SH28                                                  protein containing DUF159 [Rhodopirellula baltica SH28].                                                                       421610148_?->421610149_?-><-421610150_DinB<-421610151_SRAP*||421610152_?->421610153_?->421610154_?->       470093668    <-DinB<-SRAP*                                                              RESH_03312                      261   bacteria>planctomycetes                        Rhodopirellula europaea SH398                                                protein containing DUF159 [Rhodopirellula europaea SH398].                                                                     470093665_?->470093666_?-><-470093667_DinB<-470093668_SRAP*       440713168    <-DinB<-SRAP*                                                              RBSWK_00827                     260   bacteria>planctomycetes                        Rhodopirellula baltica SWK14                                                 protein containing DUF159 [Rhodopirellula baltica SWK14].                                                                      440713165_?->440713166_?-><-440713167_DinB<-440713168_SRAP*||440713169_?->440713170_?->440713171_?->       449137082    <-DinB<-SRAP*                                                              RE6C_04468                      260   bacteria>planctomycetes                        Rhodopirellula europaea 6C                                                   protein containing DUF159 [Rhodopirellula europaea 6C].                                                                        449137079_?->449137080_?-><-449137081_DinB<-449137082_SRAP*<-449137083_?||449137084_?->449137085_?->       32472974     SRAP*->DinB->                                                              -                               283   bacteria>planctomycetes                        Rhodopirellula baltica SH 1                                                  hypothetical protein RB4223 [Rhodopirellula baltica SH 1].                                                                     <-32472971_?||32472972_?-><-32472973_?||32472974_SRAP*->32472975_DinB->32472976_?->32472977_?->       414177213    <-DinB<-SRAP||SRAP*-><-?<-ATP-dependent-ligase                             HMPREF9695_04971                196   bacteria>proteobacteria>alphaproteobacteria    Afipia broomeae ATCC 49717                                                   hypothetical protein HMPREF9695_04971 [Afipia broomeae ATCC 49717].                                                            <-414177209_?<-414177210_?<-414177211_DinB<-414177212_SRAP||414177213_SRAP*-><-414177214_?<-414177215_ATP-dependent-ligase<-414177216_?       398356291    <-SRAP*||SRAP-><-DinB                                                      USDA257_p02030                  196   bacteria>proteobacteria>alphaproteobacteria    Sinorhizobium fredii USDA 257                                                hypothetical protein USDA257_p02030 [Sinorhizobium fredii USDA 257].                                                           398356288_?-><-398356289_?||398356290_?-><-398356291_SRAP*||398356292_SRAP-><-398356293_DinB||398356294_?->398356295_?->       398353764    <-DinB<-SNase<-SRAP*                                                       USDA257_c39270                  220   bacteria>proteobacteria>alphaproteobacteria    Sinorhizobium fredii USDA 257                                                hypothetical protein USDA257_c39270 [Sinorhizobium fredii USDA 257].                                                           398353761_?-><-398353762_DinB<-398353763_SNase<-398353764_SRAP*<-398353765_?<-398353766_?||398353767_?->       407690786    <-SRAP*<-?<-?<-DinB                                                        BN406_04281                     196   bacteria>proteobacteria>alphaproteobacteria    Sinorhizobium meliloti Rm41                                                  hypothetical protein BN406_04281 [Sinorhizobium meliloti Rm41].                                                                <-407690783_?<-407690784_?<-407690785_?<-407690786_SRAP*<-407690787_?<-407690788_?<-407690789_DinB       16262936     <-SRAP*||SRAP-><-DinB<-DinB                                                SMa0882                         196   bacteria>proteobacteria>alphaproteobacteria    Sinorhizobium meliloti 1021                                                  hypothetical protein SMa0882 [Sinorhizobium meliloti 1021].                                                                    <-16262933_?<-16262934_?<-16262935_?<-16262936_SRAP*||16262937_SRAP-><-16262938_DinB<-193782614_DinB||16262939_?->       355629576    <-SRAP*<-DinB                                                              HMPREF1020_04518                197   bacteria>firmicutes                            Clostridium sp. 7_3_54FAA                                                    hypothetical protein HMPREF1020_04518 [Clostridium sp. 7_3_54FAA].                                                             <-355629573_?<-355629574_?<-355629575_?<-355629576_SRAP*<-355629577_DinB<-355629578_?<-355629579_?       346308553    <-NAD-dependent-ligase||DinB->ImuB-C->SRAP*->                              HMPREF9457_02373                195   bacteria>firmicutes                            Dorea formicigenerans 4_6_53AFAA                                             hypothetical protein HMPREF9457_02373 [Dorea formicigenerans 4_6_53AFAA].                                                      <-346308550_NAD-dependent-ligase||346308551_DinB->346308552_ImuB-C->346308553_SRAP*-><-346308554_?<-346308555_?<-346308556_?       431278656    <-SRAP*<-?<-DinB                                                           OIO_05517                       196   bacteria>firmicutes                            Enterococcus faecium E1623                                                   hypothetical protein OIO_05517 [Enterococcus faecium E1623].                                                                   431278655_?-><-431278656_SRAP*<-431278657_?<-431278658_DinB       85858878     <-DinB<-?||SRAP*->                                                         -                               207   bacteria>proteobacteria>deltaproteobacteria    Syntrophus aciditrophicus SB                                                 cytoplasmic protein [Syntrophus aciditrophicus SB].                                                                            85858875_?-><-85858876_DinB<-85858877_?||85858878_SRAP*->85858879_?-><-85858880_?||85858881_?->        # Other Family Y DNA polymerase associated       444351103    Family_Y_DNApol-><-?||?-><-SRAP*                                           ST548_p7351                     225   bacteria>proteobacteria>gammaproteobacteria    Enterobacter aerogenes EA1509E                                               Gifsy-2 prophage protein [Enterobacter aerogenes EA1509E].                                                                     444351100_Family_Y_DNApol-><-444351101_?||444351102_?-><-444351103_SRAP*<-444351104_?<-444351105_?<-444351106_?        # UvrD associated       390955816    UvrD->SRAP*->                                                              Aeqsu_3124                      238   bacteria>bacteroidetes                         Aequorivita sublithincola DSM 14238                                          hypothetical protein Aeqsu_3124 [Aequorivita sublithincola DSM 14238].                                                         <-390955813_?<-390955814_?||390955815_UvrD->390955816_SRAP*->390955817_?->390955818_?->390955819_?->       323139777    UvrD->SRAP*->                                                              Met49242DRAFT_4201              111   bacteria>proteobacteria>alphaproteobacteria    Methylocystis sp. ATCC 49242                                                 protein of unknown function DUF159, partial [Methylocystis sp. ATCC 49242].                                                    323139774_?->323139775_?->323139776_UvrD->323139777_SRAP*->       323139768    UvrD->SRAP*->?-><-DNA_pol_B_epsi                                           Met49242DRAFT_4192              221   bacteria>proteobacteria>alphaproteobacteria    Methylocystis sp. ATCC 49242                                                 protein of unknown function DUF159 [Methylocystis sp. ATCC 49242].                                                             <-323139765_?<-323139766_?||323139767_UvrD->323139768_SRAP*->323139769_?-><-323139770_DNA_pol_B_epsi||323139771_?->       323139147    UvrD->SRAP*->SRAP->                                                        Met49242DRAFT_3592              142   bacteria>proteobacteria>alphaproteobacteria    Methylocystis sp. ATCC 49242                                                 protein of unknown function DUF159 [Methylocystis sp. ATCC 49242].                                                             323139144_?->323139145_?->323139146_UvrD->323139147_SRAP*->323139148_SRAP->323139149_?->323139150_?-><-323139151_?        # ATP-dependent ligase associated       77454673     ATP-dependent-ligase->?->SRAP*->                                           pREL1_0106                      240   bacteria>actinobacteria                        Rhodococcus erythropolis PR4                                                 hypothetical protein pREL1_0106 [Rhodococcus erythropolis PR4].                                                                77454670_?->77454671_ATP-dependent-ligase->77454672_?->77454673_SRAP*->77454674_?-><-77454675_?<-77454676_?       111025281    ATP-dependent-ligase-><-SRAP*                                              RHA1_ro08499                    250   bacteria>actinobacteria                        Rhodococcus jostii RHA1                                                      hypothetical protein RHA1_ro08499 [Rhodococcus jostii RHA1].                                                                   <-111025278_?||111025279_?->111025280_ATP-dependent-ligase-><-111025281_SRAP*<-111025282_?<-111025283_?||111025284_?->       255535037    <-SRAP*||ATP-dependent-ligase->                                            FIC_00894                       210   bacteria>bacteroidetes                         Flavobacteriaceae bacterium 3519-10                                          hypothetical protein FIC_00894 [Flavobacteriaceae bacterium 3519-10].                                                          255535034_?->255535035_?->255535036_?-><-255535037_SRAP*||255535038_ATP-dependent-ligase-><-255535039_?<-255535040_?       398306655    <-ATP-dependent-ligase<-SRAP*                                              BvalD_010100014740              224   bacteria>firmicutes                            Bacillus vallismortis DV1-F-3                                                hypothetical protein BvalD_14740 [Bacillus vallismortis DV1-F-3].                                                              398306652_?->398306653_?-><-398306654_ATP-dependent-ligase<-398306655_SRAP*||398306656_?->398306657_?->398306658_?->       387898405    <-ATP-dependent-ligase<-SRAP*                                              -                               227   bacteria>firmicutes                            Bacillus amyloliquefaciens Y2                                                hypothetical protein MUS_2009 [Bacillus amyloliquefaciens Y2].                                                                 387898402_?->387898403_?-><-387898404_ATP-dependent-ligase<-387898405_SRAP*||387898406_?->387898407_?->387898408_?->       384265419    <-ATP-dependent-ligase<-SRAP*                                              -                               224   bacteria>firmicutes                            Bacillus amyloliquefaciens subsp. plantarum YAU B9601-Y2                     hypothetical protein BANAU_1789 [Bacillus amyloliquefaciens subsp. plantarum YAU B9601-Y2].                                    384265416_?->384265417_?-><-384265418_ATP-dependent-ligase<-384265419_SRAP*||384265420_?->384265421_?->384265422_?->       384158911    <-ATP-dependent-ligase<-SRAP*                                              -                               224   bacteria>firmicutes                            Bacillus amyloliquefaciens TA208                                             hypothetical protein BAMTA208_06580 [Bacillus amyloliquefaciens TA208].                                                        384158908_?->384158909_?-><-384158910_ATP-dependent-ligase<-384158911_SRAP*||384158912_?->384158913_?->384158914_?->       418032788    <-ATP-dependent-ligase<-SRAP*                                              BSSC8_22140                     191   bacteria>firmicutes                            Bacillus subtilis subsp. subtilis str. SC-8                                  hypothetical protein BSSC8_22140 [Bacillus subtilis subsp. subtilis str. SC-8].                                                418032785_?->418032786_?-><-418032787_ATP-dependent-ligase<-418032788_SRAP*||418032789_?->418032790_?->418032791_?->       335035309    SRAP*->?-><-?<-ATP-dependent-ligase                                        AGRO_2641                       338   bacteria>proteobacteria>alphaproteobacteria    Agrobacterium sp. ATCC 31749                                                 hypothetical protein AGRO_2641 [Agrobacterium sp. ATCC 31749].                                                                 335035306_?-><-335035308_?||335035307_?->335035309_SRAP*->335035310_?-><-335035311_?<-335035312_ATP-dependent-ligase       367474288    <-ATP-dependent-ligase<-SRAP*                                              BRAO285_1800025                 118   bacteria>proteobacteria>alphaproteobacteria    Bradyrhizobium sp. ORS 285                                                   hypothetical protein BRAO285_1800025 [Bradyrhizobium sp. ORS 285].                                                             <-367474285_?<-367474286_?<-367474287_ATP-dependent-ligase<-367474288_SRAP*||367474289_?->       365881528    <-ATP-dependent-ligase<-SRAP*                                              BRAO375_2560034                 220   bacteria>proteobacteria>alphaproteobacteria    Bradyrhizobium sp. ORS 375                                                   conserved hypothetical protein [Bradyrhizobium sp. ORS 375].                                                                   <-365881525_?<-365881526_?<-365881527_ATP-dependent-ligase<-365881528_SRAP*<-365881529_?||365881530_?->365881531_?->       386401255    <-SRAP*<-?<-?<-ATP-dependent-ligase                                        Bra1253DRAFT_06957              225   bacteria>proteobacteria>alphaproteobacteria    Bradyrhizobium sp. WSM1253                                                   hypothetical protein Bra1253DRAFT_06957 [Bradyrhizobium sp. WSM1253].                                                          386401252_?-><-386401253_?<-386401254_?<-386401255_SRAP*<-386401256_?<-386401257_?<-386401258_ATP-dependent-ligase       374575359    SRAP*->?->?->ATP-dependent-ligase->                                        Bra471DRAFT_03982               220   bacteria>proteobacteria>alphaproteobacteria    Bradyrhizobium sp. WSM471                                                    hypothetical protein Bra471DRAFT_03982 [Bradyrhizobium sp. WSM471].                                                            <-374575356_?||374575357_?->374575358_?->374575359_SRAP*->374575360_?->374575361_?->374575362_ATP-dependent-ligase->       374365338    SRAP*-><-?||ATP-dependent-ligase->                                         OR16_04577                      218   bacteria>proteobacteria>betaproteobacteria     Cupriavidus basilensis OR16                                                  hypothetical protein OR16_04577 [Cupriavidus basilensis OR16].                                                                 <-374365335_?||374365336_?->374365337_?->374365338_SRAP*-><-374365339_?||374365340_ATP-dependent-ligase->374365341_?->       421745210    ATP-dependent-ligase->SRAP*->                                              B551_00290                      199   bacteria>proteobacteria>betaproteobacteria     Cupriavidus necator HPC(L)                                                   hypothetical protein B551_00290 [Cupriavidus necator HPC(L)].                                                                  421745207_?->421745208_?->421745209_ATP-dependent-ligase->421745210_SRAP*->421745211_?-><-421745212_?<-421745213_?       13476675     <-SRAP*<-ATP-dependent-ligase                                              -                               168   bacteria>proteobacteria>alphaproteobacteria    Mesorhizobium loti MAFF303099                                                hypothetical protein mll8062, partial [Mesorhizobium loti MAFF303099].                                                         13476671_?-><-13476673_?<-13476674_?<-13476675_SRAP*<-13476676_ATP-dependent-ligase<-13476677_?||13476678_?->       217979225    SRAP*->?->ATP-dependent-ligase->                                           -                               197   bacteria>proteobacteria>alphaproteobacteria    Methylocella silvestris BL2                                                  hypothetical protein Msil_3101 [Methylocella silvestris BL2].                                                                  <-217979222_?||217979223_?->217979224_?->217979225_SRAP*->217979226_?->217979227_ATP-dependent-ligase->217979228_?->       387892649    <-ATP-dependent-ligase||?->?->SRAP*->                                      -                               230   bacteria>proteobacteria>gammaproteobacteria    Pseudomonas fluorescens A506                                                 hypothetical protein PflA506_1435 [Pseudomonas fluorescens A506].                                                              <-387892646_ATP-dependent-ligase||387892647_?->387892648_?->387892649_SRAP*->387892650_?-><-387892651_?<-387892652_?       190890772    <-SRAP*<-?||?->ATP-dependent-ligase->                                      -                               250   bacteria>proteobacteria>alphaproteobacteria    Rhizobium etli CIAT 652                                                      hypothetical protein RHECIAT_CH0001155 [Rhizobium etli CIAT 652].                                                              <-190890769_?<-190890770_?<-190890771_?<-190890772_SRAP*<-190890773_?||190890774_?->190890775_ATP-dependent-ligase->       424887058    <-SRAP*<-ATP-dependent-ligase                                              Rleg10DRAFT_5112                245   bacteria>proteobacteria>alphaproteobacteria    Rhizobium leguminosarum bv. trifolii WSM2012                                 hypothetical protein Rleg10DRAFT_5112 [Rhizobium leguminosarum bv. trifolii WSM2012].                                          424887055_?->424887056_?->424887057_?-><-424887058_SRAP*<-424887059_ATP-dependent-ligase<-424887060_?<-424887061_?       430006050    <-SNase<-?<-SRAP*||?->ATP-dependent-ligase->                               NT26_4131                       241   bacteria>proteobacteria>alphaproteobacteria    Rhizobium sp.                                                                conserved hypothetical protein [Rhizobium sp.].                                                                                <-430006047_?<-430006048_SNase<-430006049_?<-430006050_SRAP*||430006051_?->430006052_ATP-dependent-ligase-><-430006053_?       430004191    <-ATP-dependent-ligase<-?||?->SRAP*->                                      NT26_2258                       251   bacteria>proteobacteria>alphaproteobacteria    Rhizobium sp.                                                                conserved hypothetical protein [Rhizobium sp.].                                                                                <-430004188_ATP-dependent-ligase<-430004189_?||430004190_?->430004191_SRAP*-><-430004192_?||430004193_?->430004194_?->       420241902    <-SRAP*||ATP-dependent-ligase->                                            PMI07_03785                     222   bacteria>proteobacteria>alphaproteobacteria    Rhizobium sp. CF080                                                          hypothetical protein PMI07_03785 [Rhizobium sp. CF080].                                                                        <-420241899_?<-420241900_?<-420241901_?<-420241902_SRAP*||420241903_ATP-dependent-ligase-><-420241904_?<-420241905_?        # NAD-dependent ligase associated       340360482    <-NAD-dependent-ligase<-SRAP*                                              -                               251   bacteria>actinobacteria                        Actinomyces sp. oral taxon 448 str. F0400                                    protein of hypothetical function DUF159 [Actinomyces sp. oral taxon 448 str. F0400].                                           <-340360479_?||340360480_?-><-340360481_NAD-dependent-ligase<-340360482_SRAP*||340360483_?->340360484_?-><-340360485_?       479154887    <-NAD-dependent-ligase||SRAP*->                                            -                               195   bacteria>firmicutes                            Ruminococcus sp. SR1/5                                                       Uncharacterized conserved protein [Ruminococcus sp. SR1/5].                                                                    <-479154884_?<-479154885_?<-479154886_NAD-dependent-ligase||479154887_SRAP*-><-479154888_?<-479154889_?<-479154890_?       427390420    <-NAD-dependent-ligase||SRAP->SRAP*->                                      -                               77    bacteria>actinobacteria                        Actinobaculum massiliae ACS-171-V-Col2                                       hypothetical protein HMPREF9233_00329 [Actinobaculum massiliae ACS-171-V-Col2].                                                <-427390416_?||427390417_?-><-427390418_NAD-dependent-ligase||427390419_SRAP->427390420_SRAP*->427390421_?->427390422_?->427390423_?->       227498226    SRAP*->NAD-dependent-ligase-><-SRAP                                        HMPREF0058_2387                 258   bacteria>actinobacteria                        Actinomyces urogenitalis DSM 15434                                           protein of hypothetical function DUF159 [Actinomyces urogenitalis DSM 15434].                                                  <-227498221_?<-227498222_?<-227498225_?||227498226_SRAP*->227498227_NAD-dependent-ligase-><-227498228_SRAP||227498229_?->        # NUDIX-associated       414171802    <-NUDIX<-?||SRAP*-><-NUDIX                                                 -                               258   bacteria>proteobacteria>alphaproteobacteria    Afipia broomeae ATCC 49717                                                   hypothetical protein HMPREF9695_00359 [Afipia broomeae ATCC 49717].                                                            <-414171799_?<-414171800_NUDIX<-414171801_?||414171802_SRAP*-><-414171803_NUDIX<-414171804_?<-414171805_?       414170447    <-NUDIX<-?||SRAP*-><-NUDIX                                                 -                               252   bacteria>proteobacteria>alphaproteobacteria    Afipia clevelandensis ATCC 49720                                             hypothetical protein HMPREF9696_03888 [Afipia clevelandensis ATCC 49720].                                                      <-414170444_?<-414170445_NUDIX<-414170446_?||414170447_SRAP*-><-414170448_NUDIX<-414170449_?<-414170450_?       414163345    <-SRAP*||?->NUDIX->                                                        -                               249   bacteria>proteobacteria>alphaproteobacteria    Afipia felis ATCC 53690                                                      hypothetical protein HMPREF9697_01493 [Afipia felis ATCC 53690].                                                               <-414163342_?||414163343_?->414163344_?-><-414163345_SRAP*||414163346_?->414163347_NUDIX->414163348_?->       299134709    <-NUDIX<-?||SRAP*->                                                        AfiDRAFT_3030                   248   bacteria>proteobacteria>alphaproteobacteria    Afipia sp. 1NLS2                                                             protein of unknown function DUF159 [Afipia sp. 1NLS2].                                                                         <-299134706_?<-299134707_NUDIX<-299134708_?||299134709_SRAP*-><-299134710_?<-299134711_?||299134712_?->       408379318    <-NUDIX||SRAP*->                                                           QWE_17018                       254   bacteria>proteobacteria>alphaproteobacteria    Agrobacterium albertimagni AOL15                                             hypothetical protein QWE_17018 [Agrobacterium albertimagni AOL15].                                                             <-408379315_?<-408379316_?<-408379317_NUDIX||408379318_SRAP*-><-408379319_?<-408379320_?<-408379321_?       15888401     <-NUDIX||?->SRAP*->                                                        Atu1059                         253   bacteria>proteobacteria>alphaproteobacteria    Agrobacterium fabrum str. C58                                                conserved hypothetical protein [Agrobacterium fabrum str. C58].                                                                <-159184595_?<-15888399_NUDIX||159184596_?->15888401_SRAP*-><-159184597_?<-15888403_?<-159184598_?       222085408    <-NUDIX||?->SRAP*->                                                        -                               254   bacteria>proteobacteria>alphaproteobacteria    Agrobacterium radiobacter K84                                                hypothetical protein Arad_1617 [Agrobacterium radiobacter K84].                                                                <-222085405_?<-222085406_NUDIX||222085407_?->222085408_SRAP*-><-222085409_?<-222085410_?<-222085411_?       335036576    <-NUDIX||?->?->SRAP*->                                                     AGRO_3909                       253   bacteria>proteobacteria>alphaproteobacteria    Agrobacterium sp. ATCC 31749                                                 hypothetical protein AGRO_3909 [Agrobacterium sp. ATCC 31749].                                                                 <-335036573_NUDIX||335036574_?->335036575_?->335036576_SRAP*-><-335036577_?<-335036578_?<-335036579_?       325292438    <-NUDIX||?->SRAP*->                                                        -                               253   bacteria>proteobacteria>alphaproteobacteria    Agrobacterium sp. H13-3                                                      hypothetical protein AGROH133_05111 [Agrobacterium sp. H13-3].                                                                 <-325292435_?<-325292436_NUDIX||325292437_?->325292438_SRAP*-><-325292439_?<-325292440_?<-325292441_?       418406593    <-NUDIX||?->SRAP*->                                                        AT5A_05190                      253   bacteria>proteobacteria>alphaproteobacteria    Agrobacterium tumefaciens 5A                                                 hypothetical protein AT5A_05190 [Agrobacterium tumefaciens 5A].                                                                <-418406590_?<-418406591_NUDIX||418406592_?->418406593_SRAP*-><-418406594_?<-418406595_?<-418406596_?       418295880    <-NUDIX||?->SRAP*->                                                        ATCR1_00115                     253   bacteria>proteobacteria>alphaproteobacteria    Agrobacterium tumefaciens CCNWGS0286                                         hypothetical protein ATCR1_00115 [Agrobacterium tumefaciens CCNWGS0286].                                                       <-418295877_?<-418295878_NUDIX||418295879_?->418295880_SRAP*-><-418295881_?<-418295882_?<-418295883_?       417860506    <-SRAP*<-?||NUDIX->                                                        -                               250   bacteria>proteobacteria>alphaproteobacteria    Agrobacterium tumefaciens F2                                                 hypothetical protein Agau_C201932 [Agrobacterium tumefaciens F2].                                                              417860503_?->417860504_?->417860505_?-><-417860506_SRAP*<-417860507_?||417860508_NUDIX->417860509_?->       475898858    <-NUDIX||?->SRAP*->                                                        H009_06622                      253   bacteria>proteobacteria>alphaproteobacteria    Agrobacterium tumefaciens str. Cherry 2E-2-2                                 hypothetical protein H009_06622 [Agrobacterium tumefaciens str. Cherry 2E-2-2].                                                <-475898855_?<-475898856_NUDIX||475898857_?->475898858_SRAP*-><-475898859_?<-475898860_?<-475898861_?       222148096    <-NUDIX||SRAP*->                                                           -                               251   bacteria>proteobacteria>alphaproteobacteria    Agrobacterium vitis S4                                                       hypothetical protein Avi_1467 [Agrobacterium vitis S4].                                                                        <-222148093_?<-222148094_?<-222148095_NUDIX||222148096_SRAP*->222148097_?-><-222148098_?<-222148099_?       304392052    <-cysteinyl-tRNA_synthetase<-?<-SRAP*||NUDIX->                             -                               247   bacteria>proteobacteria>alphaproteobacteria    Ahrensia sp. R2A130                                                          protein YoaM [Ahrensia sp. R2A130].                                                                                            <-304392049_?<-304392050_cysteinyl-tRNA_synthetase<-304392051_?<-304392052_SRAP*||304392053_NUDIX->304392054_?->304392055_?->       395785999    <-NUDIX||SRAP*->cysteinyl-tRNA_synthetase->                                -                               255   bacteria>proteobacteria>alphaproteobacteria    Bartonella tamiae Th239                                                      hypothetical protein ME5_01045 [Bartonella tamiae Th239].                                                                      <-395785996_?<-395785997_?<-395785998_NUDIX||395785999_SRAP*->395786000_cysteinyl-tRNA_synthetase->395786001_?-><-395786002_?       338973353    <-NUDIX<-?||SRAP*-><-NUDIX<-?<-FAD_linked_oxidase                          -                               267   bacteria>proteobacteria>alphaproteobacteria    Bradyrhizobiaceae bacterium SG-6C                                            protein of unknown function DUF159 [Bradyrhizobiaceae bacterium SG-6C].                                                        <-338973350_?<-338973351_NUDIX<-338973352_?||338973353_SRAP*-><-338973354_NUDIX<-338973355_?<-338973356_FAD_linked_oxidase       27377675     <-NUDIX<-?||SRAP*-><-?<-?<-NUDIX                                           -                               254   bacteria>proteobacteria>alphaproteobacteria    Bradyrhizobium diazoefficiens USDA 110                                       hypothetical protein blr2564 [Bradyrhizobium diazoefficiens USDA 110].                                                         <-27377672_?<-27377673_NUDIX<-27377674_?||27377675_SRAP*-><-27377676_?<-27377677_?<-27377678_NUDIX       384220923    NUDIX->?->?-><-SRAP*||?->NUDIX->                                           -                               254   bacteria>proteobacteria>alphaproteobacteria    Bradyrhizobium japonicum USDA 6                                              hypothetical protein BJ6T_72540 [Bradyrhizobium japonicum USDA 6].                                                             384220920_NUDIX->384220921_?->384220922_?-><-384220923_SRAP*||384220924_?->384220925_NUDIX->384220926_?->       459291879    FAD_linked_oxidase->?->NUDIX-><-SRAP*||?->NUDIX->                          -                               255   bacteria>proteobacteria>alphaproteobacteria    Bradyrhizobium oligotrophicum S58                                            conserved hypothetical protein [Bradyrhizobium oligotrophicum S58].                                                            459291876_FAD_linked_oxidase->459291877_?->459291878_NUDIX-><-459291879_SRAP*||459291880_?->459291881_NUDIX->459291882_?->       148253873    <-NUDIX<-?||SRAP*-><-NUDIX                                                 -                               204   bacteria>proteobacteria>alphaproteobacteria    Bradyrhizobium sp. BTAi1                                                     hypothetical protein BBta_2383 [Bradyrhizobium sp. BTAi1].                                                                     <-148253870_?<-148253871_NUDIX<-148253872_?||148253873_SRAP*-><-148253874_NUDIX<-148253875_?<-148253876_?       421603589    <-NUDIX<-?||SRAP*-><-?<-SRAP                                               BCCGELA001_34258                254   bacteria>proteobacteria>alphaproteobacteria    Bradyrhizobium sp. CCGE-LA001                                                hypothetical protein BCCGELA001_34258 [Bradyrhizobium sp. CCGE-LA001].                                                         <-421603586_?<-421603587_NUDIX<-421603588_?||421603589_SRAP*-><-421603590_?<-421603591_SRAP       146339100    <-NUDIX<-?||SRAP*-><-NUDIX                                                 -                               204   bacteria>proteobacteria>alphaproteobacteria    Bradyrhizobium sp. ORS 278                                                   hypothetical protein BRADO2054 [Bradyrhizobium sp. ORS 278].                                                                   <-146339097_?<-146339098_NUDIX<-146339099_?||146339100_SRAP*-><-146339101_NUDIX<-146339102_?<-146339103_?       367473339    <-NUDIX<-?||SRAP*-><-NUDIX                                                 BRAO285_1410026                 204   bacteria>proteobacteria>alphaproteobacteria    Bradyrhizobium sp. ORS 285                                                   conserved hypothetical protein [Bradyrhizobium sp. ORS 285].                                                                   <-367473336_?<-367473337_NUDIX<-367473338_?||367473339_SRAP*-><-367473340_NUDIX<-367473341_?<-367473342_?       365882564    NUDIX-><-SRAP*||?->NUDIX->                                                 BRAO375_3440044                 204   bacteria>proteobacteria>alphaproteobacteria    Bradyrhizobium sp. ORS 375                                                   conserved hypothetical protein [Bradyrhizobium sp. ORS 375].                                                                   365882561_?->365882562_?->365882563_NUDIX-><-365882564_SRAP*||365882565_?->365882566_NUDIX->365882567_?->       383773659    <-SRAP*||?->NUDIX->                                                        -                               213   bacteria>proteobacteria>alphaproteobacteria    Bradyrhizobium sp. S23321                                                    hypothetical protein S23_54210 [Bradyrhizobium sp. S23321].                                                                    <-383773656_?||383773657_?->383773658_?-><-383773659_SRAP*||383773660_?->383773661_NUDIX->383773662_?->       365890954    NUDIX-><-SRAP*||?->NUDIX->                                                 BRAS3809_5540014                204   bacteria>proteobacteria>alphaproteobacteria    Bradyrhizobium sp. STM 3809                                                  conserved hypothetical protein [Bradyrhizobium sp. STM 3809].                                                                  365890951_?->365890952_?->365890953_NUDIX-><-365890954_SRAP*||365890955_?->365890956_NUDIX->365890957_?->       365897924    NUDIX-><-SRAP*||?->NUDIX->                                                 BRAO3843_2730057                204   bacteria>proteobacteria>alphaproteobacteria    Bradyrhizobium sp. STM 3843                                                  conserved hypothetical protein [Bradyrhizobium sp. STM 3843].                                                                  365897921_?->365897922_?->365897923_NUDIX-><-365897924_SRAP*||365897925_?->365897926_NUDIX->365897927_?->       386397340    NUDIX->?->?-><-SRAP*||?->NUDIX->                                           Bra1253DRAFT_02856              254   bacteria>proteobacteria>alphaproteobacteria    Bradyrhizobium sp. WSM1253                                                   hypothetical protein Bra1253DRAFT_02856 [Bradyrhizobium sp. WSM1253].                                                          386397337_NUDIX->386397338_?->386397339_?-><-386397340_SRAP*||386397341_?->386397342_NUDIX->386397343_?->       374573835    <-NUDIX<-?||SRAP*-><-?<-?<-NUDIX                                           Bra471DRAFT_02427               251   bacteria>proteobacteria>alphaproteobacteria    Bradyrhizobium sp. WSM471                                                    hypothetical protein Bra471DRAFT_02427 [Bradyrhizobium sp. WSM471].                                                            <-374573832_?<-374573833_NUDIX<-374573834_?||374573835_SRAP*-><-374573836_?<-374573837_?<-374573838_NUDIX       398822397    NUDIX->?->?-><-SRAP*||?->NUDIX->                                           PMI42_03486                     253   bacteria>proteobacteria>alphaproteobacteria    Bradyrhizobium sp. YR681                                                     hypothetical protein PMI42_03486 [Bradyrhizobium sp. YR681].                                                                   398822394_NUDIX->398822395_?->398822396_?-><-398822397_SRAP*||398822398_?->398822399_NUDIX->398822400_?->       479598099    <-NUDIX||SRAP*->                                                           C032_00610                      259   bacteria>proteobacteria>alphaproteobacteria    Brucella abortus 63/294                                                      hypothetical protein C032_00610 [Brucella abortus 63/294].                                                                     <-479598096_?<-479598097_?<-479598098_NUDIX||479598099_SRAP*->479598100_?-><-479598101_?||479598102_?->       261218958    <-SRAP*||NUDIX->                                                           BAJG_02403                      206   bacteria>proteobacteria>alphaproteobacteria    Brucella ceti M13/05/1                                                       conserved hypothetical protein [Brucella ceti M13/05/1].                                                                       261218955_?->261218956_?-><-261218957_?<-261218958_SRAP*||261218959_NUDIX->261218960_?->261218961_?->       225627171    <-NUDIX||SRAP*->                                                           BCETI_2000141                   259   bacteria>proteobacteria>alphaproteobacteria    Brucella ceti str. Cudo                                                      Hypothetical protein, conserved [Brucella ceti str. Cudo].                                                                     <-225627168_?<-225627169_?<-225627170_NUDIX||225627171_SRAP*->225627172_?-><-225627173_?||225627174_?->       306845274    <-NUDIX||SRAP*->                                                           BIBO1_1959                      259   bacteria>proteobacteria>alphaproteobacteria    Brucella inopinata BO1                                                       protein of unknown function DUF159 [Brucella inopinata BO1].                                                                   <-306845271_?<-306845272_?<-306845273_NUDIX||306845274_SRAP*->306845275_?-><-306845276_?<-306845277_?       17987558     <-SRAP*||NUDIX->                                                           BMEI1275                        259   bacteria>proteobacteria>alphaproteobacteria    Brucella melitensis bv. 1 str. 16M                                           hypothetical protein BMEI1275 [Brucella melitensis bv. 1 str. 16M].                                                            <-17987555_?||17987556_?-><-17987557_?<-17987558_SRAP*||17987559_NUDIX->17987560_?-><-17987561_?       384211056    <-NUDIX||SRAP*->?->cysteinyl-tRNA_synthetase->                             BM590_A0690                     339   bacteria>proteobacteria>alphaproteobacteria    Brucella melitensis M5-90                                                    hypothetical protein [Brucella melitensis M5-90].                                                                              <-384211053_?<-384211054_?<-384211055_NUDIX||384211056_SRAP*->384211057_?->384211058_cysteinyl-tRNA_synthetase->384211059_?->       265983795    <-NUDIX||SRAP*->                                                           BAKG_00124                      259   bacteria>proteobacteria>alphaproteobacteria    Brucella sp. 83/13                                                           conserved hypothetical protein [Brucella sp. 83/13].                                                                           <-265983792_?<-265983793_?<-265983794_NUDIX||265983795_SRAP*->265983796_?-><-265983797_?<-265983798_?       306842062    <-SRAP*||NUDIX->                                                           BIBO2_1846                      259   bacteria>proteobacteria>alphaproteobacteria    Brucella sp. BO2                                                             protein of unknown function DUF159 [Brucella sp. BO2].                                                                         <-306842059_?||306842060_?-><-306842061_?<-306842062_SRAP*||306842063_NUDIX->306842064_?->306842065_?->       294852036    <-NUDIX||SRAP*->                                                           BAZG_00952                      259   bacteria>proteobacteria>alphaproteobacteria    Brucella sp. NVSL 07-0026                                                    hypothetical protein BAZG_00952 [Brucella sp. NVSL 07-0026].                                                                   <-294852033_?<-294852034_?<-294852035_NUDIX||294852036_SRAP*->294852037_?-><-294852038_?||294852039_?->       163842944    <-NUDIX||SRAP*->                                                           BSUIS_A0701                     259   bacteria>proteobacteria>alphaproteobacteria    Brucella suis ATCC 23445                                                     hypothetical protein BSUIS_A0701 [Brucella suis ATCC 23445].                                                                   <-163842941_?<-163842942_?<-163842943_NUDIX||163842944_SRAP*->163842945_?-><-163842946_?<-163842947_?       110633318    <-NUDIX||SRAP*->cysteinyl-tRNA_synthetase->                                -                               246   bacteria>proteobacteria>alphaproteobacteria    Chelativorans sp. BNC1                                                       hypothetical protein Meso_0964 [Chelativorans sp. BNC1].                                                                       <-110633315_?<-110633316_?<-110633317_NUDIX||110633318_SRAP*->110633319_cysteinyl-tRNA_synthetase->110633320_?->110633321_?->       114704770    <-cysteinyl-tRNA_synthetase||?-><-SRAP*||NUDIX->                           -                               288   bacteria>proteobacteria>alphaproteobacteria    Fulvimarina pelagi HTCC2506                                                  hypothetical protein FP2506_07536 [Fulvimarina pelagi HTCC2506].                                                               114704767_?-><-114704768_cysteinyl-tRNA_synthetase||114704769_?-><-114704770_SRAP*||114704771_NUDIX->114704772_?->114704773_?->       163760570    <-SRAP*||NUDIX->                                                           -                               252   bacteria>proteobacteria>alphaproteobacteria    Hoeflea phototrophica DFL-43                                                 hypothetical protein HPDFL43_11766 [Hoeflea phototrophica DFL-43].                                                             <-163760567_?<-163760568_?<-163760569_?<-163760570_SRAP*||163760571_NUDIX-><-163760572_?||163760573_?->       118589250    <-NUDIX<-?||SRAP*->                                                        SIAM614_06893                   251   bacteria>proteobacteria>alphaproteobacteria    Labrenzia aggregata IAM 12614                                                hypothetical protein SIAM614_06893 [Labrenzia aggregata IAM 12614].                                                            <-118589247_?<-118589248_NUDIX<-118589249_?||118589250_SRAP*->118589251_?-><-118589252_?<-118589253_?       254504903    <-NUDIX<-?||SRAP*->                                                        SADFL11_4942                    248   bacteria>proteobacteria>alphaproteobacteria    Labrenzia alexandrii DFL-11                                                  conserved hypothetical protein [Labrenzia alexandrii DFL-11].                                                                  <-254503853_?<-254504556_NUDIX<-254504435_?||254504903_SRAP*-><-254501254_?<-254504253_?<-254501599_?       359790186    <-SRAP*<-?||NUDIX->                                                        MAXJ12_12292                    252   bacteria>proteobacteria>alphaproteobacteria    Mesorhizobium alhagi CCNWXJ12-2                                              hypothetical protein MAXJ12_12292 [Mesorhizobium alhagi CCNWXJ12-2].                                                           <-359790183_?<-359790184_?||359790185_?-><-359790186_SRAP*<-359790187_?||359790188_NUDIX->359790189_?->       357025804    <-NUDIX||SRAP*-><-?||cysteinyl-tRNA_synthetase->                           MEA186_13692                    258   bacteria>proteobacteria>alphaproteobacteria    Mesorhizobium amorphae CCNWGS0123                                            hypothetical protein MEA186_13692 [Mesorhizobium amorphae CCNWGS0123].                                                         <-357025801_?<-357025802_?<-357025803_NUDIX||357025804_SRAP*-><-357025805_?||357025806_cysteinyl-tRNA_synthetase->357025807_?->       433776086    <-SRAP*||NUDIX->                                                           -                               253   bacteria>proteobacteria>alphaproteobacteria    Mesorhizobium australicum WSM2073                                            hypothetical protein Mesau_04856 [Mesorhizobium australicum WSM2073].                                                          <-433776083_?||433776084_?->433776085_?-><-433776086_SRAP*||433776087_NUDIX->433776088_?->433776089_?->       319784482    <-SRAP*||NUDIX->                                                           -                               253   bacteria>proteobacteria>alphaproteobacteria    Mesorhizobium ciceri biovar biserrulae WSM1271                               hypothetical protein [Mesorhizobium ciceri biovar biserrulae WSM1271].                                                         <-319784479_?<-319784480_?||319784481_?-><-319784482_SRAP*||319784483_NUDIX->319784484_?->319784485_?->       13476468     <-NUDIX||SRAP*-><-?||cysteinyl-tRNA_synthetase->                           -                               369   bacteria>proteobacteria>alphaproteobacteria    Mesorhizobium loti MAFF303099                                                hypothetical protein mlr7795 [Mesorhizobium loti MAFF303099].                                                                  <-13476465_?<-13476466_?<-13476467_NUDIX||13476468_SRAP*-><-13476469_?||13476470_cysteinyl-tRNA_synthetase->13476471_?->       472451931    <-NUDIX||SRAP*->                                                           MESS2_200003                    253   bacteria>proteobacteria>alphaproteobacteria    Mesorhizobium metallidurans STM 2683                                         conserved hypothetical protein [Mesorhizobium metallidurans STM 2683].                                                         <-472451929_?<-472451930_NUDIX||472451931_SRAP*-><-472451932_?||472451933_?->472451934_?->       337269751    <-SRAP*||NUDIX->                                                           -                               253   bacteria>proteobacteria>alphaproteobacteria    Mesorhizobium opportunistum WSM2075                                          hypothetical protein Mesop_5296 [Mesorhizobium opportunistum WSM2075].                                                         <-337269748_?||337269749_?->337269750_?-><-337269751_SRAP*||337269752_NUDIX->337269753_?->337269754_?->       474661774    <-NUDIX||SRAP*->                                                           MESS4_470006                    253   bacteria>proteobacteria>alphaproteobacteria    Mesorhizobium sp. STM 4661                                                   conserved hypothetical protein [Mesorhizobium sp. STM 4661].                                                                   <-474661771_?<-474661772_?<-474661773_NUDIX||474661774_SRAP*->474661775_?->474661776_?->474661777_?->       390449896    <-NUDIX||?->SRAP*->                                                        A33O_10329                      193   bacteria>proteobacteria>alphaproteobacteria    Nitratireductor aquibiodomus RA22                                            hypothetical protein A33O_10329 [Nitratireductor aquibiodomus RA22].                                                           <-390449893_?<-390449894_NUDIX||390449895_?->390449896_SRAP*-><-390449897_?<-390449898_?||390449899_?->       407974574    <-SRAP*||NUDIX->                                                           NA8A_09729                      252   bacteria>proteobacteria>alphaproteobacteria    Nitratireductor indicus C115                                                 hypothetical protein NA8A_09729 [Nitratireductor indicus C115].                                                                407974571_?->407974572_?->407974573_?-><-407974574_SRAP*||407974575_NUDIX->407974576_?->407974577_?->       407777086    <-SRAP*||NUDIX->                                                           NA2_03922                       251   bacteria>proteobacteria>alphaproteobacteria    Nitratireductor pacificus pht-3B                                             hypothetical protein NA2_03922 [Nitratireductor pacificus pht-3B].                                                             <-407777083_?<-407777084_?||407777085_?-><-407777086_SRAP*||407777087_NUDIX->407777088_?->407777089_?->       85714357     <-SRAP*||?->NUDIX->                                                        NB311A_15437                    255   bacteria>proteobacteria>alphaproteobacteria    Nitrobacter sp. Nb-311A                                                      hypothetical protein NB311A_15437 [Nitrobacter sp. Nb-311A].                                                                   85714354_?->85714355_?->85714356_?-><-85714357_SRAP*||85714358_?->85714359_NUDIX->85714360_?->       75676882     Sua5->FAD_linked_oxidase-><-SRAP*||?->NUDIX->                              -                               255   bacteria>proteobacteria>alphaproteobacteria    Nitrobacter winogradskyi Nb-255                                              hypothetical protein Nwi_2698 [Nitrobacter winogradskyi Nb-255].                                                               75676879_?->75676880_Sua5->75676881_FAD_linked_oxidase-><-75676882_SRAP*||75676883_?->75676884_NUDIX->75676885_?->       83945382     <-NUDIX||SRAP*->                                                           -                               219   bacteria>proteobacteria>alphaproteobacteria    Oceanicaulis sp. HTCC2633                                                    hypothetical protein OA2633_14386 [Oceanicaulis sp. HTCC2633].                                                                 <-83945379_?<-83945380_?<-83945381_NUDIX||83945382_SRAP*-><-83945383_?<-83945384_?<-83945385_?       153009940    <-SRAP*||NUDIX->                                                           -                               262   bacteria>proteobacteria>alphaproteobacteria    Ochrobactrum anthropi ATCC 49188                                             hypothetical protein Oant_2613 [Ochrobactrum anthropi ATCC 49188].                                                             <-153009937_?<-153009938_?||153009939_?-><-153009940_SRAP*||153009941_NUDIX->153009942_?->153009943_?->       404320770    <-NUDIX||SRAP*->                                                           OantC_010100021352              259   bacteria>proteobacteria>alphaproteobacteria    Ochrobactrum anthropi CTS-325                                                hypothetical protein OantC_21352 [Ochrobactrum anthropi CTS-325].                                                              <-404320767_?<-404320768_?<-404320769_NUDIX||404320770_SRAP*-><-404320771_?||404320772_?->404320773_?->       239831510    <-NUDIX||SRAP*->                                                           -                               302   bacteria>proteobacteria>alphaproteobacteria    Ochrobactrum intermedium LMG 3301                                            Hypothetical protein, conserved [Ochrobactrum intermedium LMG 3301].                                                           <-239831507_?<-239831508_?<-239831509_NUDIX||239831510_SRAP*-><-239831511_?||239831512_?->239831513_?->       444311665    <-NUDIX||SRAP*->                                                           D584_17890                      259   bacteria>proteobacteria>alphaproteobacteria    Ochrobactrum intermedium M86                                                 hypothetical protein D584_17890 [Ochrobactrum intermedium M86].                                                                <-444311662_?<-444311663_?<-444311664_NUDIX||444311665_SRAP*-><-444311666_?||444311667_?->444311668_?->       459782825    <-NUDIX||SRAP*->                                                           WYI_04891                       39    bacteria>proteobacteria>alphaproteobacteria    Ochrobactrum sp. CDB2                                                        hypothetical protein WYI_04891, partial [Ochrobactrum sp. CDB2].                                                               <-459782822_?<-459782823_?<-459782824_NUDIX||459782825_SRAP*->       209886042    Sua5->FAD_linked_oxidase-><-SRAP*||?->NUDIX->                              -                               251   bacteria>proteobacteria>alphaproteobacteria    Oligotropha carboxidovorans OM5                                              hypothetical protein OCAR_6926 [Oligotropha carboxidovorans OM5].                                                              209886039_?->209886040_Sua5->209886041_FAD_linked_oxidase-><-209886042_SRAP*||209886043_?->209886044_NUDIX->209886045_?->       357383644    <-NUDIX||SRAP*->                                                           -                               235   bacteria>proteobacteria>alphaproteobacteria    Pelagibacterium halotolerans B2                                              hypothetical protein [Pelagibacterium halotolerans B2].                                                                        <-357383641_?<-357383642_?<-357383643_NUDIX||357383644_SRAP*->357383645_?-><-357383646_?||357383647_?->       398831495    <-SRAP*||NUDIX->                                                           PMI41_04573                     254   bacteria>proteobacteria>alphaproteobacteria    Phyllobacterium sp. YR531                                                    hypothetical protein PMI41_04573 [Phyllobacterium sp. YR531].                                                                  <-398831492_?||398831493_?-><-398831494_?<-398831495_SRAP*||398831496_NUDIX->398831497_?->398831498_?->       328544937    FAD_linked_oxidase-><-?<-SRAP*||?->NUDIX->                                 -                               248   bacteria>proteobacteria>alphaproteobacteria    Polymorphum gilvum SL003B-26A1                                               hypothetical protein SL003B_3320 [Polymorphum gilvum SL003B-26A1].                                                             <-328544934_?||328544935_FAD_linked_oxidase-><-328544936_?<-328544937_SRAP*||328544938_?->328544939_NUDIX->328544940_?->       374329990    <-NUDIX<-?||SRAP*->?-><-FAD_linked_oxidase                                 -                               185   bacteria>proteobacteria>alphaproteobacteria    Pseudovibrio sp. FO-BEG1                                                     hypothetical protein PSE_1640 [Pseudovibrio sp. FO-BEG1].                                                                      <-374329987_?<-374329988_NUDIX<-374329989_?||374329990_SRAP*->374329991_?-><-374329992_FAD_linked_oxidase||374329993_?->       254471804    FAD_linked_oxidase-><-?<-SRAP*||?->NUDIX->                                 PJE062_189                      255   bacteria>proteobacteria>alphaproteobacteria    Pseudovibrio sp. JE062                                                       conserved hypothetical protein [Pseudovibrio sp. JE062].                                                                       254471936_?->254472079_FAD_linked_oxidase-><-254471702_?<-254471804_SRAP*||254471669_?->254471688_NUDIX->254471679_?->       218515051    <-NUDIX||?->SRAP*->                                                        Retl8_010100015851              73    bacteria>proteobacteria>alphaproteobacteria    Rhizobium etli 8C-3                                                          hypothetical protein Retl8_15851, partial [Rhizobium etli 8C-3].                                                               <-218515049_NUDIX||218515050_?->218515051_SRAP*->       86357047     <-NUDIX||?->SRAP*->                                                        -                               273   bacteria>proteobacteria>alphaproteobacteria    Rhizobium etli CFN 42                                                        hypothetical protein RHE_CH01409 [Rhizobium etli CFN 42].                                                                      <-86357044_?<-86357045_NUDIX||86357046_?->86357047_SRAP*-><-86357048_?<-86357049_?||86357050_?->       190891093    <-NUDIX||?->SRAP*->                                                        -                               254   bacteria>proteobacteria>alphaproteobacteria    Rhizobium etli CIAT 652                                                      hypothetical protein RHECIAT_CH0001478 [Rhizobium etli CIAT 652].                                                              <-190891090_?<-190891091_NUDIX||190891092_?->190891093_SRAP*-><-190891094_?<-190891095_?<-190891096_?       417103439    <-SRAP*||NUDIX->                                                           RHECNPAF_330017                 254   bacteria>proteobacteria>alphaproteobacteria    Rhizobium etli CNPAF512                                                      hypothetical protein RHECNPAF_330017 [Rhizobium etli CNPAF512].                                                                417103436_?->417103437_?->417103438_?-><-417103439_SRAP*||417103440_NUDIX->417103441_?->417103442_?->       478274360    <-NUDIX||?->SRAP*->                                                        RHSP_35341                      241   bacteria>proteobacteria>alphaproteobacteria    Rhizobium freirei PRF 81                                                     protein of unknown function DUF159 [Rhizobium freirei PRF 81].                                                                 <-478274357_?<-478274358_NUDIX||478274359_?->478274360_SRAP*-><-478274361_?<-478274362_?<-478274363_?       241203909    <-NUDIX||?->SRAP*->                                                        -                               254   bacteria>proteobacteria>alphaproteobacteria    Rhizobium leguminosarum bv. trifolii WSM1325                                 hypothetical protein Rleg_1171 [Rhizobium leguminosarum bv. trifolii WSM1325].                                                 <-241203906_?<-241203907_NUDIX||241203908_?->241203909_SRAP*-><-241203910_?<-241203911_?<-241203912_?       424891028    <-SRAP*<-?||NUDIX->                                                        Rleg10DRAFT_1745                254   bacteria>proteobacteria>alphaproteobacteria    Rhizobium leguminosarum bv. trifolii WSM2012                                 hypothetical protein Rleg10DRAFT_1745 [Rhizobium leguminosarum bv. trifolii WSM2012].                                          424891025_?->424891026_?->424891027_?-><-424891028_SRAP*<-424891029_?||424891030_NUDIX->424891031_?->       424894378    <-NUDIX||SRAP*->                                                           Rleg4DRAFT_0212                 254   bacteria>proteobacteria>alphaproteobacteria    Rhizobium leguminosarum bv. trifolii WSM2297                                 hypothetical protein Rleg4DRAFT_0212 [Rhizobium leguminosarum bv. trifolii WSM2297].                                           <-424894375_?<-424894376_?<-424894377_NUDIX||424894378_SRAP*-><-424894379_?<-424894380_?<-424894381_?       209548622    <-NUDIX||?->SRAP*->                                                        -                               254   bacteria>proteobacteria>alphaproteobacteria    Rhizobium leguminosarum bv. trifolii WSM2304                                 hypothetical protein Rleg2_1019 [Rhizobium leguminosarum bv. trifolii WSM2304].                                                <-209548619_?<-209548620_NUDIX||209548621_?->209548622_SRAP*-><-209548623_?<-209548624_?<-209548625_?       424914769    <-SRAP*<-?||NUDIX->                                                        Rleg9DRAFT_2300                 254   bacteria>proteobacteria>alphaproteobacteria    Rhizobium leguminosarum bv. trifolii WSM597                                  hypothetical protein Rleg9DRAFT_2300 [Rhizobium leguminosarum bv. trifolii WSM597].                                            424914766_?->424914767_?->424914768_?-><-424914769_SRAP*<-424914770_?||424914771_NUDIX->424914772_?->       424880873    <-NUDIX||?->SRAP*->                                                        Rleg8DRAFT_2422                 254   bacteria>proteobacteria>alphaproteobacteria    Rhizobium leguminosarum bv. trifolii WU95                                    hypothetical protein Rleg8DRAFT_2422 [Rhizobium leguminosarum bv. trifolii WU95].                                              <-424880870_?<-424880871_NUDIX||424880872_?->424880873_SRAP*-><-424880874_?<-424880875_?<-424880876_?       116251297    <-NUDIX||?->SRAP*->                                                        -                               254   bacteria>proteobacteria>alphaproteobacteria    Rhizobium leguminosarum bv. viciae 3841                                      hypothetical protein RL1531 [Rhizobium leguminosarum bv. viciae 3841].                                                         <-116251294_?<-116251295_NUDIX||116251296_?->116251297_SRAP*-><-116251298_?<-116251299_?<-116251300_?       424909942    <-NUDIX||?->SRAP*->                                                        Rleg13DRAFT_02134               253   bacteria>proteobacteria>alphaproteobacteria    Rhizobium leguminosarum bv. viciae USDA 2370                                 hypothetical protein Rleg13DRAFT_02134 [Rhizobium leguminosarum bv. viciae USDA 2370].                                         <-424909939_?<-424909940_NUDIX||424909941_?->424909942_SRAP*-><-424909943_?<-424909944_?<-424909945_?       424874588    <-NUDIX||?->SRAP*->                                                        Rleg5DRAFT_6144                 254   bacteria>proteobacteria>alphaproteobacteria    Rhizobium leguminosarum bv. viciae WSM1455                                   hypothetical protein Rleg5DRAFT_6144 [Rhizobium leguminosarum bv. viciae WSM1455].                                             <-424874585_?<-424874586_NUDIX||424874587_?->424874588_SRAP*-><-424874589_?<-424874590_?<-424874591_?       409436643    <-NUDIX||?->SRAP*->                                                        -                               253   bacteria>proteobacteria>alphaproteobacteria    Rhizobium mesoamericanum STM3625                                             conserved hypothetical protein [Rhizobium mesoamericanum STM3625].                                                             <-409436640_?<-409436641_NUDIX||409436642_?->409436643_SRAP*->409436644_?-><-409436645_?<-409436646_?       430003031    <-NUDIX||SRAP*->                                                           NT26_1090                       251   bacteria>proteobacteria>alphaproteobacteria    Rhizobium sp.                                                                conserved protein of unknown function [Rhizobium sp.].                                                                         <-430003028_?<-430003029_?<-430003030_NUDIX||430003031_SRAP*->430003032_?-><-430003033_?<-430003034_?       398378498    <-SRAP*<-?||NUDIX->                                                        PMI03_02274                     248   bacteria>proteobacteria>alphaproteobacteria    Rhizobium sp. AP16                                                           hypothetical protein PMI03_02274 [Rhizobium sp. AP16].                                                                         398378495_?->398378496_?->398378497_?-><-398378498_SRAP*<-398378499_?||398378500_NUDIX->398378501_?->       402486341    <-SRAP*<-?||NUDIX->                                                        RCCGE510_01520                  254   bacteria>proteobacteria>alphaproteobacteria    Rhizobium sp. CCGE 510                                                       hypothetical protein RCCGE510_01520 [Rhizobium sp. CCGE 510].                                                                  402486338_?->402486339_?->402486340_?-><-402486341_SRAP*<-402486342_?||402486343_NUDIX->402486344_?->       399038547    <-SRAP*<-?||NUDIX->                                                        PMI09_02127                     254   bacteria>proteobacteria>alphaproteobacteria    Rhizobium sp. CF122                                                          hypothetical protein PMI09_02127 [Rhizobium sp. CF122].                                                                        399038544_?->399038545_?-><-399038546_?<-399038547_SRAP*<-399038548_?||399038549_NUDIX->       405380058    <-NUDIX||?->SRAP*->                                                        PMI11_03885                     254   bacteria>proteobacteria>alphaproteobacteria    Rhizobium sp. CF142                                                          hypothetical protein PMI11_03885 [Rhizobium sp. CF142].                                                                        <-405380055_?<-405380056_NUDIX||405380057_?->405380058_SRAP*-><-405380059_?<-405380060_?<-405380061_?       418936997    <-NUDIX||SRAP*->                                                           PDO_1468                        256   bacteria>proteobacteria>alphaproteobacteria    Rhizobium sp. PDO1-076                                                       protein of unknown function DUF159 [Rhizobium sp. PDO1-076].                                                                   <-418936994_?<-418936995_?<-418936996_NUDIX||418936997_SRAP*-><-418936998_?<-418936999_?<-418937000_?       421587278    <-SRAP*<-?||NUDIX->                                                        RCCGEPOP_01714                  254   bacteria>proteobacteria>alphaproteobacteria    Rhizobium sp. Pop5                                                           hypothetical protein RCCGEPOP_01714 [Rhizobium sp. Pop5].                                                                      421587275_?->421587276_?-><-421587277_?<-421587278_SRAP*<-421587279_?||421587280_NUDIX->421587281_?->       440226046    <-NUDIX||?->SRAP*->                                                        -                               254   bacteria>proteobacteria>alphaproteobacteria    Rhizobium tropici CIAT 899                                                   hypothetical protein RTCIAT899_CH05920 [Rhizobium tropici CIAT 899].                                                           <-440226043_?<-440226044_NUDIX||440226045_?->440226046_SRAP*-><-440226047_?<-440226048_?<-440226049_?       115526376    <-SRAP*||?->NUDIX->                                                        -                               258   bacteria>proteobacteria>alphaproteobacteria    Rhodopseudomonas palustris BisA53                                            hypothetical protein RPE_4383 [Rhodopseudomonas palustris BisA53].                                                             115526373_?->115526374_?-><-115526375_?<-115526376_SRAP*||115526377_?->115526378_NUDIX->115526379_?->       90425797     <-SRAP*||?->NUDIX->                                                        -                               257   bacteria>proteobacteria>alphaproteobacteria    Rhodopseudomonas palustris BisB18                                            hypothetical protein RPC_4325 [Rhodopseudomonas palustris BisB18].                                                             90425794_?->90425795_?-><-90425796_?<-90425797_SRAP*||90425798_?->90425799_NUDIX->90425800_?->       91975725     <-NUDIX<-?||SRAP*->                                                        -                               259   bacteria>proteobacteria>alphaproteobacteria    Rhodopseudomonas palustris BisB5                                             hypothetical protein RPD_1245 [Rhodopseudomonas palustris BisB5].                                                              <-91975722_?<-91975723_NUDIX<-91975724_?||91975725_SRAP*-><-91975726_?<-91975727_?<-91975728_?       39934150     <-NUDIX<-?||SRAP*->                                                        -                               257   bacteria>proteobacteria>alphaproteobacteria    Rhodopseudomonas palustris CGA009                                            hypothetical protein RPA1075 [Rhodopseudomonas palustris CGA009].                                                              <-39934147_?<-39934148_NUDIX<-39934149_?||39934150_SRAP*-><-39934151_?<-39934152_?||39934153_?->       316932618    <-NUDIX<-?||SRAP*->                                                        -                               257   bacteria>proteobacteria>alphaproteobacteria    Rhodopseudomonas palustris DX-1                                              hypothetical protein [Rhodopseudomonas palustris DX-1].                                                                        <-316932615_?<-316932616_NUDIX<-316932617_?||316932618_SRAP*-><-316932619_?<-316932620_?||316932621_?->       86748255     <-NUDIX<-?||SRAP*-><-FAD_linked_oxidase<-Sua5                              -                               259   bacteria>proteobacteria>alphaproteobacteria    Rhodopseudomonas palustris HaA2                                              hypothetical protein RPB_1130 [Rhodopseudomonas palustris HaA2].                                                               <-86748252_?<-86748253_NUDIX<-86748254_?||86748255_SRAP*-><-86748256_FAD_linked_oxidase<-86748257_Sua5<-86748258_?       192289673    <-NUDIX<-?||SRAP*->                                                        -                               257   bacteria>proteobacteria>alphaproteobacteria    Rhodopseudomonas palustris TIE-1                                             hypothetical protein Rpal_1263 [Rhodopseudomonas palustris TIE-1].                                                             <-192289670_?<-192289671_NUDIX<-192289672_?||192289673_SRAP*->192289674_?->192289675_?-><-192289676_?       402849084    <-SRAP*||?->NUDIX->                                                        A33M_2193                       259   bacteria>proteobacteria>alphaproteobacteria    Rhodovulum sp. PH10                                                          Gifsy-2 prophage protein [Rhodovulum sp. PH10].                                                                                402849081_?-><-402849082_?<-402849083_?<-402849084_SRAP*||402849085_?->402849086_NUDIX->402849087_?->       307941563    <-SRAP*||?->NUDIX->                                                        TRICHSKD4_0160                  247   bacteria>proteobacteria>alphaproteobacteria    Roseibium sp. TrichSKD4                                                      protein YoqW [Roseibium sp. TrichSKD4].                                                                                        307941560_?->307941561_?-><-307941562_?<-307941563_SRAP*||307941564_?->307941565_NUDIX->307941566_?->       378825383    <-NUDIX||SRAP*->                                                           -                               271   bacteria>proteobacteria>alphaproteobacteria    Sinorhizobium fredii HH103                                                   hypothetical protein SFHH103_00791 [Sinorhizobium fredii HH103].                                                               <-378825380_?<-378825381_?<-378825382_NUDIX||378825383_SRAP*-><-378825384_?||378825385_?->378825386_?->       227821435    <-NUDIX||SRAP*->                                                           -                               257   bacteria>proteobacteria>alphaproteobacteria    Sinorhizobium fredii NGR234                                                  hypothetical protein NGR_c08610 [Sinorhizobium fredii NGR234].                                                                 <-227821432_?<-227821433_?<-227821434_NUDIX||227821435_SRAP*->227821436_?->227821437_?->227821438_?->       398350717    <-NUDIX||SRAP*->                                                           USDA257_c08320                  270   bacteria>proteobacteria>alphaproteobacteria    Sinorhizobium fredii USDA 257                                                hypothetical protein USDA257_c08320 [Sinorhizobium fredii USDA 257].                                                           <-398350714_?<-398350715_?<-398350716_NUDIX||398350717_SRAP*-><-398350718_?<-398350719_?||398350720_?->       150395935    <-NUDIX||SRAP*->                                                           -                               256   bacteria>proteobacteria>alphaproteobacteria    Sinorhizobium medicae WSM419                                                 hypothetical protein Smed_0711 [Sinorhizobium medicae WSM419].                                                                 <-150395932_?<-150395933_?<-150395934_NUDIX||150395935_SRAP*-><-150395936_?||150395937_?->150395938_?->       15964862     <-NUDIX||SRAP*-><-?||cysteinyl-tRNA_synthetase->                           -                               276   bacteria>proteobacteria>alphaproteobacteria    Sinorhizobium meliloti 1021                                                  hypothetical protein SMc02553 [Sinorhizobium meliloti 1021].                                                                   <-15964859_?<-15964860_?<-15964861_NUDIX||15964862_SRAP*-><-15964863_?||15964864_cysteinyl-tRNA_synthetase->15964865_?->       470188062    <-NUDIX||SRAP*->                                                           -                               256   bacteria>proteobacteria>alphaproteobacteria    Sinorhizobium meliloti 2011                                                  hypothetical protein SM2011_c02553 [Sinorhizobium meliloti 2011].                                                              <-470188059_?<-470188060_?<-470188061_NUDIX||470188062_SRAP*-><-470188063_?||470188064_?->470188065_?->       71906715     <-NUDIX<-?<-SRAP*                                                          -                               214   bacteria>proteobacteria>betaproteobacteria     Dechloromonas aromatica RCB                                                  hypothetical protein Daro_1076 [Dechloromonas aromatica RCB].                                                                  <-71906712_?<-71906713_NUDIX<-71906714_?<-71906715_SRAP*<-71906716_?<-71906717_?<-71906718_?       423140407    <-NUDIX<-?||?->SRAP*->                                                     -                               227   bacteria>proteobacteria>gammaproteobacteria    Salmonella enterica subsp. houtenae str. ATCC BAA-1581                       hypothetical protein SEHO0A_01924 [Salmonella enterica subsp. houtenae str. ATCC BAA-1581].                                    <-423140404_NUDIX<-423140405_?||423140406_?->423140407_SRAP*-><-423140408_?||423140409_?-><-423140410_?       425898636    <-SRAP*<-?<-?||NUDIX->                                                     -                               129   bacteria>proteobacteria>gammaproteobacteria    Pseudomonas chlororaphis subsp. aureofaciens 30-84                           hypothetical protein Pchl3084_2015 [Pseudomonas chlororaphis subsp. aureofaciens 30-84].                                       <-425898633_?||425898634_?-><-425898635_?<-425898636_SRAP*<-425898637_?<-425898638_?||425898639_NUDIX->        # UDG-associated        329848884    <-SRAP*<-?<-Uracil_DNA_glycosylase                                         ABI_19560                       205   bacteria>proteobacteria>alphaproteobacteria    Asticcacaulis biprosthecum C19                                               hypothetical protein ABI_19560 [Asticcacaulis biprosthecum C19].                                                               <-329848881_?||329848882_?->329848883_?-><-329848884_SRAP*<-329848885_?<-329848886_Uracil_DNA_glycosylase||329848887_?->       315500450    <-Uracil_DNA_glycosylase<-SRAP*                                            Astex_3470                      159   bacteria>proteobacteria>alphaproteobacteria    Asticcacaulis excentricus CB 48                                              hypothetical protein Astex_3470 [Asticcacaulis excentricus CB 48].                                                             <-315500447_?||315500448_?-><-315500449_Uracil_DNA_glycosylase<-315500450_SRAP*||315500451_?-><-315500452_?||315500453_?->       359398350    SRAP*-><-?<-?||Uracil_DNA_glycosylase->                                    NSU_1057                        206   bacteria>proteobacteria>alphaproteobacteria    Novosphingobium pentaromativorans US6-1                                      hypothetical protein NSU_1057 [Novosphingobium pentaromativorans US6-1].                                                       <-359398347_?||359398348_?-><-359398349_?||359398350_SRAP*-><-359398351_?<-359398352_?||359398353_Uracil_DNA_glycosylase->       399064648    SRAP*->Uracil_DNA_glycosylase->                                            PMI02_03964                     208   bacteria>proteobacteria>alphaproteobacteria    Novosphingobium sp. AP12                                                     hypothetical protein PMI02_03964 [Novosphingobium sp. AP12].                                                                   <-399064645_?<-399064646_?<-399064647_?||399064648_SRAP*->399064649_Uracil_DNA_glycosylase-><-399064650_?<-399064651_?       393773817    <-Uracil_DNA_glycosylase<-SRAP*                                            WSK_3206                        210   bacteria>proteobacteria>alphaproteobacteria    Novosphingobium sp. Rr 2-17                                                  hypothetical protein WSK_3206 [Novosphingobium sp. Rr 2-17].                                                                   393773814_?-><-393773815_?<-393773816_Uracil_DNA_glycosylase<-393773817_SRAP*||393773818_?->393773819_?->393773820_?->       402823548    SRAP*->?->Uracil_DNA_glycosylase->                                         LH128_11688                     208   bacteria>proteobacteria>alphaproteobacteria    Sphingomonas sp. LH128                                                       hypothetical protein LH128_11688 [Sphingomonas sp. LH128].                                                                     <-402823547_?||402823548_SRAP*->402823549_?->402823550_Uracil_DNA_glycosylase-><-402823551_?       374853348    UDG->?-><-?<-SRAP*                                                         HGMM_F35G12C30                  226   bacteria                                       uncultured candidate division OP1 bacterium                                  hypothetical conserved protein [uncultured candidate division OP1 bacterium].                                                  374853345_UDG->374853346_?-><-374853347_?<-374853348_SRAP*<-374853349_?<-374853350_?<-374853351_?        # DNA polB-associated       317120976    SRAP*-><-?||?->DNA_pol_B_epsi->                                            -                               232   bacteria>firmicutes                            Thermaerobacter marianensis DSM 12885                                        hypothetical protein [Thermaerobacter marianensis DSM 12885].                                                                  <-317120973_?||317120974_?->317120975_?->317120976_SRAP*-><-317120977_?||317120978_?->317120979_DNA_pol_B_epsi->       410582362    <-DNA_pol_B_epsi<-?||?-><-SRAP*                                            -                               239   bacteria>firmicutes                            Thermaerobacter subterraneus DSM 13965                                       hypothetical protein ThesuDRAFT_00379 [Thermaerobacter subterraneus DSM 13965].                                                <-410582359_DNA_pol_B_epsi<-410582360_?||410582361_?-><-410582362_SRAP*<-410582363_?<-410582364_?||410582365_?->       398334668    DNA_pol_B->SRAP*->                                                         LkmesMB_010100002865            232   bacteria>spirochaetes                          Leptospira kmetyi serovar Malaysia str. Bejo-Iso9                            hypothetical protein LkmesMB_02865 [Leptospira kmetyi serovar Malaysia str. Bejo-Iso9].                                        398334665_?->398334666_?->398334667_DNA_pol_B->398334668_SRAP*-><-398334669_?<-398334670_?<-398334671_?       408794675    DNA_pol_B->SRAP*->                                                         -                               225   bacteria>spirochaetes                          Leptospira meyeri serovar Hardjo str. Went 5                                 hypothetical protein LEP1GSC017_3678 [Leptospira meyeri serovar Hardjo str. Went 5].                                           408794245_?->408794465_?->408794401_DNA_pol_B->408794675_SRAP*-><-408794380_?<-408794476_?<-408794341_?       398345538    <-SRAP*||?-><-DNA_pol_B                                                    LinasL1_010100021407            238   bacteria>spirochaetes                          Leptospira inadai serovar Lyme str. 10                                       hypothetical protein LinasL1_21407 [Leptospira inadai serovar Lyme str. 10].                                                   398345535_?->398345536_?-><-398345537_?<-398345538_SRAP*||398345539_?-><-398345540_DNA_pol_B<-398345541_?       359686739    <-SRAP*<-DNA_pol_B                                                         LlicsVM_010100000105            235   bacteria>spirochaetes                          Leptospira licerasiae serovar Varillal str. MMD0835                          hypothetical protein LlicsVM_00105 [Leptospira licerasiae serovar Varillal str. MMD0835].                                      359686736_?->359686737_?->359686738_?-><-359686739_SRAP*<-359686740_DNA_pol_B<-359686741_?<-359686742_?        # OGG in neighborhood       169824588    <-OGG-glycosylase<-?<-?<-SRAP*                                             FMG_0891                        194   bacteria>firmicutes                            Finegoldia magna ATCC 29328                                                  hypothetical protein FMG_0891 [Finegoldia magna ATCC 29328].                                                                   <-169824585_OGG-glycosylase<-169824586_?<-169824587_?<-169824588_SRAP*<-169824589_?<-169824590_?<-169824591_?       297588572    <-OGG-glycosylase<-?<-?<-SRAP*                                             -                               197   bacteria>firmicutes                            Finegoldia magna ATCC 53516                                                  conserved hypothetical protein [Finegoldia magna ATCC 53516].                                                                  <-297588569_OGG-glycosylase<-297588570_?<-297588571_?<-297588572_SRAP*<-297588573_?<-297588574_?<-297588575_?       302380363    <-OGG-glycosylase<-?<-?<-SRAP*                                             HMPREF9261_0810                 194   bacteria>firmicutes                            Finegoldia magna ACS-171-V-Col3                                              conserved hypothetical protein [Finegoldia magna ACS-171-V-Col3].                                                              <-302380379_OGG-glycosylase<-302380371_?<-302380356_?<-302380363_SRAP*<-302380342_?<-302380328_?<-302380335_?       303233639    <-OGG-glycosylase<-?<-?<-SRAP*                                             HMPREF9289_1398                 194   bacteria>firmicutes                            Finegoldia magna BVS033A4                                                    conserved hypothetical protein [Finegoldia magna BVS033A4].                                                                    <-303233727_OGG-glycosylase<-303233636_?<-303233759_?<-303233639_SRAP*<-303233734_?<-303233712_?<-303233648_?       417926108    SRAP*->?->?->OGG-glycosylase->                                             HMPREF9489_0217                 154   bacteria>firmicutes                            Finegoldia magna SY403409CC001050417                                         hypothetical protein HMPREF9489_0217 [Finegoldia magna SY403409CC001050417].                                                   417926109_?->417926100_?->417926119_?->417926108_SRAP*->417926077_?->417926114_?->417926059_OGG-glycosylase->        # SNase associated       408377844    SNase->?-><-SRAP*                                                          QWE_09645                       236   bacteria>proteobacteria>alphaproteobacteria    Agrobacterium albertimagni AOL15                                             hypothetical protein QWE_09645 [Agrobacterium albertimagni AOL15].                                                             <-408377841_?||408377842_SNase->408377843_?-><-408377844_SRAP*||408377845_?->408377846_?->408377847_?->       408377085    SRAP*->SNase->                                                             QWE_05808                       241   bacteria>proteobacteria>alphaproteobacteria    Agrobacterium albertimagni AOL15                                             hypothetical protein QWE_05808 [Agrobacterium albertimagni AOL15].                                                             <-408377082_?||408377083_?->408377084_?->408377085_SRAP*->408377086_SNase->408377087_?->408377088_?->       460063595    SNase-><-SRAP*                                                             -                               210   bacteria>proteobacteria>alphaproteobacteria    alpha proteobacterium HIMB114                                                hypothetical protein HIMB114_00013240 [alpha proteobacterium HIMB114].                                                         <-460063592_?||460063593_?->460063594_SNase-><-460063595_SRAP*||460063596_?->460063597_?-><-460063598_?       392382000    SRAP*->SNase->                                                             AZOBR_100150                    232   bacteria>proteobacteria>alphaproteobacteria    Azospirillum brasilense Sp245                                                protein of unknown function [Azospirillum brasilense Sp245].                                                                   392381997_?-><-392381998_?<-392381999_?||392382000_SRAP*->392382001_SNase->392382002_?-><-392382003_?       374291828    SRAP*->SNase->                                                             -                               246   bacteria>proteobacteria>alphaproteobacteria    Azospirillum lipoferum 4B                                                    hypothetical protein AZOLI_1317 [Azospirillum lipoferum 4B].                                                                   374291825_?-><-374291826_?<-374291827_?||374291828_SRAP*->374291829_SNase->374291830_?->374291831_?->       288958372    <-SNase<-SRAP*                                                             AZL_015310                      248   bacteria>proteobacteria>alphaproteobacteria    Azospirillum sp. B510                                                        hypothetical protein AZL_015310 [Azospirillum sp. B510].                                                                       288958369_?-><-288958370_?<-288958371_SNase<-288958372_SRAP*||288958373_?->288958374_?-><-288958375_?       110633601    <-SRAP*||?-><-SNase                                                        -                               226   bacteria>proteobacteria>alphaproteobacteria    Chelativorans sp. BNC1                                                       hypothetical protein Meso_1248 [Chelativorans sp. BNC1].                                                                       <-110633598_?<-110633599_?<-110633600_?<-110633601_SRAP*||110633602_?-><-110633603_SNase||110633604_?->       114797601    SNase-><-SRAP*                                                             -                               110   bacteria>proteobacteria>alphaproteobacteria    Hyphomonas neptunium ATCC 15444                                              hypothetical protein HNE_1448 [Hyphomonas neptunium ATCC 15444].                                                               114797466_?->114798235_?->114798901_SNase-><-114797601_SRAP*||114798675_?-><-114800251_?<-114799458_?       89052729     <-SRAP*||?-><-SNase                                                        -                               210   bacteria>proteobacteria>alphaproteobacteria    Jannaschia sp. CCS1                                                          hypothetical protein Jann_0238 [Jannaschia sp. CCS1].                                                                          89052726_?->89052727_?-><-89052728_?<-89052729_SRAP*||89052730_?-><-89052731_SNase||89052732_?->       319784651    SNase->SRAP*->                                                             -                               236   bacteria>proteobacteria>alphaproteobacteria    Mesorhizobium ciceri biovar biserrulae WSM1271                               hypothetical protein [Mesorhizobium ciceri biovar biserrulae WSM1271].                                                         <-319784648_?||319784649_?->319784650_SNase->319784651_SRAP*-><-319784652_?||319784653_?->319784654_?->       407973439    <-SNase||?->?->SRAP*->                                                     NA8A_04050                      217   bacteria>proteobacteria>alphaproteobacteria    Nitratireductor indicus C115                                                 hypothetical protein NA8A_04050 [Nitratireductor indicus C115].                                                                <-407973436_SNase||407973437_?->407973438_?->407973439_SRAP*->407973440_?->407973441_?->407973442_?->       399065038    <-SNase<-SRAP*                                                             PMI02_04141                     241   bacteria>proteobacteria>alphaproteobacteria    Novosphingobium sp. AP12                                                     hypothetical protein PMI02_04141 [Novosphingobium sp. AP12].                                                                   399065035_?-><-399065036_?<-399065037_SNase<-399065038_SRAP*||399065039_?-><-399065040_?<-399065041_?       444310883    SRAP*-><-SNase                                                             D584_13914                      226   bacteria>proteobacteria>alphaproteobacteria    Ochrobactrum intermedium M86                                                 hypothetical protein D584_13914 [Ochrobactrum intermedium M86].                                                                444310880_?->444310881_?->444310882_?->444310883_SRAP*-><-444310884_SNase<-444310885_?<-444310886_?       478271810    <-SNase||?-><-SRAP*<-SRAP                                                  RHSP_40842                      215   bacteria>proteobacteria>alphaproteobacteria    Rhizobium freirei PRF 81                                                     hypothetical protein RHSP_40842 [Rhizobium freirei PRF 81].                                                                    478271807_?-><-478271808_SNase||478271809_?-><-478271810_SRAP*<-478271811_SRAP||478271812_?-><-478271813_?<-478271814_?       424885130    <-SRAP*<-?<-SNase                                                          Rleg10DRAFT_5625                263   bacteria>proteobacteria>alphaproteobacteria    Rhizobium leguminosarum bv. trifolii WSM2012                                 hypothetical protein Rleg10DRAFT_5625 [Rhizobium leguminosarum bv. trifolii WSM2012].                                          <-424885127_?||424885128_?-><-424885129_?<-424885130_SRAP*<-424885131_?<-424885132_SNase<-424885133_?       116251880    SRAP*->SNase->                                                             -                               257   bacteria>proteobacteria>alphaproteobacteria    Rhizobium leguminosarum bv. viciae 3841                                      hypothetical protein RL2120 [Rhizobium leguminosarum bv. viciae 3841].                                                         <-116251877_?||116251878_?->116251879_?->116251880_SRAP*->116251881_SNase->116251882_?->116251883_?->       209964901    <-SNase<-SRAP*                                                             -                               267   bacteria>proteobacteria>alphaproteobacteria    Rhodospirillum centenum SW                                                   hypothetical protein RC1_1601 [Rhodospirillum centenum SW].                                                                    <-209964898_?||209964899_?-><-209964900_SNase<-209964901_SRAP*||209964902_?->209964903_?->209964904_?->       390166473    <-SNase<-SRAP*                                                             SIDU_04792                      199   bacteria>proteobacteria>alphaproteobacteria    Sphingobium indicum B90A                                                     hypothetical protein SIDU_04792 [Sphingobium indicum B90A].                                                                    390166470_?-><-390166471_?<-390166472_SNase<-390166473_SRAP*<-390166474_?<-390166475_?<-390166476_?       154247665    <-SRAP*<-?||?->SNase->                                                     -                               222   bacteria>proteobacteria>alphaproteobacteria    Xanthobacter autotrophicus Py2                                               hypothetical protein Xaut_3740 [Xanthobacter autotrophicus Py2].                                                               <-154247662_?<-154247663_?<-154247664_?<-154247665_SRAP*<-154247666_?||154247667_?->154247668_SNase->       299529198    SRAP*-><-?<-SNase                                                          CTS44_00484                     221   bacteria>proteobacteria>betaproteobacteria     Comamonas testosteroni S44                                                   hypothetical protein CTS44_00484 [Comamonas testosteroni S44].                                                                 299529195_?->299529196_?-><-299529197_?||299529198_SRAP*-><-299529199_?<-299529200_SNase<-299529201_?       264678762    SNase-><-?<-SRAP*                                                          -                               221   bacteria>proteobacteria>betaproteobacteria     Comamonas testosteroni CNB-2                                                 hypothetical protein CtCNB1_2627 [Comamonas testosteroni CNB-2].                                                               264678759_?->264678760_SNase-><-264678761_?<-264678762_SRAP*<-264678763_?||264678764_?->264678765_?->       253701010    SRAP*->SNase->                                                             -                               221   bacteria>proteobacteria>deltaproteobacteria    Geobacter sp. M21                                                            hypothetical protein GM21_2394 [Geobacter sp. M21].                                                                            253701007_?-><-253701008_?||253701009_?->253701010_SRAP*->253701011_SNase-><-253701012_?<-253701013_?       190576863    SNase-><-SRAP*                                                             pK245.entp27                    223   bacteria>proteobacteria>gammaproteobacteria    Klebsiella pneumoniae                                                        hypothetical protein pK245.entp27 [Klebsiella pneumoniae].                                                                     190576860_?->190576861_?->190576862_SNase-><-190576863_SRAP*||190576864_?-><-190576865_?||190576866_?->        # SSB associated       386775429    SRAP*->?->?->SSB->                                                         BparL_010100016688              227   bacteria>actinobacteria                        Brachybacterium paraconglomeratum LC44                                       hypothetical protein BparL_16688 [Brachybacterium paraconglomeratum LC44].                                                     <-386775426_?<-386775427_?<-386775428_?||386775429_SRAP*->386775430_?->386775431_?->386775432_SSB->       295836592    <-Sigma<-?<-SRAP*                                                          SSBG_02321                      270   bacteria>actinobacteria                        Streptomyces sp. SPB74                                                       conserved hypothetical protein [Streptomyces sp. SPB74].                                                                       <-295836589_?<-295836590_Sigma<-295836591_?<-295836592_SRAP*||295836593_?->295836594_?->295836595_?->       261410088    <-SRAP*||?-><-?<-SSB                                                       -                               224   bacteria>firmicutes                            Paenibacillus sp. Y412MC10                                                   hypothetical protein GYMC10_6319 [Paenibacillus sp. Y412MC10].                                                                 <-261410085_?<-261410086_?<-261410087_?<-261410088_SRAP*||261410089_?-><-261410090_?<-261410091_SSB       315644247    <-SRAP<-SRAP*<-?<-?<-SSB                                                   PVOR_01860                      147   bacteria>firmicutes                            Paenibacillus vortex V453                                                    hypothetical protein PVOR_01860 [Paenibacillus vortex V453].                                                                   <-315644243_?<-315644244_?<-315644245_?<-315644246_SRAP<-315644247_SRAP*<-315644248_?<-315644249_?<-315644250_SSB       442804808    <-SSB<-?<-?<-SRAP*                                                         Cst_c13560                      197   bacteria>firmicutes                            Clostridium stercorarium subsp. stercorarium DSM 8532                        hypothetical protein Cst_c13560 [Clostridium stercorarium subsp. stercorarium DSM 8532].                                       <-442804805_SSB<-442804806_?<-442804807_?<-442804808_SRAP*<-442804809_?<-442804810_?<-442804811_?       329925088    <-SRAP*<-?<-SSB                                                            HMPREF9412_6574                 224   bacteria>firmicutes                            Paenibacillus sp. HGF5                                                       hypothetical protein HMPREF9412_6574 [Paenibacillus sp. HGF5].                                                                 <-329925086_?<-329925117_?<-329925095_?<-329925088_SRAP*<-329925101_?<-329925035_SSB<-329925059_?       354582035    <-SRAP*<-?<-SSB                                                            PaelaDRAFT_2039                 224   bacteria>firmicutes                            Paenibacillus lactis 154                                                     protein of unknown function DUF159 [Paenibacillus lactis 154].                                                                 <-354582032_?<-354582033_?<-354582034_?<-354582035_SRAP*<-354582036_?<-354582037_SSB<-354582038_?       329846742    SRAP*-><-?<-SSB                                                            ABI_00490                       249   bacteria>proteobacteria>alphaproteobacteria    Asticcacaulis biprosthecum C19                                               hypothetical protein ABI_00490 [Asticcacaulis biprosthecum C19].                                                               329846739_?-><-329846740_?||329846741_?->329846742_SRAP*-><-329846743_?<-329846744_SSB||329846745_?->       163858083    transposase->?->SRAP*-><-SSB                                               -                               232   bacteria>proteobacteria>betaproteobacteria     Bordetella petrii DSM 12804                                                  hypothetical protein Bpet3770 [Bordetella petrii DSM 12804].                                                                   163858080_?->163858081_transposase->163858082_?->163858083_SRAP*-><-163858084_SSB<-163858085_?<-163858086_?        # RecG associated        304404158    RecG->?-><-?<-SRAP*                                                        PaecuDRAFT_0479                 227   bacteria>firmicutes                            Paenibacillus curdlanolyticus YK9                                            protein of unknown function DUF159 [Paenibacillus curdlanolyticus YK9].                                                        304404155_RecG->304404156_?-><-304404157_?<-304404158_SRAP*||304404159_?->304404160_?->304404161_?->       261405811    RecG->?-><-SRAP*||?-><-?<-YolD                                             -                               235   bacteria>firmicutes                            Paenibacillus sp. Y412MC10                                                   hypothetical protein GYMC10_1964 [Paenibacillus sp. Y412MC10].                                                                 261405808_?->261405809_RecG->261405810_?-><-261405811_SRAP*||261405812_?-><-261405813_?<-261405814_YolD       315646190    RecG->?->?-><-SRAP*                                                        PVOR_12255                      233   bacteria>firmicutes                            Paenibacillus vortex V453                                                    hypothetical protein PVOR_12255 [Paenibacillus vortex V453].                                                                   315646187_RecG->315646188_?->315646189_?-><-315646190_SRAP*||315646191_?->315646192_?-><-315646193_?       329926599    YolD->?-><-?||SRAP*-><-?<-RecG                                             HMPREF9412_3114                 235   bacteria>firmicutes                            Paenibacillus sp. HGF5                                                       hypothetical protein HMPREF9412_3114 [Paenibacillus sp. HGF5].                                                                 329926595_YolD->329926603_?-><-329926557_?||329926599_SRAP*-><-329926569_?<-329926589_RecG<-329926612_?        # RecA associated       86605785     RecA-><-?<-?<-SRAP*                                                        -                               129   bacteria>cyanobacteria                         Synechococcus sp. JA-3-3Ab                                                   hypothetical protein CYA_1095 [Synechococcus sp. JA-3-3Ab].                                                                    86605782_RecA-><-86605783_?<-86605784_?<-86605785_SRAP*<-86605786_?<-86605787_?<-86605788_?         # Other associations        167041879    SRAP*->                                                                    ALOHA_HF4000133G03ctg1g24       206                                                  uncultured marine microorganism HF4000_133G03                                putative uncharacterized ACR, COG2135 [uncultured marine microorganism HF4000_133G03].                                         <-167041876_?||167041877_?->167041878_?->167041879_SRAP*-><-167041880_?<-167041881_?<-167041882_?       345005481    SRAP*->                                                                    -                               227   archaea                                        halophilic archaeon DL31                                                     hypothetical protein [halophilic archaeon DL31].                                                                               <-345005478_?||345005479_?->345005480_?->345005481_SRAP*-><-345005482_?||345005483_?-><-345005484_?       110668529    <-SRAP*                                                                    HQ2623A                         247   archaea>euryarchaeota                          Haloquadratum walsbyi DSM 16790                                              hypothetical protein HQ2623A [Haloquadratum walsbyi DSM 16790].                                                                <-110668526_?||110668527_?->110668528_?-><-110668529_SRAP*||110668530_?->110668531_?-><-110668532_?       433639624    <-SRAP*                                                                    -                               259   archaea>euryarchaeota                          Halovivax ruber XH-70                                                        hypothetical protein Halru_2675 [Halovivax ruber XH-70].                                                                       <-433639621_?<-433639622_?||433639623_?-><-433639624_SRAP*||433639625_?-><-433639626_?<-433639627_?       15789874     SRAP*->                                                                    VNG0686C                        229   archaea>euryarchaeota                          Halobacterium sp. NRC-1                                                      hypothetical protein VNG0686C [Halobacterium sp. NRC-1].                                                                       15789871_?->15789872_?->15789873_?->15789874_SRAP*->15789875_?->15789876_?->15789877_?->       355570873    <-SRAP*                                                                    MettaDRAFT_0188                 227   archaea>euryarchaeota                          Methanolinea tarda NOBI-1                                                    protein of unknown function DUF159 [Methanolinea tarda NOBI-1].                                                                355570870_?-><-355570871_?||355570872_?-><-355570873_SRAP*<-355570874_?||355570875_?->355570876_?->       55377063     SRAP*->                                                                    -                               233   archaea>euryarchaeota                          Haloarcula marismortui ATCC 43049                                            hypothetical protein rrnAC0135 [Haloarcula marismortui ATCC 43049].                                                            <-55377060_?<-55377061_?||55377062_?->55377063_SRAP*-><-55377064_?||55377065_?-><-55377066_?       257387394    <-SRAP*                                                                    -                               234   archaea>euryarchaeota                          Halomicrobium mukohataei DSM 12286                                           hypothetical protein Hmuk_1339 [Halomicrobium mukohataei DSM 12286].                                                           <-257387391_?<-257387392_?||257387393_?-><-257387394_SRAP*<-257387395_?||257387396_?->257387397_?->       76801924     <-SRAP*                                                                    NP2564A                         233   archaea>euryarchaeota                          Natronomonas pharaonis DSM 2160                                              hypothetical protein NP2564A [Natronomonas pharaonis DSM 2160].                                                                <-76801921_?||76801922_?-><-76801923_?<-76801924_SRAP*||76801925_?-><-76801926_?<-76801927_?       9664591      SRAP*->                                                                    -                               189   archaea>euryarchaeota                          uncultured marine group II euryarchaeote 37F11                               unknown [uncultured marine group II euryarchaeote 37F11].                                                                      9664588_?->9664589_?->9664590_?->9664591_SRAP*->9664612_?->9664592_?->9664593_?->       448409258    SRAP*->                                                                    C475_09924                      238   archaea>euryarchaeota                          Halosimplex carlsbadense 2-9-1                                               hypothetical protein C475_09924 [Halosimplex carlsbadense 2-9-1].                                                              448409255_?->448409256_?->448409257_?->448409258_SRAP*->448409259_?->448409260_?-><-448409261_?       448349130    SRAP*->                                                                    C484_06232                      277   archaea>euryarchaeota                          Natrialba taiwanensis DSM 12281                                              hypothetical protein C484_06232 [Natrialba taiwanensis DSM 12281].                                                             448349127_?->448349128_?-><-448349129_?||448349130_SRAP*->448349131_?-><-448349132_?||448349133_?->       448335931    SRAP*->                                                                    C488_20987                      285   archaea>euryarchaeota                          Natrinema pellirubrum DSM 15624                                              hypothetical protein C488_20987 [Natrinema pellirubrum DSM 15624].                                                             <-448335928_?<-448335929_?<-448335930_?||448335931_SRAP*-><-448335932_?       448315324    SRAP*->                                                                    C492_02959                      280   archaea>euryarchaeota                          Natronococcus jeotgali DSM 18795                                             hypothetical protein C492_02959 [Natronococcus jeotgali DSM 18795].                                                            448315321_?->448315322_?->448315323_?->448315324_SRAP*-><-448315325_?||448315326_?->448315327_?->       307353128    <-SRAP*                                                                    -                               225   archaea>euryarchaeota                          Methanoplanus petrolearius DSM 11571                                         hypothetical protein Mpet_0974 [Methanoplanus petrolearius DSM 11571].                                                         307353125_?->307353126_?->307353127_?-><-307353128_SRAP*<-307353129_?<-307353130_?<-307353131_?       354611648    <-SRAP*                                                                    -                               229   archaea>euryarchaeota                          Halobacterium sp. DL1                                                        protein of unknown function DUF159 [Halobacterium sp. DL1].                                                                    354611645_?-><-354611646_?<-354611647_?<-354611648_SRAP*<-354611649_?<-354611650_?<-354611651_?       374724759    SRAP*->                                                                    MG2_1251                        223   archaea>euryarchaeota                          uncultured marine group II euryarchaeote                                     hypothetical protein MG2_1251 [uncultured marine group II euryarchaeote].                                                      374724755_?->374724757_?->374724758_?->374724759_SRAP*-><-374724760_?||374724761_?->374724762_?->       448418454    <-SRAP*                                                                    C474_13519                      69    archaea>euryarchaeota                          Halosarcina pallida JCM 14848                                                hypothetical protein C474_13519 [Halosarcina pallida JCM 14848].                                                               448418451_?-><-448418452_?||448418453_?-><-448418454_SRAP*||448418455_?-><-448418456_?<-448418457_?       448473509    <-SRAP*                                                                    C461_04547                      259   archaea>euryarchaeota                          Halorubrum aidingense JCM 13560                                              hypothetical protein C461_04547 [Halorubrum aidingense JCM 13560].                                                             448473506_?->448473507_?->448473508_?-><-448473509_SRAP*||448473510_?-><-448473511_?||448473512_?->       448730217    <-SRAP*                                                                    C449_10538                      235   archaea>euryarchaeota                          Halococcus saccharolyticus DSM 5350                                          hypothetical protein C449_10538 [Halococcus saccharolyticus DSM 5350].                                                         448730214_?->448730215_?->448730216_?-><-448730217_SRAP*||448730218_?->448730219_?->448730220_?->       432331616    SRAP*->                                                                    -                               244   archaea>euryarchaeota                          Methanoregula formicicum SMSP                                                hypothetical protein Metfor_2247 [Methanoregula formicicum SMSP].                                                              432331613_?-><-432331614_?<-432331615_?||432331616_SRAP*-><-432331617_?<-432331618_?<-432331619_?       257053446    SRAP*->                                                                    -                               233   archaea>euryarchaeota                          Halorhabdus utahensis DSM 12940                                              hypothetical protein Huta_2380 [Halorhabdus utahensis DSM 12940].                                                              257053443_?->257053444_?-><-257053445_?||257053446_SRAP*-><-257053447_?<-257053448_?||257053449_?->       452208077    <-SRAP*                                                                    Nmlp_3383                       228   archaea>euryarchaeota                          Natronomonas moolapensis 8.8.11                                              UPF0361 family protein [Natronomonas moolapensis 8.8.11].                                                                      452208074_?-><-452208075_?<-452208076_?<-452208077_SRAP*||452208078_?->452208079_?->452208080_?->       322367948    SRAP*->                                                                    ZOD2009_00660                   226   archaea>euryarchaeota                          Haladaptatus paucihalophilus DX253                                           hypothetical protein ZOD2009_00660 [Haladaptatus paucihalophilus DX253].                                                       <-322367945_?<-322367946_?||322367947_?->322367948_SRAP*->322367949_?-><-322367950_?||322367951_?->       300710561    SRAP*->                                                                    HacjB3_05960                    222   archaea>euryarchaeota                          Halalkalicoccus jeotgali B3                                                  hypothetical protein HacjB3_05960 [Halalkalicoccus jeotgali B3].                                                               <-300710558_?<-300710559_?<-300710560_?||300710561_SRAP*->300710562_?->300710563_?-><-300710564_?       88602717     SRAP*->                                                                    -                               218   archaea>euryarchaeota                          Methanospirillum hungatei JF-1                                               hypothetical protein Mhun_1441 [Methanospirillum hungatei JF-1].                                                               88602714_?->88602715_?->88602716_?->88602717_SRAP*-><-88602718_?||88602719_?-><-88602720_?       397773966    <-SRAP*                                                                    NJ7G_2198                       360   archaea>euryarchaeota                          Natrinema sp. J7-2                                                           hypothetical protein NJ7G_2198 [Natrinema sp. J7-2].                                                                           <-397773963_?<-397773964_?<-397773965_?<-397773966_SRAP*<-397773967_?<-397773968_?<-397773969_?       219853176    SRAP*->                                                                    -                               220   archaea>euryarchaeota                          Methanosphaerula palustris E1-9c                                             hypothetical protein Mpal_2616 [Methanosphaerula palustris E1-9c].                                                             219853173_?-><-219853174_?||219853175_?->219853176_SRAP*-><-219853177_?<-219853178_?||219853179_?->       399574367    <-SRAP*                                                                    HSB1_01650                      237   archaea>euryarchaeota                          Halogranum salarium B-1                                                      hypothetical protein HSB1_01650 [Halogranum salarium B-1].                                                                     399574364_?->399574365_?->399574366_?-><-399574367_SRAP*||399574368_?->399574369_?->399574370_?->       406976458    -                                                                          ACD_23C00221G0005               87    bacteria                                       uncultured bacterium                                                         hypothetical protein ACD_23C00221G0005, partial [uncultured bacterium].                                                               40062519     <-SRAP*                                                                    MBMO_EBAC750-01B07.6            244   bacteria                                       uncultured marine bacterium 106                                              conserved hypothetical protein [uncultured marine bacterium 106].                                                              40062516_?->40062517_?->40062518_?-><-40062519_SRAP*<-40062520_?<-40062521_?<-40062522_?       255671637    <-SRAP*                                                                    -                               237   bacteria                                       uncultured bacterium HF186_25m_30B18                                         uncharacterized conserved protein [uncultured bacterium HF186_25m_30B18].                                                      255671634_?->255671635_?-><-255671636_?<-255671637_SRAP*||255671638_?-><-255671639_?||255671640_?->       406976770    -                                                                          ACD_23C00122G0002               48    bacteria                                       uncultured bacterium                                                         site-specific DNA-methyltransferase, partial [uncultured bacterium].                                                                  218960390    SRAP*->                                                                    CLOAM0040                       240   bacteria                                       Candidatus Cloacamonas acidaminovorans str. Evry                             conserved hypothetical protein [Candidatus Cloacamonas acidaminovorans str. Evry].                                             218960387_?->218960388_?-><-218960389_?||218960390_SRAP*->218960391_?->218960392_?->218960393_?->       406961473    SRAP*->                                                                    ACD_35C00019G0001               103   bacteria                                       uncultured bacterium                                                         hypothetical protein ACD_35C00019G0001, partial [uncultured bacterium].                                                        406961473_SRAP*->       406991541    SRAP*->                                                                    ACD_15C00151G0011               219   bacteria                                       uncultured bacterium                                                         hypothetical protein ACD_15C00151G0011 [uncultured bacterium].                                                                 406991538_?->406991539_?-><-406991540_?||406991541_SRAP*->406991542_?->406991543_?-><-406991544_?       407007433    SRAP*->                                                                    ACD_6C00604G0002                220   bacteria                                       uncultured bacterium                                                         hypothetical protein ACD_6C00604G0002 [uncultured bacterium].                                                                  407007432_?->407007433_SRAP*-><-407007434_?||407007435_?-><-407007436_?       406883828    <-SRAP*                                                                    ACD_77C00345G0010               231   bacteria                                       uncultured bacterium                                                         hypothetical protein ACD_77C00345G0010 [uncultured bacterium].                                                                 406883825_?-><-406883826_?||406883827_?-><-406883828_SRAP*<-406883829_?<-406883830_?<-406883831_?       406999697    <-SRAP*                                                                    ACD_10C00547G0003               214   bacteria                                       uncultured bacterium                                                         hypothetical protein ACD_10C00547G0003 [uncultured bacterium].                                                                 406999695_?->406999696_?-><-406999697_SRAP*       406976456    <-SRAP*<-?<-SRAP                                                           ACD_23C00221G0003               77    bacteria                                       uncultured bacterium                                                         hypothetical protein ACD_23C00221G0003 [uncultured bacterium].                                                                 <-406976454_?||406976455_?-><-406976456_SRAP*<-406976457_?<-406976458_SRAP       335428033    SRAP*->                                                                    HLPCO_03715                     228   bacteria                                       Haloplasma contractile SSD-17B                                               hypothetical protein HLPCO_03715 [Haloplasma contractile SSD-17B].                                                             <-335428030_?<-335428031_?<-335428032_?||335428033_SRAP*->335428034_?-><-335428035_?<-335428036_?       451982528    SRAP*->                                                                    NITGR_980005                    221   bacteria                                       Nitrospina gracilis 3/211                                                    conserved hypothetical protein [Nitrospina gracilis 3/211].                                                                    451982525_?->451982526_?-><-451982527_?||451982528_SRAP*-><-451982529_?<-451982530_?<-451982531_?       397690015    SRAP*->                                                                    MROS_1016                       230   bacteria                                       Melioribacter roseus P3M-2                                                   hypothetical protein MROS_1016 [Melioribacter roseus P3M-2].                                                                   <-397690012_?||397690013_?-><-397690014_?||397690015_SRAP*->397690016_?->397690017_?-><-397690018_?       406921878    SRAP*->SRAP->                                                              ACD_54C01263G0008               226   bacteria                                       uncultured bacterium                                                         protein of unknown function DUF159 [uncultured bacterium].                                                                     406921875_?->406921876_?-><-406921877_?||406921878_SRAP*->406921879_SRAP->       406976769    SRAP*->SRAP->                                                              ACD_23C00122G0001               213   bacteria                                       uncultured bacterium                                                         hypothetical protein ACD_23C00122G0001 [uncultured bacterium].                                                                 406976769_SRAP*->406976770_SRAP->       68305011     <-SRAP*                                                                    -                               192   bacteria                                       uncultured bacterium BAC13K9BAC                                              hypothetical protein all3194 [uncultured bacterium BAC13K9BAC].                                                                68305008_?->68305009_?->68305010_?-><-68305011_SRAP*<-68305012_?<-68305013_?<-68305014_?       269839147    <-SRAP*                                                                    Tter_2115                       213   bacteria                                       Thermobaculum terrenum ATCC BAA-798                                          hypothetical protein Tter_2115 [Thermobaculum terrenum ATCC BAA-798].                                                          <-269839144_?||269839145_?-><-269839146_?<-269839147_SRAP*<-269839148_?<-269839149_?<-269839150_?       406923587    <-SRAP*                                                                    ACD_54C00624G0001               142   bacteria                                       uncultured bacterium                                                         hypothetical protein ACD_54C00624G0001 [uncultured bacterium].                                                                 <-406923587_SRAP*<-406923588_?       406921879    -                                                                          ACD_54C01263G0009               55    bacteria                                       uncultured bacterium                                                         hypothetical protein ACD_54C01263G0009, partial [uncultured bacterium].                                                               390958104    <-SRAP*                                                                    Terro_2262                      237   bacteria>acidobacteria                         Terriglobus roseus DSM 18391                                                 hypothetical protein Terro_2262 [Terriglobus roseus DSM 18391].                                                                390958101_?->390958102_?->390958103_?-><-390958104_SRAP*<-390958105_?<-390958106_?<-390958107_?       347756740    <-SRAP*                                                                    Cabther_B0789                   253   bacteria>acidobacteria                         Candidatus Chloracidobacterium thermophilum B                                hypothetical protein [Candidatus Chloracidobacterium thermophilum B].                                                          <-347756737_?<-347756738_?<-347756739_?<-347756740_SRAP*<-347756741_?<-347756742_?||347756743_?->       322433257    SRAP*->ArsR->                                                              AciX9_4400                      222   bacteria>acidobacteria                         Granulicella tundricola MP5ACTX9                                             hypothetical protein AciX9_4400 [Granulicella tundricola MP5ACTX9].                                                            <-322433254_?<-322433255_?||322433256_?->322433257_SRAP*->322433258_ArsR->322433259_?->322433260_?->       94970917     <-SRAP*                                                                    -                               235   bacteria>acidobacteria                         Candidatus Koribacter versatilis Ellin345                                    hypothetical protein Acid345_3891 [Candidatus Koribacter versatilis Ellin345].                                                 <-94970914_?<-94970915_?<-94970916_?<-94970917_SRAP*||94970918_?-><-94970919_?<-94970920_?       390956113    <-SRAP*                                                                    Terro_0184                      224   bacteria>acidobacteria                         Terriglobus roseus DSM 18391                                                 hypothetical protein Terro_0184 [Terriglobus roseus DSM 18391].                                                                390956110_?-><-390956111_?<-390956112_?<-390956113_SRAP*<-390956114_?<-390956115_?||390956116_?->       374310400    <-SRAP*                                                                    -                               248   bacteria>acidobacteria                         Granulicella mallensis MP5ACTX8                                              hypothetical protein [Granulicella mallensis MP5ACTX8].                                                                        <-374310397_?<-374310398_?<-374310399_?<-374310400_SRAP*<-374310401_?<-374310402_?||374310403_?->       459053731    <-SRAP*<-?||Sigma->                                                        GP2_012_00890                   251   bacteria>actinobacteria                        Gordonia paraffinivorans NBRC 108238                                         hypothetical protein GP2_012_00890 [Gordonia paraffinivorans NBRC 108238].                                                     <-459053728_?<-459053729_?||459053730_?-><-459053731_SRAP*<-459053732_?||459053733_Sigma->459053734_?->       297626216    SRAP*->Sigma->                                                             PFREUD_10190                    250   bacteria>actinobacteria                        Propionibacterium freudenreichii subsp. shermanii CIRM-BIA1                  hypothetical protein PFREUD_10190 [Propionibacterium freudenreichii subsp. shermanii CIRM-BIA1].                               <-297626213_?<-297626214_?<-297626215_?||297626216_SRAP*->297626217_Sigma->297626218_?-><-297626219_?       358445603    SRAP*->Sigma->                                                             CCAS_04395                      216   bacteria>actinobacteria                        Corynebacterium casei UCMA 3821                                              putative uncharacterized protein [Corynebacterium casei UCMA 3821].                                                            358445600_?-><-358445601_?<-358445602_?||358445603_SRAP*->358445604_Sigma->358445605_?-><-358445606_?       403720914    <-SRAP*<-?||Sigma->                                                        GORHZ_028_00190                 270   bacteria>actinobacteria                        Gordonia rhizosphera NBRC 16068                                              hypothetical protein GORHZ_028_00190 [Gordonia rhizosphera NBRC 16068].                                                        <-403720911_?||403720912_?->403720913_?-><-403720914_SRAP*<-403720915_?||403720916_Sigma->403720917_?->       317483272    -                                                                          HMPREF0177_01662                118   bacteria>actinobacteria                        Bifidobacterium sp. 12_1_47BFAA                                              integrase, partial [Bifidobacterium sp. 12_1_47BFAA].                                                                                 288923104    <-SRAP*<-?||GNAT->                                                         FrEUN1fDRAFT_6951               312   bacteria>actinobacteria                        Frankia sp. EUN1f                                                            protein of unknown function DUF159 [Frankia sp. EUN1f].                                                                        <-288923101_?||288923102_?-><-288923103_?<-288923104_SRAP*<-288923105_?||288923106_GNAT->288923107_?->       334563621    SRAP*->Sigma->                                                             CbovD2_010100003529             261   bacteria>actinobacteria                        Corynebacterium bovis DSM 20582                                              hypothetical protein CbovD2_03529, partial [Corynebacterium bovis DSM 20582].                                                  334563621_SRAP*->334563622_Sigma->334563623_?-><-334563624_?       358459823    <-SRAP*<-?||GNAT->                                                         FrCN3DRAFT_4688                 301   bacteria>actinobacteria                        Frankia sp. CN3                                                              protein of unknown function DUF159 [Frankia sp. CN3].                                                                          358459820_?-><-358459821_?||358459822_?-><-358459823_SRAP*<-358459824_?||358459825_GNAT->358459826_?->       302532667    <-SRAP*<-?<-SRAP                                                           SSNG_00630                      248   bacteria>actinobacteria                        Streptomyces sp. C                                                           conserved hypothetical protein [Streptomyces sp. C].                                                                           <-302532664_?||302532665_?->302532666_?-><-302532667_SRAP*<-302532668_?<-302532669_SRAP||302532670_?->       441507527    <-SRAP*<-?||Sigma->                                                        GOACH_03_04340                  269   bacteria>actinobacteria                        Gordonia aichiensis NBRC 108223                                              hypothetical protein GOACH_03_04340 [Gordonia aichiensis NBRC 108223].                                                         441507524_?-><-441507525_?||441507526_?-><-441507527_SRAP*<-441507528_?||441507529_Sigma->441507530_?->       306835497    -                                                                          -                               294   bacteria>actinobacteria                        Corynebacterium accolens ATCC 49726                                          GTPase, partial [Corynebacterium accolens ATCC 49726].                                                                                404421783    -                                                                          MFORT_15192                     83    bacteria>actinobacteria                        Mycobacterium fortuitum subsp. fortuitum DSM 46621                           3-phosphoshikimate 1-carboxyvinyltransferase, partial [Mycobacterium fortuitum subsp. fortuitum DSM 46621].                           311064481    SRAP*->                                                                    BBPR_1104                       231   bacteria>actinobacteria                        Bifidobacterium bifidum PRL2010                                              hypothetical protein BBPR_1104 [Bifidobacterium bifidum PRL2010].                                                              <-311064478_?||311064479_?->311064480_?->311064481_SRAP*->311064482_?-><-311064483_?||311064484_?->       291454044    SRAP*->?->Sigma->                                                          SSHG_04337                      290   bacteria>actinobacteria                        Streptomyces albus J1074                                                     conserved hypothetical protein [Streptomyces albus J1074].                                                                     <-291454041_?<-291454042_?<-291454043_?||291454044_SRAP*->291454045_?->291454046_Sigma->291454047_?->       310287566    SRAP*->                                                                    BBIF_1045                       231   bacteria>actinobacteria                        Bifidobacterium bifidum S17                                                  hypothetical protein BBIF_1045 [Bifidobacterium bifidum S17].                                                                  <-310287563_?<-310287564_?<-310287565_?||310287566_SRAP*->310287567_?-><-310287568_?||310287569_?->       302532669    -                                                                          SSNG_00632                      191   bacteria>actinobacteria                        Streptomyces sp. C                                                           two component system response regulator, partial [Streptomyces sp. C].                                                                15610362     <-SRAP*                                                                    -                               252   bacteria>actinobacteria                        Mycobacterium tuberculosis H37Rv                                             Conserved hypothetical protein [Mycobacterium tuberculosis H37Rv].                                                             57117076_?->57117077_?-><-15610361_?<-15610362_SRAP*||15610363_?->15610364_?-><-57117078_?       410867822    SRAP*->                                                                    PACID_33200                     241   bacteria>actinobacteria                        Propionibacterium acidipropionici ATCC 4875                                  hypothetical protein PACID_33200 [Propionibacterium acidipropionici ATCC 4875].                                                410867819_?->410867820_?->410867821_?->410867822_SRAP*-><-410867823_?<-410867824_?<-410867825_?       329939683    SRAP*->?->Sigma->                                                          SGM_4476                        242   bacteria>actinobacteria                        Streptomyces griseoaurantiacus M045                                          hypothetical protein SGM_4476 [Streptomyces griseoaurantiacus M045].                                                           <-329939680_?<-329939681_?<-329939682_?||329939683_SRAP*->329939684_?->329939685_Sigma->329939686_?->       381397289    <-SRAP*                                                                    OR221_1246                      236   bacteria>actinobacteria                        Microbacterium laevaniformans OR221                                          protein of unknown function DUF159 [Microbacterium laevaniformans OR221].                                                      381397286_?-><-381397287_?||381397288_?-><-381397289_SRAP*<-381397290_?<-381397291_?||381397292_?->       479321619    SRAP*->?->Sigma->                                                          -                               252   bacteria>actinobacteria                        Streptomyces sp. PAMC26508                                                   hypothetical protein F750_4747 [Streptomyces sp. PAMC26508].                                                                   <-479321616_?<-479321617_?<-479321618_?||479321619_SRAP*->479321620_?->479321621_Sigma->479321622_?->       239990616    SRAP*->?->Sigma->                                                          SrosN1_010100025140             271   bacteria>actinobacteria                        Streptomyces roseosporus NRRL 11379                                          hypothetical protein SrosN1_25140 [Streptomyces roseosporus NRRL 11379].                                                       <-239990613_?<-239990614_?<-239990615_?||239990616_SRAP*->239990617_?->239990618_Sigma->239990619_?->       448823158    SRAP*->Sigma->                                                             CU7111_0484                     235   bacteria>actinobacteria                        Corynebacterium urealyticum DSM 7111                                         hypothetical protein CU7111_0484 [Corynebacterium urealyticum DSM 7111].                                                       448823155_?-><-448823156_?<-448823157_?||448823158_SRAP*->448823159_Sigma->448823160_?-><-448823161_?       386842409    SRAP*->?->Sigma->                                                          -                               237   bacteria>actinobacteria                        Streptomyces hygroscopicus subsp. jinggangensis 5008                         hypothetical protein SHJG_6327 [Streptomyces hygroscopicus subsp. jinggangensis 5008].                                         <-386842406_?<-386842407_?||386842408_?->386842409_SRAP*->386842410_?->386842411_Sigma->386842412_?->       357401653    SRAP*->?->Sigma->                                                          -                               281   bacteria>actinobacteria                        Streptomyces cattleya NRRL 8057 = DSM 46488                                  hypothetical protein SCAT_4079 [Streptomyces cattleya NRRL 8057 = DSM 46488].                                                  <-357401650_?<-357401651_?<-357401652_?||357401653_SRAP*->357401654_?->357401655_Sigma->357401656_?->       345848572    SRAP*->?->Sigma->                                                          SZN_02597                       271   bacteria>actinobacteria                        Streptomyces zinciresistens K42                                              hypothetical protein SZN_02597 [Streptomyces zinciresistens K42].                                                              <-345848569_?||345848570_?->345848571_?->345848572_SRAP*->345848573_?->345848574_Sigma->345848575_?->       477579021    -                                                                          D477_011701                     39    bacteria>actinobacteria                        Arthrobacter crystallopoietes BAB-32                                         Zn-dependent alcohol dehydrogenase, partial [Arthrobacter crystallopoietes BAB-32].                                                   458871330    SRAP*->?->Sigma->                                                          H114_23601                      271   bacteria>actinobacteria                        Streptomyces gancidicus BKS 13-15                                            hypothetical protein H114_23601 [Streptomyces gancidicus BKS 13-15].                                                           <-458871327_?<-458871328_?<-458871329_?||458871330_SRAP*->458871331_?->458871332_Sigma->458871333_?->       408826308    SRAP*->?->Sigma->                                                          SsomD4_010100003909             271   bacteria>actinobacteria                        Streptomyces somaliensis DSM 40738                                           hypothetical protein SsomD4_03909 [Streptomyces somaliensis DSM 40738].                                                        <-408826305_?<-408826306_?<-408826307_?||408826308_SRAP*->408826309_?->408826310_Sigma->408826311_?->       375137584    <-SRAP*                                                                    -                               266   bacteria>actinobacteria                        Mycobacterium rhodesiae NBB3                                                 hypothetical protein [Mycobacterium rhodesiae NBB3].                                                                           <-375137581_?<-375137582_?<-375137583_?<-375137584_SRAP*||375137585_?->375137586_?->375137587_?->       383808179    <-SRAP*                                                                    HMPREF1324_0945                 342   bacteria>actinobacteria                        Rothia aeria F0474                                                           hypothetical protein HMPREF1324_0945 [Rothia aeria F0474].                                                                     <-383808002_?<-383808053_?<-383808139_?<-383808179_SRAP*<-383808004_?||383807995_?-><-383808075_?       383818045    <-SRAP*                                                                    MPHLEI_02143                    249   bacteria>actinobacteria                        Mycobacterium phlei RIVM601174                                               hypothetical protein MPHLEI_02143 [Mycobacterium phlei RIVM601174].                                                            <-383818042_?<-383818043_?<-383818044_?<-383818045_SRAP*||383818046_?->383818047_?-><-383818048_?       359770360    <-SRAP*<-aminoacyl-tRNA_deacylase||Sigma->                                 GOEFS_008_00270                 269   bacteria>actinobacteria                        Gordonia effusa NBRC 100432                                                  hypothetical protein GOEFS_008_00270 [Gordonia effusa NBRC 100432].                                                            <-359770357_?<-359770358_?<-359770359_?<-359770360_SRAP*<-359770361_aminoacyl-tRNA_deacylase||359770362_Sigma->359770363_?->       350568483    SRAP*->                                                                    -                               155   bacteria>actinobacteria                        Propionibacterium avidum ATCC 25577                                          hypothetical protein HMPREF9153_0966 [Propionibacterium avidum ATCC 25577].                                                    350568480_?->350568481_?-><-350568482_?||350568483_SRAP*-><-350568484_?<-350568485_?<-350568486_?       479316215    -                                                                          -                               83    bacteria>actinobacteria                        Mycobacterium tuberculosis str. Beijing/NITR203                              hypothetical protein J112_17340 [Mycobacterium tuberculosis str. Beijing/NITR203].                                                    403251009    -                                                                          A27L6_005800000040              467   bacteria>actinobacteria                        actinobacterium SCGC AAA027-L06                                              DNA polymerase III, alpha subunit, partial [actinobacterium SCGC AAA027-L06].                                                         291437363    -                                                                          SSFG_02463                      144   bacteria>actinobacteria                        Streptomyces ghanaensis ATCC 14672                                           LOW QUALITY PROTEIN: conserved hypothetical protein, partial [Streptomyces ghanaensis ATCC 14672].                                    408680586    SRAP*->?->Sigma->                                                          SVEN_4868                       271   bacteria>actinobacteria                        Streptomyces venezuelae ATCC 10712                                           hypothetical protein SVEN_4868 [Streptomyces venezuelae ATCC 10712].                                                           <-408680583_?<-408680584_?<-408680585_?||408680586_SRAP*->408680587_?->408680588_Sigma->408680589_?->       365866736    SRAP*->?->Sigma->                                                          SPW_6644                        271   bacteria>actinobacteria                        Streptomyces sp. W007                                                        hypothetical protein SPW_6644 [Streptomyces sp. W007].                                                                         <-365866733_?<-365866734_?<-365866735_?||365866736_SRAP*->365866737_?->365866738_Sigma->365866739_?->       419968579    -                                                                          WSS_A40400                      112   bacteria>actinobacteria                        Rhodococcus opacus M213                                                      hypothetical protein WSS_A40400 [Rhodococcus opacus M213].                                                                            419968581    -                                                                          WSS_A40420                      39    bacteria>actinobacteria                        Rhodococcus opacus M213                                                      putative membrane protein, partial [Rhodococcus opacus M213].                                                                         134102868    -                                                                          -                               252   bacteria>actinobacteria                        Saccharopolyspora erythraea NRRL 2338                                        bacteriophage protein [Saccharopolyspora erythraea NRRL 2338].                                                                        254384630    SRAP*->?->Sigma->                                                          SSAG_04354                      271   bacteria>actinobacteria                        Streptomyces sp. Mg1                                                         conserved hypothetical protein [Streptomyces sp. Mg1].                                                                         <-254384627_?<-254384628_?<-254384629_?||254384630_SRAP*->254384631_?->254384632_Sigma->254384633_?->       386383373    <-SRAP*                                                                    STSU_10836                      257   bacteria>actinobacteria                        Streptomyces tsukubaensis NRRL18488                                          hypothetical protein STSU_10836 [Streptomyces tsukubaensis NRRL18488].                                                         <-386383373_SRAP*||386383374_?->386383375_?->386383376_?->       297204781    SRAP*->                                                                    SSEG_03100                      247   bacteria>actinobacteria                        Streptomyces sviceus ATCC 29083                                              conserved hypothetical protein [Streptomyces sviceus ATCC 29083].                                                              <-297204778_?||297204779_?-><-297204780_?||297204781_SRAP*-><-297204782_?<-297204783_?||297204784_?->       254388977    SRAP*->?->Sigma->                                                          SSCG_01395                      274   bacteria>actinobacteria                        Streptomyces clavuligerus ATCC 27064                                         conserved hypothetical protein [Streptomyces clavuligerus ATCC 27064].                                                         <-254388974_?<-254388975_?<-254388976_?||254388977_SRAP*->254388978_?->254388979_Sigma->254388980_?->       421736830    -                                                                          B217_06702                      150   bacteria>actinobacteria                        Bifidobacterium bifidum IPLA 20015                                           integral membrane sensor signal transduction histidine kinase, partial [Bifidobacterium bifidum IPLA 20015].                          294631315    SRAP*->?->Sigma->                                                          SSTG_03316                      271   bacteria>actinobacteria                        Streptomyces sp. e14                                                         conserved hypothetical protein [Streptomyces sp. e14].                                                                         <-294631312_?<-294631313_?<-294631314_?||294631315_SRAP*->294631316_?->294631317_Sigma->294631318_?->       345009200    SRAP*->?->Sigma->                                                          -                               271   bacteria>actinobacteria                        Streptomyces violaceusniger Tu 4113                                          hypothetical protein [Streptomyces violaceusniger Tu 4113].                                                                    <-345009197_?<-345009198_?<-345009199_?||345009200_SRAP*->345009201_?->345009202_Sigma->345009203_?->       302554033    SRAP*->?->Sigma->                                                          SSQG_05262                      271   bacteria>actinobacteria                        Streptomyces viridochromogenes DSM 40736                                     conserved hypothetical protein [Streptomyces viridochromogenes DSM 40736].                                                     <-302554030_?<-302554031_?<-302554032_?||302554033_SRAP*->302554034_?->302554035_Sigma->302554036_?->       345001967    SRAP*->?->Sigma->                                                          -                               271   bacteria>actinobacteria                        Streptomyces sp. SirexAA-E                                                   hypothetical protein SACTE_4451 [Streptomyces sp. SirexAA-E].                                                                  <-345001964_?<-345001965_?||345001966_?->345001967_SRAP*->345001968_?->345001969_Sigma->345001970_?->       354615939    SRAP*->SRAP->                                                              SacpaDRAFT_3054                 264   bacteria>actinobacteria                        Saccharomonospora paurometabolica YIM 90007                                  protein of unknown function DUF159 [Saccharomonospora paurometabolica YIM 90007].                                              354615936_?-><-354615937_?<-354615938_?||354615939_SRAP*->354615940_SRAP->       318079741    SRAP*->?->Sigma->                                                          SSA3_010100024411               238   bacteria>actinobacteria                        Streptomyces sp. SA3_actF                                                    hypothetical protein SSA3_24411, partial [Streptomyces sp. SA3_actF].                                                          318079741_SRAP*->318079742_?->318079743_Sigma->318079744_?->       302521835    SRAP*->?->Sigma->                                                          SSLG_04604                      270   bacteria>actinobacteria                        Streptomyces sp. SPB78                                                       conserved hypothetical protein [Streptomyces sp. SPB78].                                                                       <-302521832_?<-302521833_?<-302521834_?||302521835_SRAP*->302521836_?->302521837_Sigma->302521838_?->       21223582     SRAP*->?->Sigma->                                                          -                               271   bacteria>actinobacteria                        Streptomyces coelicolor A3(2)                                                hypothetical protein SCO5214 [Streptomyces coelicolor A3(2)].                                                                  <-21223579_?<-21223580_?<-21223581_?||21223582_SRAP*->21223583_?->21223584_Sigma->21223585_?->       418471824    SRAP*->?->Sigma->                                                          SMCF_4596                       271   bacteria>actinobacteria                        Streptomyces coelicoflavus ZG0656                                            hypothetical protein SMCF_4596 [Streptomyces coelicoflavus ZG0656].                                                            <-418471820_?<-418471821_?<-418471822_?||418471824_SRAP*->418471825_?->418471826_Sigma->418471827_?->       417748929    SRAP*->                                                                    MAPs_02260                      257   bacteria>actinobacteria                        Mycobacterium avium subsp. paratuberculosis S397                             hypothetical protein MAPs_02260 [Mycobacterium avium subsp. paratuberculosis S397].                                            417748926_?-><-417748927_?<-417748928_?||417748929_SRAP*->       117927744    SRAP*->?->Sigma->                                                          -                               250   bacteria>actinobacteria                        Acidothermus cellulolyticus 11B                                              hypothetical protein Acel_0536 [Acidothermus cellulolyticus 11B].                                                              <-117927741_?<-117927742_?<-117927743_?||117927744_SRAP*->117927745_?->117927746_Sigma->117927747_?->       297202268    SRAP*->?->Sigma->                                                          SSEG_00829                      274   bacteria>actinobacteria                        Streptomyces sviceus ATCC 29083                                              conserved hypothetical protein [Streptomyces sviceus ATCC 29083].                                                              <-297202265_?<-297202266_?<-297202267_?||297202268_SRAP*->297202269_?->297202270_Sigma->297202271_?->       458972095    SRAP*->?->Sigma->                                                          H340_00305                      272   bacteria>actinobacteria                        Streptomyces mobaraensis NBRC 13819 = DSM 40847                              hypothetical protein H340_00305 [Streptomyces mobaraensis NBRC 13819 = DSM 40847].                                             <-458972092_?<-458972093_?<-458972094_?||458972095_SRAP*->458972096_?->458972097_Sigma->458972098_?->       421744315    SRAP*->?->Sigma->                                                          SM8_06008                       290   bacteria>actinobacteria                        Streptomyces sp. SM8                                                         hypothetical protein SM8_06008 [Streptomyces sp. SM8].                                                                         <-421744312_?<-421744313_?<-421744314_?||421744315_SRAP*->421744316_?->421744317_Sigma->421744318_?->       302536797    SRAP*->?->Sigma->                                                          SSNG_04760                      271   bacteria>actinobacteria                        Streptomyces sp. C                                                           conserved hypothetical protein [Streptomyces sp. C].                                                                           <-302536794_?<-302536795_?<-302536796_?||302536797_SRAP*->302536798_?->302536799_Sigma->302536800_?->       363422343    <-SRAP*                                                                    AK37_16870                      272   bacteria>actinobacteria                        Rhodococcus pyridinivorans AK37                                              hypothetical protein AK37_16870 [Rhodococcus pyridinivorans AK37].                                                             363422340_?-><-363422341_?<-363422342_?<-363422343_SRAP*<-363422344_?<-363422345_?<-363422346_?       315503815    SRAP*->?->?->Sigma->                                                       -                               235   bacteria>actinobacteria                        Micromonospora sp. L5                                                        hypothetical protein ML5_3034 [Micromonospora sp. L5].                                                                         <-315503812_?<-315503813_?<-315503814_?||315503815_SRAP*->315503816_?->315503817_?->315503818_Sigma->       387877413    <-SRAP*                                                                    -                               254   bacteria>actinobacteria                        Mycobacterium sp. MOTT36Y                                                    hypothetical protein W7S_20195 [Mycobacterium sp. MOTT36Y].                                                                    387877410_?->387877411_?-><-387877412_?<-387877413_SRAP*||387877414_?->387877415_?-><-387877416_?       296138828    SRAP*->                                                                    Tpau_1100                       254   bacteria>actinobacteria                        Tsukamurella paurometabola DSM 20162                                         hypothetical protein [Tsukamurella paurometabola DSM 20162].                                                                   <-296138825_?||296138826_?->296138827_?->296138828_SRAP*-><-296138829_?||296138830_?-><-296138831_?       418047483    SRAP*->                                                                    -                               251   bacteria>actinobacteria                        Mycobacterium rhodesiae JS60                                                 protein of unknown function DUF159 [Mycobacterium rhodesiae JS60].                                                             <-418047480_?<-418047481_?<-418047482_?||418047483_SRAP*->418047484_?-><-418047485_?<-418047486_?       295395254    SRAP*->                                                                    HMPREF0183_0960                 234   bacteria>actinobacteria                        Brevibacterium mcbrellneri ATCC 49030                                        protein of hypothetical function DUF159 [Brevibacterium mcbrellneri ATCC 49030].                                               <-295395251_?<-295395252_?<-295395253_?||295395254_SRAP*->       291456824    SRAP*->                                                                    -                               230   bacteria>actinobacteria                        Bifidobacterium breve DSM 20213 = JCM 1192                                   putative product YoaM [Bifidobacterium breve DSM 20213 = JCM 1192].                                                            <-291456821_?<-291456822_?<-291456823_?||291456824_SRAP*-><-291456825_?||291456826_?-><-291456827_?       111026950    <-SRAP*                                                                    RHA1_ro11123                    38    bacteria>actinobacteria                        Rhodococcus jostii RHA1                                                      hypothetical protein RHA1_ro11123, partial [Rhodococcus jostii RHA1].                                                          <-111026947_?<-111026948_?||111026949_?-><-111026950_SRAP*||111026951_?->111026952_?-><-111026953_?       336321591    SRAP*->                                                                    -                               247   bacteria>actinobacteria                        [Cellvibrio] gilvus ATCC 13127                                               hypothetical protein Celgi_2492 [[Cellvibrio] gilvus ATCC 13127].                                                              336321588_?->336321589_?-><-336321590_?||336321591_SRAP*-><-336321592_?<-336321593_?||336321594_?->       458842363    SRAP*->?-><-?||Sigma->                                                     H074_05284                      252   bacteria>actinobacteria                        Amycolatopsis decaplanina DSM 44594                                          hypothetical protein H074_05284 [Amycolatopsis decaplanina DSM 44594].                                                         <-458842360_?<-458842361_?<-458842362_?||458842363_SRAP*->458842364_?-><-458842365_?||458842366_Sigma->       385681306    SRAP*->?-><-?||Sigma->                                                     AATC3_020100035513              256   bacteria>actinobacteria                        Amycolatopsis sp. ATCC 39116                                                 hypothetical protein AATC3_35513 [Amycolatopsis sp. ATCC 39116].                                                               <-385681303_?<-385681304_?<-385681305_?||385681306_SRAP*->385681307_?-><-385681308_?||385681309_Sigma->       359765400    SRAP*->                                                                    GOPIP_021_00210                 249   bacteria>actinobacteria                        Gordonia polyisoprenivorans NBRC 16320                                       hypothetical protein GOPIP_021_00210 [Gordonia polyisoprenivorans NBRC 16320].                                                 <-359765397_?<-359765398_?||359765399_?->359765400_SRAP*-><-359765401_?<-359765402_?<-359765403_?       427390419    -                                                                          -                               129   bacteria>actinobacteria                        Actinobaculum massiliae ACS-171-V-Col2                                       hypothetical protein HMPREF9233_00328 [Actinobaculum massiliae ACS-171-V-Col2].                                                       367471361    SRAP*->                                                                    PAI11_43510                     294   bacteria>actinobacteria                        Patulibacter sp. I11                                                         Protein of unknown function DUF159 [Patulibacter sp. I11].                                                                     367471361_SRAP*->367471362_?->367471363_?->367471364_?->       357388131    <-SRAP*                                                                    -                               249   bacteria>actinobacteria                        Kitasatospora setae KM-6054                                                  hypothetical protein KSE_11810 [Kitasatospora setae KM-6054].                                                                  <-357388128_?||357388129_?-><-357388130_?<-357388131_SRAP*||357388132_?->357388133_?->357388134_?->       269795824    SRAP*->                                                                    -                               261   bacteria>actinobacteria                        Sanguibacter keddieii DSM 10542                                              hypothetical protein Sked_25410 [Sanguibacter keddieii DSM 10542].                                                             269795821_?-><-269795822_?<-269795823_?||269795824_SRAP*-><-269795825_?<-269795826_?||269795827_?->       458788059    <-SRAP*                                                                    G352_20671                      235   bacteria>actinobacteria                        Rhodococcus ruber BKS 20-38                                                  hypothetical protein G352_20671 [Rhodococcus ruber BKS 20-38].                                                                 458788056_?->458788057_?-><-458788058_?<-458788059_SRAP*||458788060_?->458788061_?-><-458788062_?       357022826    <-SRAP*                                                                    KEK_22509                       269   bacteria>actinobacteria                        Mycobacterium thermoresistibile ATCC 19527                                   hypothetical protein KEK_22509 [Mycobacterium thermoresistibile ATCC 19527].                                                   357022823_?->357022824_?-><-357022825_?<-357022826_SRAP*||357022827_?->357022828_?-><-357022829_?       328954908    -                                                                          -                               201   bacteria>actinobacteria                        Coriobacterium glomerans PW2                                                 hypothetical protein Corgl_0305 [Coriobacterium glomerans PW2].                                                                       227498228    -                                                                          HMPREF0058_2389                 475   bacteria>actinobacteria                        Actinomyces urogenitalis DSM 15434                                           conserved hypothetical protein, partial [Actinomyces urogenitalis DSM 15434].                                                         383830543    SRAP*->?-><-?<-Sigma                                                       SacxiDRAFT_3077                 264   bacteria>actinobacteria                        S
[truncated: 1,409,484 more chars]
